# Supplementary material for: MCU controls melanoma progression through a redox‐controlled phenotype switch
Source: EMBO Rep. 2022 Sep 26;23(11):e54746. doi: 10.15252/embr.202254746 (PMC9638851; doi:10.15252/embr.202254746)
Supplement: Supplementary file 1 — Expanded View Figures PDF [file EMBR-23-e54746-s008.pdf]

Expanded View Figures

Figure EV1. MCU_A expression affects cancer patient survival (related to Fig 1).

A–O Kaplan–Meier survival plots depicting the correlation between MCU_A mRNA expression levels and survival probability of stage I-II (A) and stage III-IV (B) melanoma patients and of patients with indicated cancer types (C–O). BLCA, bladder urothelial carcinoma; BRCA, breast invasive carcinoma; CESC, cervical squamous cell carcinoma and endocervical adenocarcinoma; COAD, colon adenocarcinoma; GBM, glioblastoma multiforme; HNSC, head and neck squamous cell carcinoma; LIHC, liver hepatocellular carcinoma; OV, ovarian serous cystadenocarcinoma; PRAD, prostate adenocarcinoma; READ, rectum adenocarcinoma; STAD, stomach adenocarcinoma; TGCT, testicular germ cell tumors; THCA, thyroid carcinoma; UCEC, uterine corpus endometrial carcinoma. *P*-values are determined by log-rank test.

P–T MCU expression levels in wild-type versus BRAF-mutant patients (P) and WT versus NRAS-mutant melanoma patients (Q), in melanoma patients with different tumor stages (R), in melanoma patients of different age intervals (S) and in male versus female melanoma patients (T). NOS, not otherwise specified; TPM, transcripts per million reads.

Data information: Data in (P–T) are presented as mean ± SEM (n = 463 patients).

 © 2022 The Authors

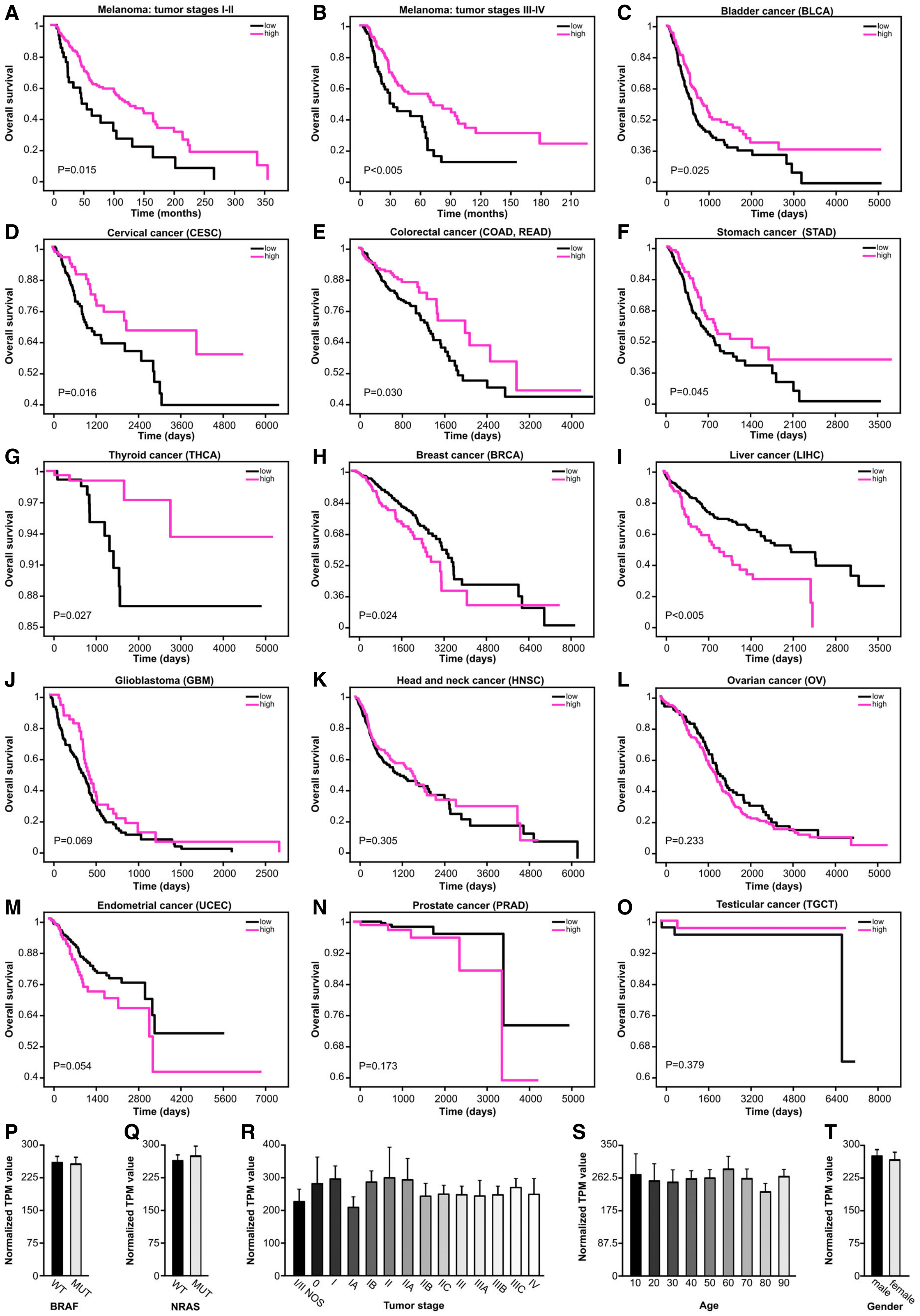
A Melanoma: tumor stages I-II
B Melanoma: tumor stages III-IV
C Bladder cancer (BLCA)
D Cervical cancer (CESC)
E Colorectal cancer (COAD, READ)
F Stomach cancer (STAD)
G Thyroid cancer (THCA)
H Breast cancer (BRCA)
I Liver cancer (LIHC)
J Glioblastoma (GBM)
K Head and neck cancer (HNSC)
L Ovarian cancer (OV)
M Endometrial cancer (UCEC)
N Prostate cancer (PRAD)
O Testicular cancer (TGCT)
low
high
Overall survival
Time (months)
Time (days)
P=0.015
P<0.005
P=0.025
P=0.016
P=0.030
P=0.045
P=0.027
P=0.024
P<0.005
P=0.069
P=0.305
P=0.233
P=0.054
P=0.173
P=0.379
P Q R S T
Normalized TPM value
WT MUT
BRAF
NRAS
I/II NOS 0 I IA IB II IIA IIB IIC III IIIA IIIB IIIC IV
Tumor stage
Age
male female
Gender

Figure EV1.

Figure EV2. Evaluation of siRNA and shRNA-mediated MCU_A depletion efficacy and their influence on store-operated Ca^{2+} entry (related to Fig 2).

A, B mRNA expression of MCU_A in 1205Lu (A) and WM3734 (B) shCTRL and MCU_{A_KD} cells ($n \leq 4$ biological replicates).
C WB for MCU_A in 1205Lu and WM3734 MCU_{A_KD} cells.
D Corresponding band quantification, shown as the ratio between MCU_A and the loading control calnexin ($n = 4$ biological replicates).
E, F mRNA expression of MCU_A in 1205Lu (A) and WM3734 (B) MCU_A transient (siRNA) KD cells ($n \leq 4$ biological replicates).
G WB for MCU_A in 1205Lu and WM3734 transient MCU_{A_KD} cells.
H Corresponding band quantification, shown as the ratio between MCU_A and the loading control calnexin ($n = 3$ biological replicates).
I, J mRNA expression of MCU complex components (MCU_A, MCU_B, MICU1, MICU2, MICU3, EMRE, MCUR1) in 1205Lu ($n \geq 3$ biological replicates) (I) and WM3734 ($n \geq 3$ biological replicates) (J) cells with and without stable MCU_{A_KD}, normalized to housekeeping gene TBP, quantified by RT–qPCR.
K WB for proteins of the MCU complex (MCU_B, MICU1, MICU2, MICU3, MCUR1) in 1205Lu and WM3734 cells with and without stable MCU_{A_KD}. β-actin was used as loading control.
L mRNA expression of MCU complex components (MCU_A, MCU_B, MICU1, MICU2, MICU3, EMRE, and MCUR1) in WM3734 transient MCU_{A_KD} cells, normalized to housekeeping gene TBP, quantified by RT–qPCR ($n = 5$ biological replicates).
M Cytosolic Ca^{2+} measurements in stable WM3734 shCTRL (black), WM3734 shMCU_1 (darker orange) and WM3734 shMCU_2 (lighter orange) cells.
N Corresponding quantification of basal cytosolic Ca^{2+} before thapsigargin (Tg, 1 μM) stimulation, of ΔCa^{2+} ER and of ΔSOCE (shCTRL: $n = 186$ cells from seven biological replicates; shMCU_1: $n = 133$ cells from six biological replicates; shMCU_2: $n = 167$ cells from seven biological replicates).

Data information: Data are presented as mean ± SEM. Statistical significance was assessed using unpaired, two-tailed Student's *t*-test (KD cells were compared with their respective control), *$P < 0.05$; **$P < 0.01$; ***$P < 0.005$; no asterisk means no statistical significance ($P > 0.05$).
Source data are available online for this figure.

 © 2022 The Authors

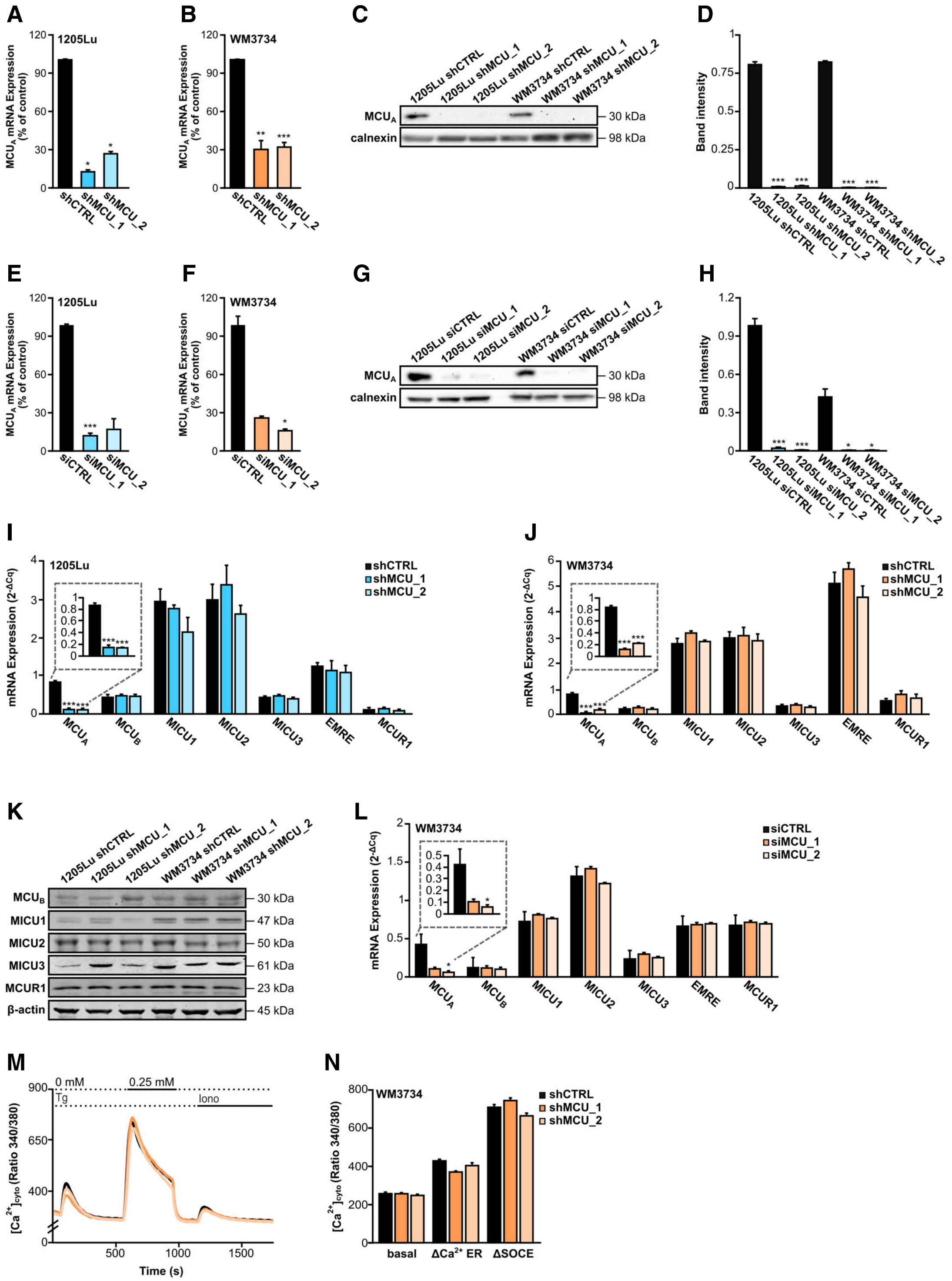
A
1205Lu
MCUA mRNA Expression (% of control)
shCTRL
shMCU_1
shMCU_2
B
WM3734
C
1205Lu shCTRL
1205Lu shMCU_1
1205Lu shMCU_2
WM3734 shCTRL
WM3734 shMCU_1
WM3734 shMCU_2
MCUA
calnexin
30 kDa
98 kDa
D
Band intensity
E
siCTRL
siMCU_1
siMCU_2
F
G
H
I
mRNA Expression (2-ΔCq)
MCUA
MCUB
MICU1
MICU2
MICU3
EMRE
MCUR1
J
K
MCUB
MICU1
MICU2
MICU3
MCUR1
β-actin
47 kDa
50 kDa
61 kDa
23 kDa
45 kDa
L
M
0 mM
0.25 mM
Tg
Iono
[Ca2+]cyto (Ratio 340/380)
Time (s)
N
basal
ΔCa2+ ER
ΔSOCE

Figure EV2.

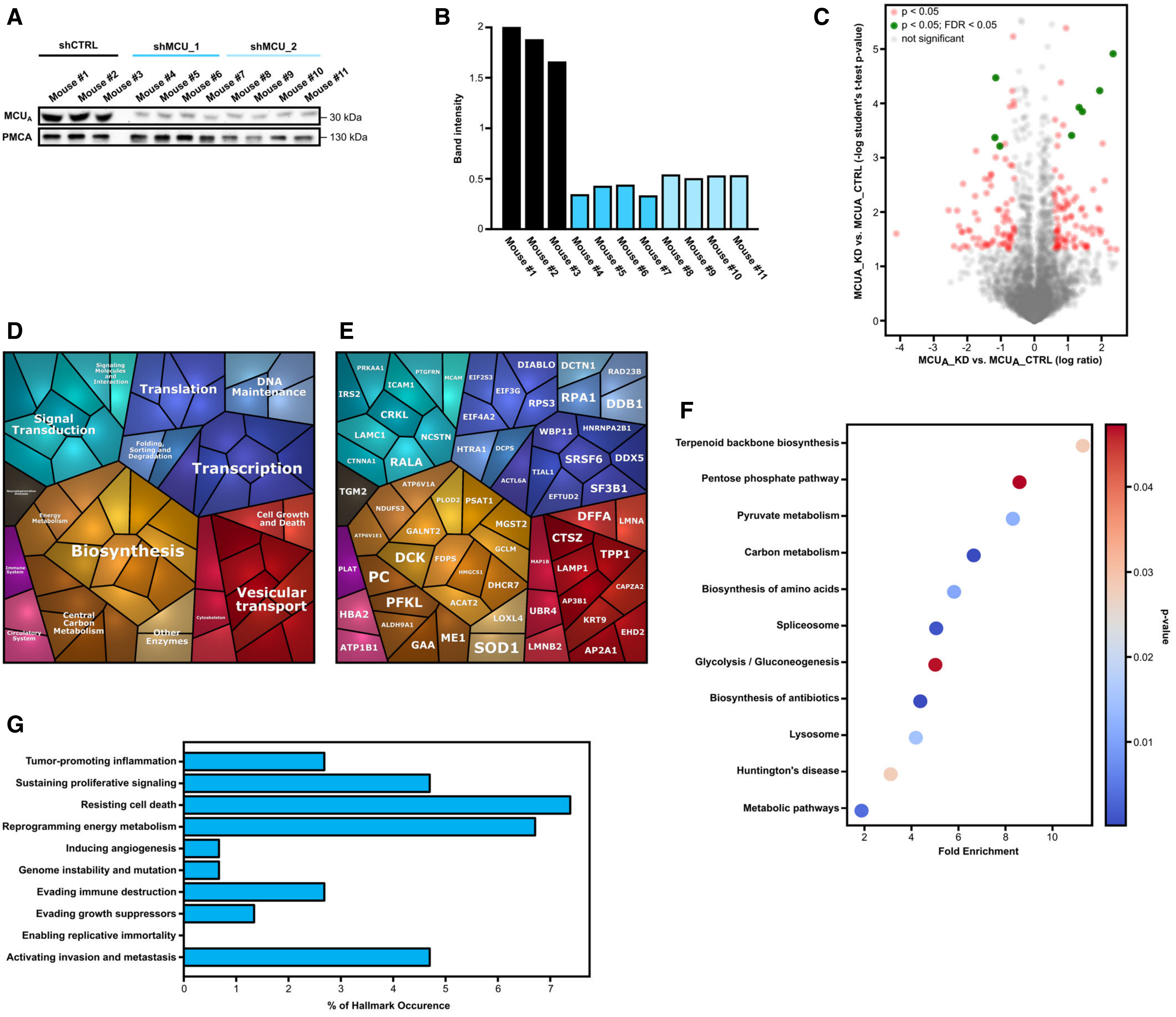

Figure EV3. MCU_A controls metabolic signaling in 1205Lu melanoma cells (related to Figs 3 and 4).

A, B Validation of MCU_A knockdown in melanoma xenografts. WB for MCU_A in 1205Lu MCU_{A_KD} cells isolated from xenograft tumors (A). Corresponding band intensity, shown as the ratio between MCU_A and the loading control plasma membrane Ca^{2+} ATPase (PMCA) (B).

C Volcano plot of proteomics data for 1205Lu MCU_{A_KD} cells versus MCU_A_CTRL. Protein entities with significant *P*-values ($P < 0.05$) are marked with red dots. Protein entities with significance in both *P*-value and FDR (false discovery rate) ($P < 0.05$; FDR < 0.05) are marked with green dots.

D, E Proteomap analyses with protein entities that display significant difference (absolute Log2 fold change > 0.5 and $P < 0.05$) between 1205Lu MCU_{A_KD} and 1205Lu shCTRL cells.

F KEGG-based analysis of cellular components and processes based on protein hits that show differential expression between in 1205Lu MCU_{A_KD} and shCTRL cells, revealed via proteomics.

G Cancer hallmark-based enrichment analysis of proteins differentially expressed in 1205Lu MCU_{A_KD} versus shCTRL cells, based on proteomics data shown in (A). Percentage of hits in the specific gene set is displayed.

Source data are available online for this figure.

 © 2022 The Authors

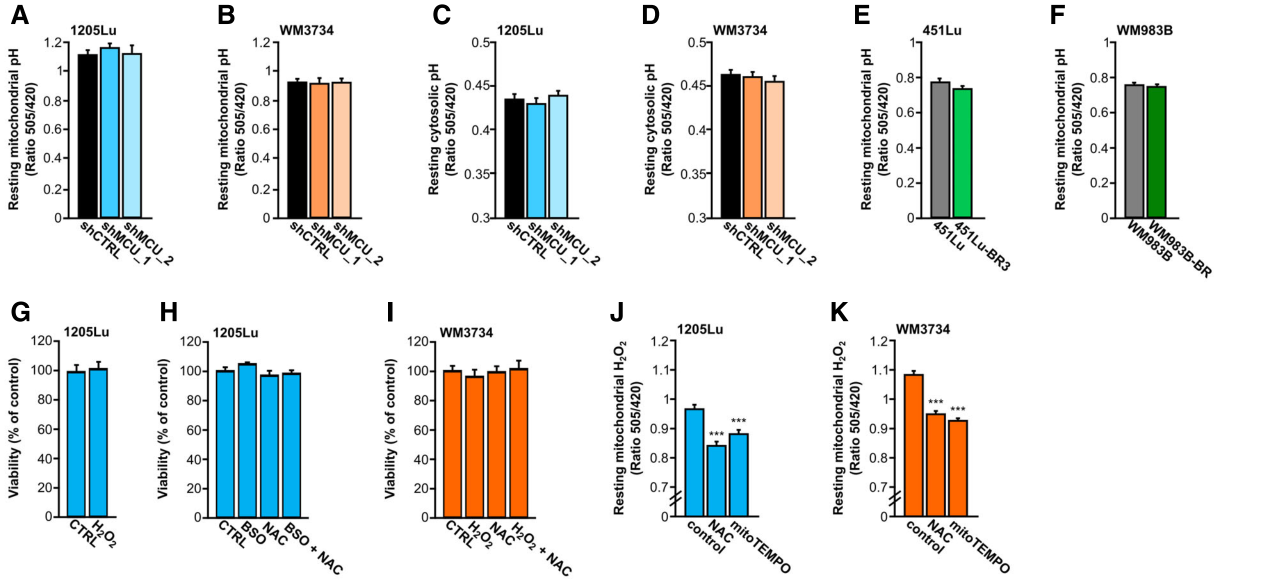

Figure EV4. Effect of redox agents on melanoma cell viability and mitochondrial ROS (related to Fig 5).

A, B Quantification of basal mito-SypHer ratios (pH control for mito-HyPer) in 1205Lu (A) and WM3734 (B) with and without stable MCU_{A_KD} (1205Lu—shCTRL: $n = 210$ cells from six biological replicates; shMCU_1: $n = 174$ cells from six biological replicates; shMCU_2: $n = 117$ cells from six biological replicates; WM3734—shCTRL: $n = 74$ from three biological replicates; shMCU_1: $n = 51$ cells from three biological replicates; shMCU_2: $n = 67$ cells from three biological replicates).

C, D Quantification of basal SypHer ratios (pH control for HyPer) in 1205Lu (C) and WM3734 (D) with and without stable MCU_{A_KD} (1205Lu—shCTRL: $n = 149$ cells from four biological replicates; shMCU_1: $n = 176$ cells from four biological replicates; shMCU_2: $n = 158$ cells from four biological replicates; WM3734—shCTRL: $n = 187$ cells from six biological replicates; shMCU_1: $n = 189$ cells from six biological replicates; shMCU_2: $n = 193$ cells from six biological replicates).

E, F Quantification of basal mito-SypHer ratios (pH control for mito-HyPer) in 451Lu (E) and WM983B (F) and their respective BRAF inhibitor-resistant versions (451Lu: $n = 73$ cells from four biological replicates; 451Lu-BR3: $n = 136$ cells from four biological replicates; WM983B: $n = 103$ cells from four biological replicates; WM983B-BR: $n = 91$ cells from four biological replicates).

G Viability assay upon 100 μM H_2O_2 treatment for 24 h in 1205Lu melanoma cells ($n = 4$ biological replicates).

H Viability assay upon 4-h treatment with 1 mM BSO, 200 μM NAC and 1 mM BSO + 200 μM NAC in 1205Lu melanoma cells ($n = 12$ wells from three biological replicates).

I Viability assay upon 4-h treatment with 100 μM H_2O_2, 200 μM NAC and 100 μM H_2O_2 + 200 μM NAC in WM3734 melanoma cells ($n = 12$ wells from three biological replicates).

J, K Resting mitochondrial hydrogen peroxide (H_2O_2; mito-HyPer) in 1205Lu (J) and WM3734 (K) with and without overnight pre-treatment with NAC (200 μM) or mito-TEMPO (1 μM) (1205Lu—control: $n = 191$ cells from 3 biological replicates; NAC: $n = 148$ cells from three biological replicates; mitoTEMPO: $n = 133$ cells from three biological replicates; WM3734—control: $n = 237$ cells from four biological replicates; NAC: $n = 263$ cells from five biological replicates; mitoTEMPO: $n = 255$ cells from three biological replicates).

Data information: (A–F) and (J–K) were measured in Ringer's buffer containing 0.5 mM Ca^{2+}. Data are presented as mean ± SEM. Statistical significance was determined using unpaired, two-tailed Student's *t*-test, ***$P < 0.005$; no asterisk means no statistical significance ($P > 0.05$).

© 2022 The Authors

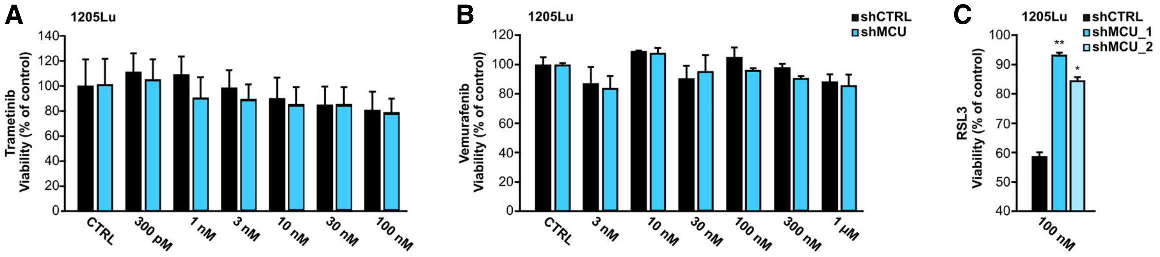

Figure EV5. MCU_A controls melanoma cell therapeutic sensitivity (related to Fig 7).

A, B 1205Lu shCTRL and MCU_{A_KD} cell viability following incubation with different concentrations of trametinib for 144 h (A) and vemurafenib for 96 h (B). Data are shown as percent of control (untreated cells = CTRL) ($n = 3$ biological replicates).

C 1205Lu cell viability upon incubation with 100 nM RSL3 for 72 h. Data are shown as percent of control (untreated cells, not shown) ($n \leq 8$ biological replicates). RSL, RAS-selective lethal.

Data information: Data are presented as mean ± SEM. Statistical significance was determined using unpaired, two-tailed Student's *t*-test, $*P < 0.05$; $**P < 0.01$; no asterisk means no statistical significance ($P > 0.05$).

© 2022 The Authors
