# Supplementary material for: MCU controls melanoma progression through a redox‐controlled phenotype switch
Source: EMBO Rep. 2022 Sep 26;23(11):e54746. doi: 10.15252/embr.202254746 (PMC9638851; doi:10.15252/embr.202254746)
Supplement: Supplementary file 2 — Table EV1 [file EMBR-23-e54746-s007.docx]

# **Table EV1. siRNA sequences**

| **siRNA** | **Target** | **Concentration** | **Sequence 5’-3’** | **Company** |
| --- | --- | --- | --- | --- |
| MCU_1 | MCU | 20 µM | CAGGTGCCTTGCAAAGGTTGA | Qiagen |
| MCU_2 | MCU | 20 µM | CTGGTCATTAATGACTTAACA | Qiagen |
| Non- silencing | (control) | 20 µM | AATTCTCCGAACGTGTCACGT | Qiagen |
