# Supplementary material for: MCU controls melanoma progression through a redox‐controlled phenotype switch
Source: EMBO Rep. 2022 Sep 26;23(11):e54746. doi: 10.15252/embr.202254746 (PMC9638851; doi:10.15252/embr.202254746)
Supplement: Supplementary file 3 — Table EV2 [file EMBR-23-e54746-s004.docx]

# **Table EV2. Plasmids and constructs**

| **Plasmid/construct** | **Provider/ Company** |
| --- | --- |
| MCU-Flag | R. Rizzuto |
| MCU-eGFP | R. Rizzuto |
| mito-Grx1-roGFP2 | T. Dick |
| pcDNA-4mt-D_3_cpV | P. Lipp (#36324, Addgene) |
| pC1-HyPer-3 | V. V. Belousov (#42131, Addgene) |
| pC1-HyPer-C199S | V. V. Belousov (#42213, Addgene) |
| pHyPer-2-dMito | V. V. Belousov (#FP942, Evrogen) |
| pSypHer-dMito | V. V. Belousov (#48251, Addgene) |
| pcDNA-mit-AT1.03 (mito-ATEAM) | H. Noji (Imamura, Nhat et al., 2009) |
