# Supplementary material for: MCU controls melanoma progression through a redox‐controlled phenotype switch
Source: EMBO Rep. 2022 Sep 26;23(11):e54746. doi: 10.15252/embr.202254746 (PMC9638851; doi:10.15252/embr.202254746)
Supplement: Supplementary file 4 — Table EV3 [file EMBR-23-e54746-s012.docx]

# **Table EV3. Sequences of gene-specific primers**

| **Target** | **Forward-Primer 5’-3’** | **Reverse-Primer 5’-3’** |
| --- | --- | --- |
| MCU/CCDC109a | CACACAGTTTGGCATTTTGG | TGTCTGTCTCTGGCTTCTGG |
| MCUb / CCDC109b | CAAAACTGCAGCCATCTTCA | CTCTTTGGACACTGCACATCA |
| MICU1 / CBARA1 | GTGTTCAGCCCTCACAACCT | CCACCAAACTGCCTCTCAGT |
| MICU2 / EFHA1 | AGCGCTTCATGCAGTTTTCT | CAGCTGTTTGGATCCCTGAC |
| MICU3 / EFHA2 | CCAGTTTGGAAAGGCTCATC | ATTCTGAACCCTGCATGTGG |
| MCUR1 / CCDC90a | GCCTGCACTCGTTTCCTG | CTGCTCCCAGAAGAGGTGAA |
| EMRE / SMDT1 | CTTGAGGAAAGATGGCGATG | CGACATAGAGAAAGGGGATCA |
| RNA-Polymerase | GGAGATTGAGTCCAAGTTCA | GCAGACACACCAGCATAGT |
| TBP | CGGAGAGTTCTGGGATTGT | GGTTCGTGGCTCTCTTATC |
