# Supplementary material for: MCU controls melanoma progression through a redox‐controlled phenotype switch
Source: EMBO Rep. 2022 Sep 26;23(11):e54746. doi: 10.15252/embr.202254746 (PMC9638851; doi:10.15252/embr.202254746)
Supplement: Supplementary file 5 — Table EV4 [file EMBR-23-e54746-s010.docx]

# **Table EV4. Primary antibodies for immunoblotting and immunohistochemistry**

| **Primary antibody** | **Clonality** | **Species** | **Company** | **Product number** | **Dilution** |
| --- | --- | --- | --- | --- | --- |
| β-actin | monoclonal | Mouse | Sigma-Aldrich | A5441 | 1:10,000 (WB) |
| Calnexin | polyclonal | Rabbit | Enzo | ADI-SPA-860-F | 1:1,000 (WB) |
| MCU | polyclonal | Rabbit | Sigma-Aldrich | HPA016480 | 1:500 (WB) |
|  | monoclonal | Rabbit | Cell Signaling | 14997 | 1:500 (WB), 1:200 (IHC) |
| MCU_B_ | polyclonal | Rabbit | Abgent | AP12355b | 1:500 (WB) |
| CBARA1/MICU1 | monoclonal | Rabbit | Cell Signaling | 12524S | 1:500 (WB) |
| MICU2 | polyclonal | Rabbit | Abcam | ab101465 | 1:500 (WB) |
| MICU3 | polyclonal | Rabbit | Sigma-Aldrich | HPA024779 | 1:500 (WB) |
| MCUR1 | polyclonal | Rabbit | Cell Signaling | 13706S | 1:500 (WB) |
| PMCA | monoclonal | Mouse | Thermo Fisher | MA1-914 | 1:1,000 (WB) |
| TOMM20 coupled to Alexa Fluor 488 | monoclonal | Rabbit | Abcam | ab205486 | 1:100 (IHC) |
