# Supplementary material for: MCU controls melanoma progression through a redox‐controlled phenotype switch
Source: EMBO Rep. 2022 Sep 26;23(11):e54746. doi: 10.15252/embr.202254746 (PMC9638851; doi:10.15252/embr.202254746)
Supplement: Supplementary file 6 — Table EV5 [file EMBR-23-e54746-s009.docx]

# **Table EV5. Secondary antibodies for immunoblotting**

| **Secondary antibody** | **Company** | **Product number** | **Dilution** |
| --- | --- | --- | --- |
| α-Rabbit | GE Healthcare | NA9340 | 1:25,000 |
|  | LI-COR | 926-32213 | 1:10,000 |
| α-Mouse | Amersham Bioscience | NA931 | 1:5,000 |
