# Supplementary material for: MCU controls melanoma progression through a redox‐controlled phenotype switch
Source: EMBO Rep. 2022 Sep 26;23(11):e54746. doi: 10.15252/embr.202254746 (PMC9638851; doi:10.15252/embr.202254746)
Supplement: Supplementary file 11 — Source Data for Expanded View [file EMBR-23-e54746-s013.zip › EMBR_2246_Figure EV2_full blots.pdf]

Figure EV2 C: Uncropped western blot images

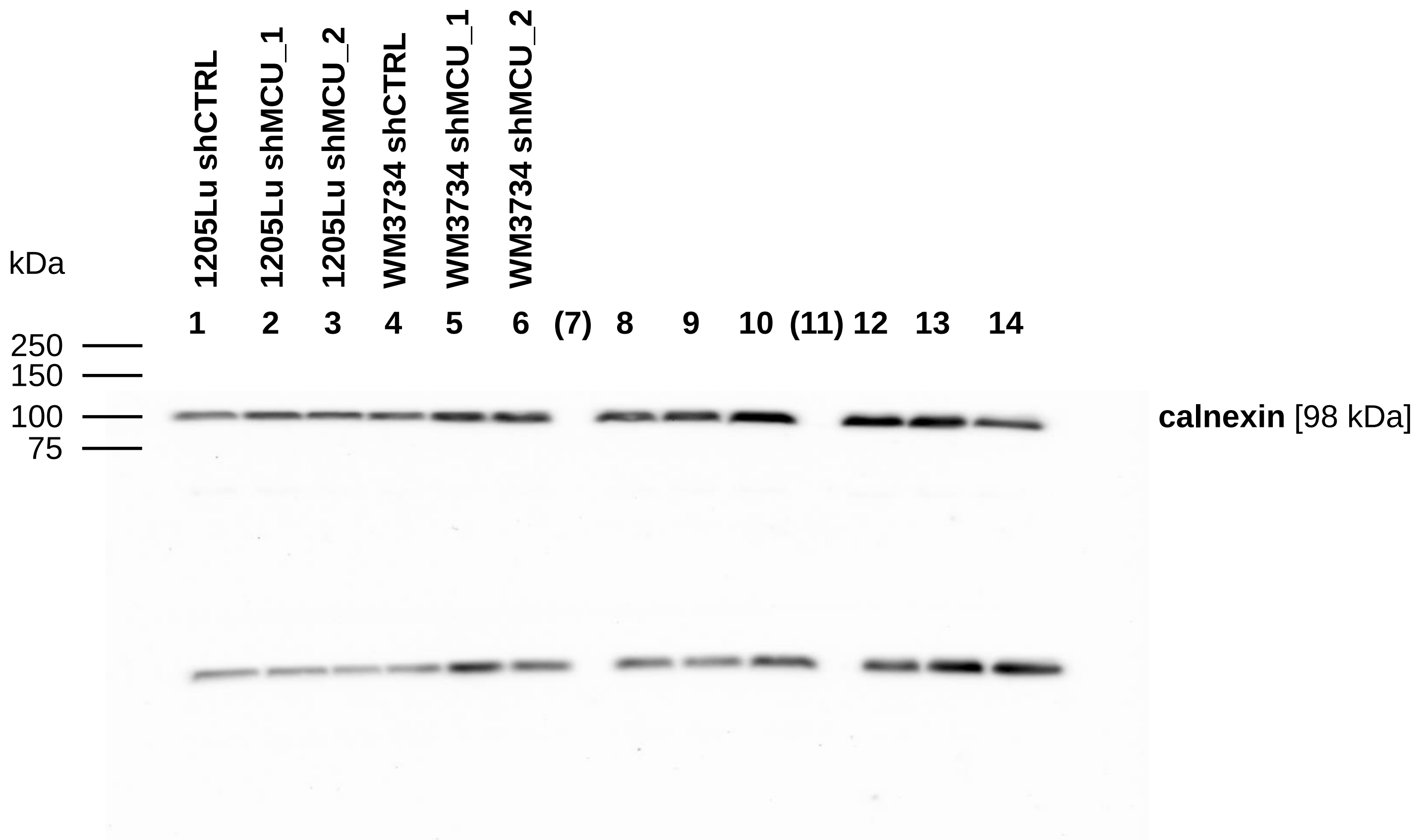

Blot #1. Lysates from 1205Lu and WM3734 with and without MCU_A stable KD (indicated above) were loaded on lanes 1-6. The rest of the samples were not used in the manuscript. The blot was performed for the detection of two proteins, however only one (calnexin) was included in the manuscript. Calnexin is a 98 kDa protein which served as a loading control. Molecular size is indicated on the left side.

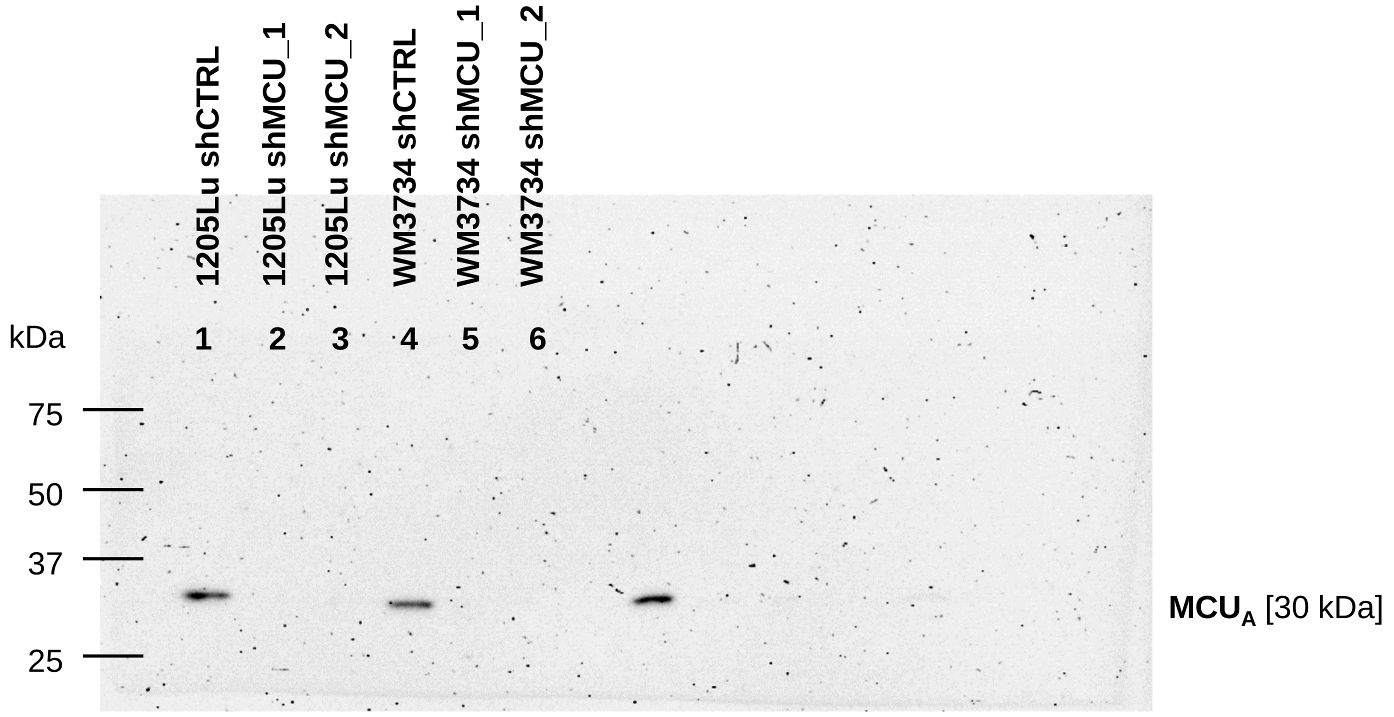

Blot #2. Lysates from 1205Lu and WM3734 with and without MCU_A stable KD (indicated above) were loaded on lanes 1-6. The rest of the samples were not used in the manuscript. The blot was performed for the detection of MCU_A (a 30 kDa protein) in order to confirm MCU_A KD. Molecular size is indicated on the left side.

Figure EV2 G: Uncropped western blot images

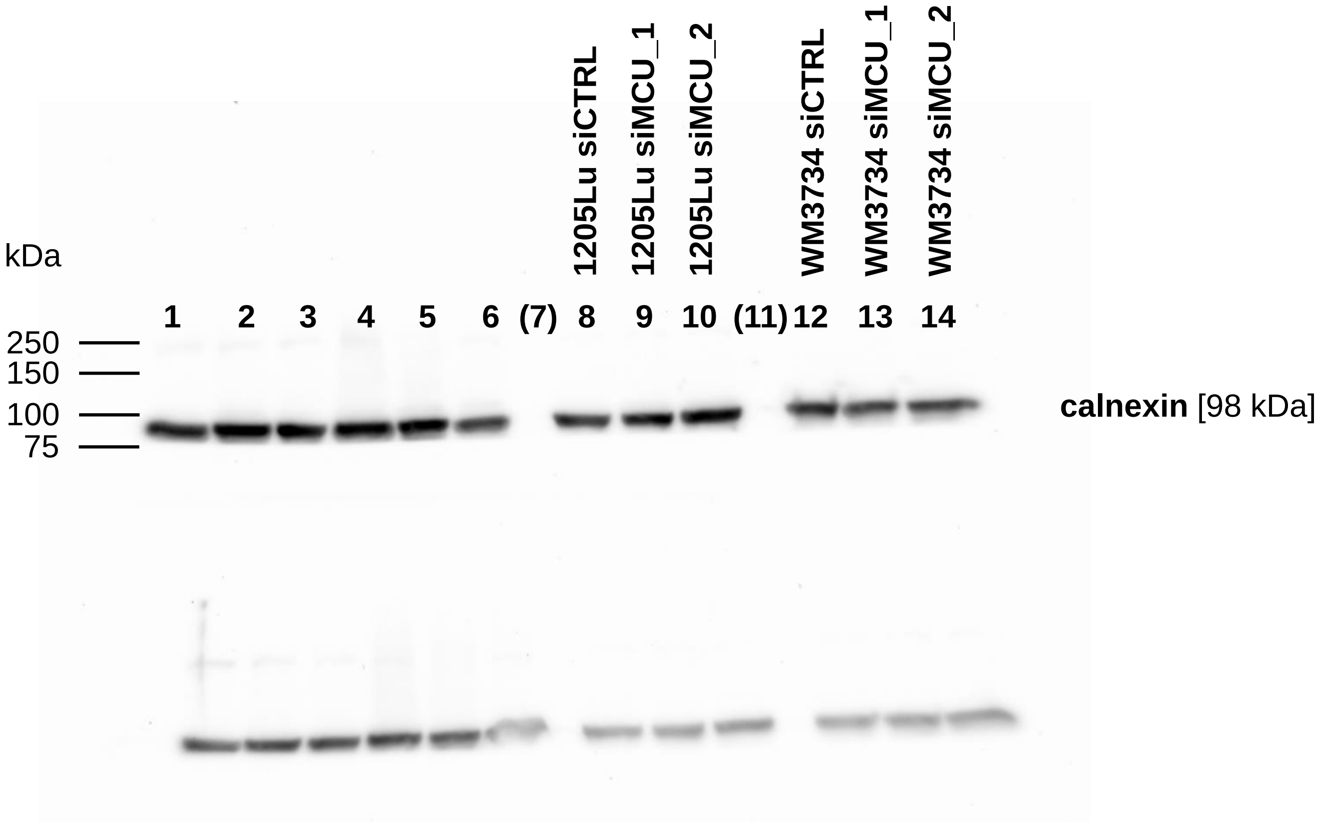

Blot #1. Lysates from 1205Lu and WM3734 with and without MCU_A transient KD (indicated above) were loaded on lanes 8-10 and 12-14. The rest of the samples were not used in the manuscript. The blot was performed for the detection of two proteins, however only one (calnexin) was included in the manuscript. Calnexin is a 98 kDa protein which served as a loading control. Molecular size is indicated on the left side.

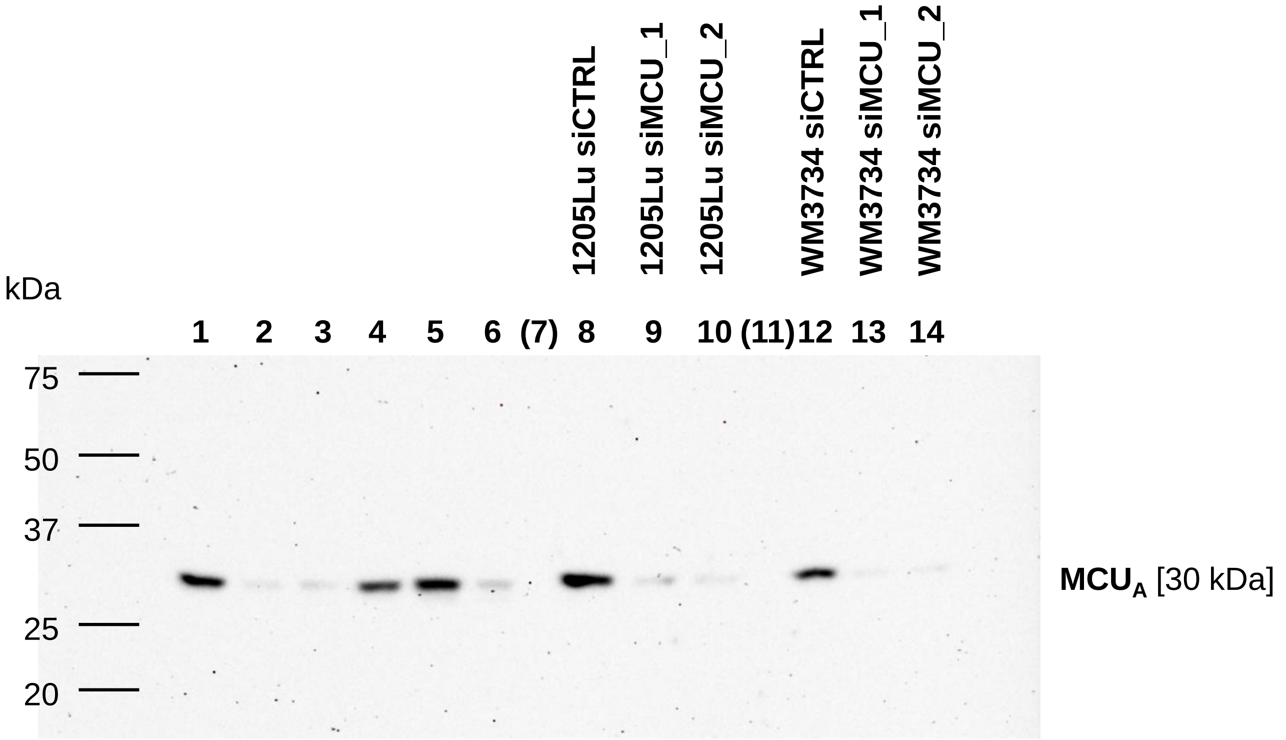

Blot #2. Lysates from 1205Lu and WM3734 with and without MCU_A transient KD (indicated above) were loaded on lanes 1-6. The rest of the samples were not used in the manuscript. The blot was performed for the detection of MCU (a 30 kDa protein) in order to confirm MCU_A KD. Molecular size is indicated on the left side.

Figure EV2 K: Uncropped western blot images

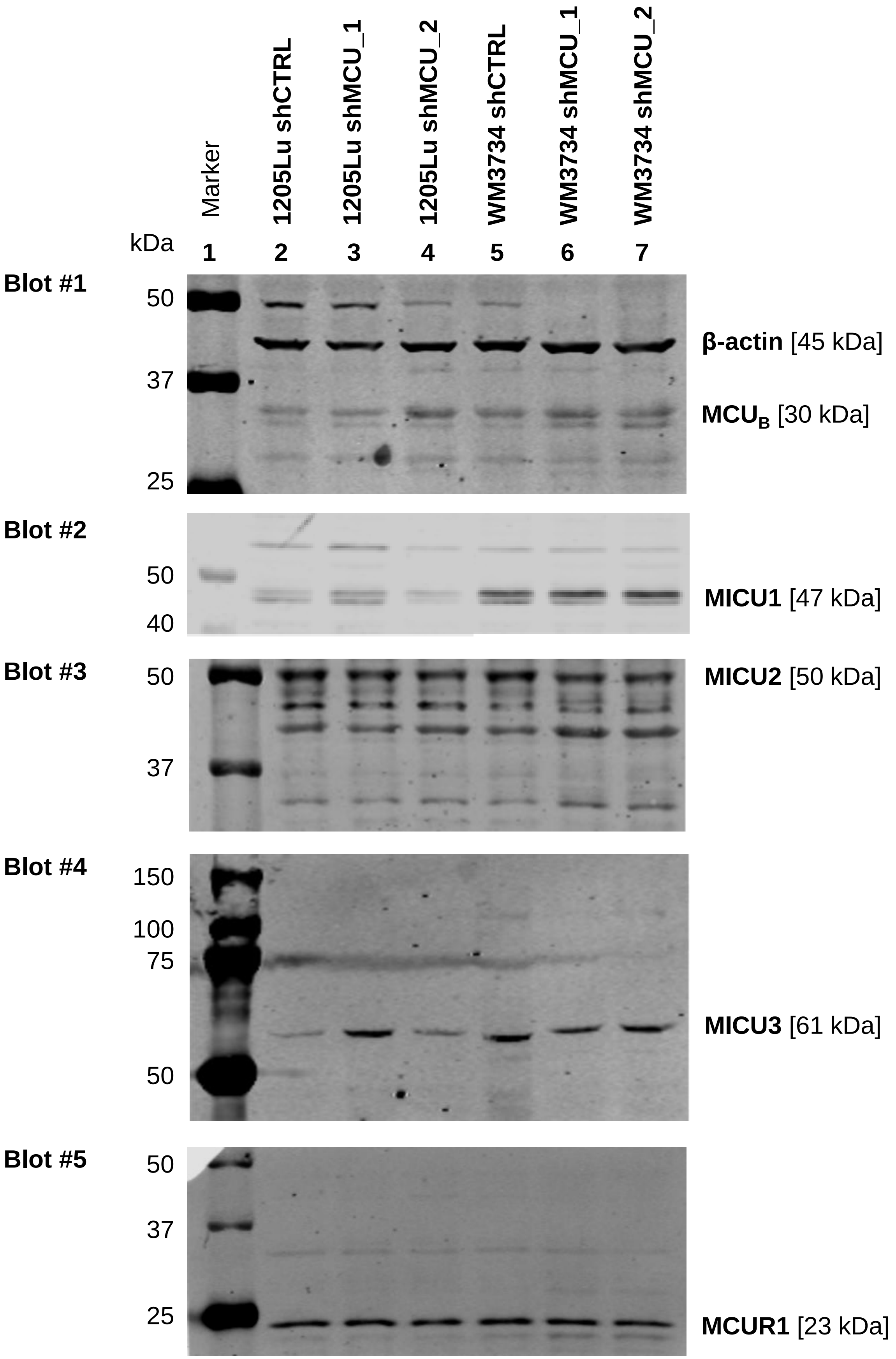

Blots #1-5. Lysates from 1205Lu and WM3734 with and without MCU stable KD (indicated above) were loaded on lanes 2-7. The blot was performed for the detection of MCU$_B$, MICU1, MICU2, MICU3 and MCUR1. β-actin was used as loading control. Molecular size is indicated by the marker on the left side (lane 1).
