# Supplementary material for: MCU controls melanoma progression through a redox‐controlled phenotype switch
Source: EMBO Rep. 2022 Sep 26;23(11):e54746. doi: 10.15252/embr.202254746 (PMC9638851; doi:10.15252/embr.202254746)
Supplement: Supplementary file 11 — Source Data for Expanded View [file EMBR-23-e54746-s013.zip › EMBR_2246_Figure EV3_full blots.pdf]

Figure EV3 A: Uncropped western blot images

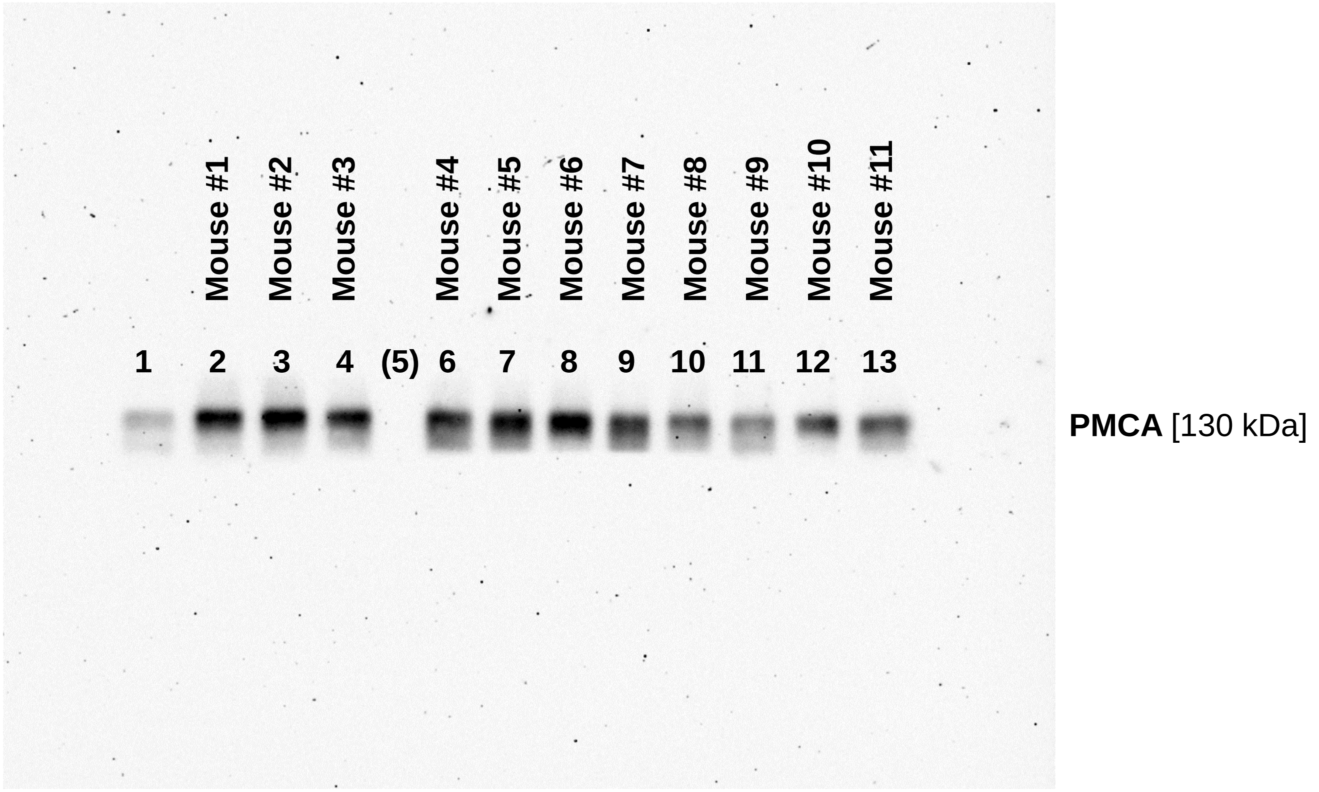

Blot #1. Lysates from 1205Lu with (Mice 4-11) and without MCU stable KD (Mice 1-3) were loaded on lanes 2-4 and 6-13, respectively. The rest of the samples were not used in the manuscript. The blot was performed for the detection of PMCA, a 130 kDa protein which served as a loading control.

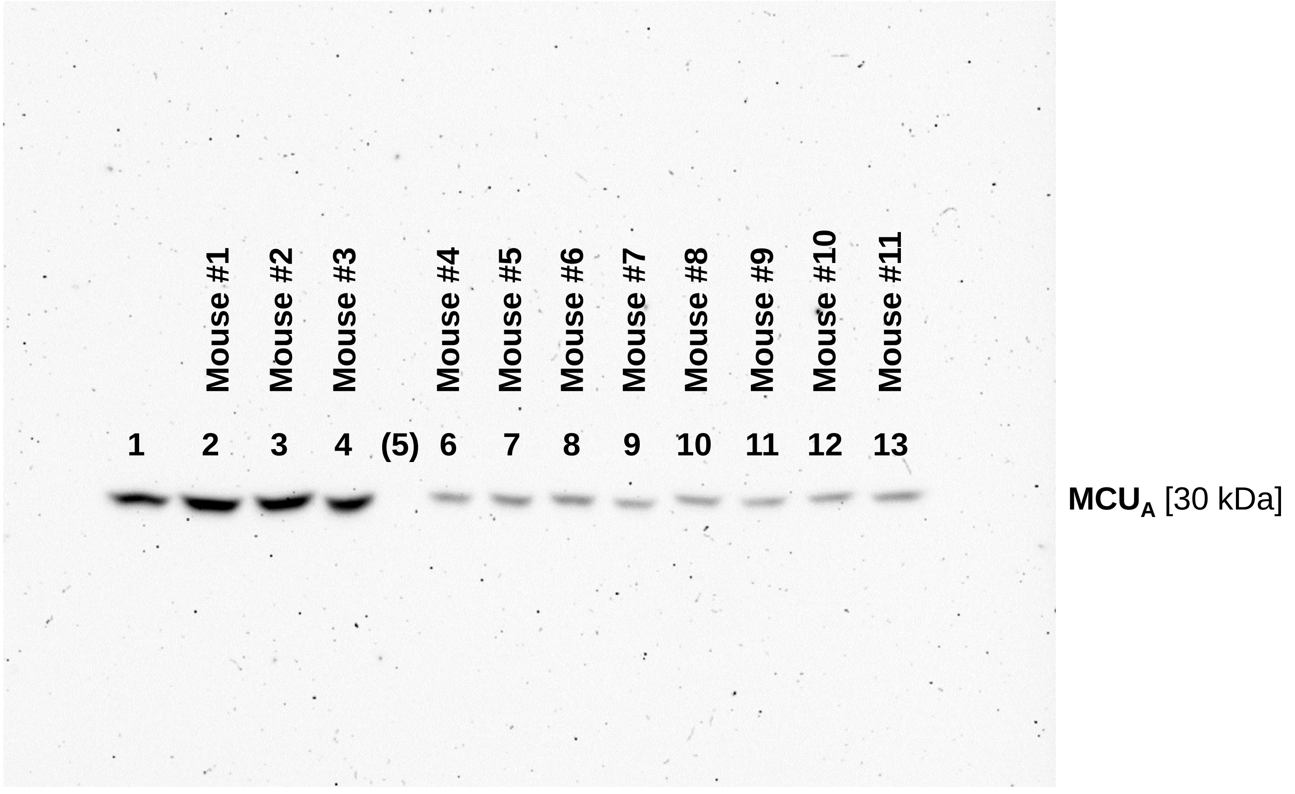

Blot #2. Lysates from 1205Lu with (Mice 4-11) and without MCU stable KD (Mice 1-3) were loaded on lanes 2-4 and 6-13, respectively. The rest of the samples were not used in the manuscript. The blot was performed for the detection of MCU (a 30 kDa protein) in order to confirm MCU_A KD *in vivo*.
