# Supplementary material for: MCU controls melanoma progression through a redox‐controlled phenotype switch
Source: EMBO Rep. 2022 Sep 26;23(11):e54746. doi: 10.15252/embr.202254746 (PMC9638851; doi:10.15252/embr.202254746)
Supplement: Supplementary file 12 — PDF+ [file EMBR-23-e54746-s011.pdf]

Article

SOURCE DATA TRANSPARENT PROCESS OPEN ACCESS

EMBO reports

MCU controls melanoma progression through a redox-controlled phenotype switch

Ioana Stejerean-Todoran[1,†], Katharina Zimmermann[2,†], Christine S Gibhardt[1,†], Adina Vultur[1,3], Christian Ickes[1], Batool Shannan[3,4], Zuriñe Bonilla del Rio[1], Anna Wölling[5], Sabrina Cappello[1], Hsu-Min Sung[1], Magdalena Shumanska[1], Xin Zhang[1], Maithily Nanadikar[6], Muhammad U Latif[7], Anna Wittek[8,9], Felix Lange[8,9], Andrea Waters[3], Patricia Brafford[3], Jörg Wilting[10], Henning Urlaub[11,12], Dörthe M Katschinski[6], Peter Rehling[13], Christof Lenz[11,12], Stefan Jakobs[8,9], Volker Ellenrieder[7], Alexander Roesch[4], Michael P Schön[5], Meenhard Herlyn[3], Hedwig Stanisz[5] & Ivan Bogeski[1,*]

Abstract

Melanoma is the deadliest of skin cancers and has a high tendency to metastasize to distant organs. Calcium and metabolic signals contribute to melanoma invasiveness; however, the underlying molecular details are elusive. The MCU complex is a major route for calcium into the mitochondrial matrix but whether MCU affects melanoma pathobiology was not understood. Here, we show that MCU_A expression correlates with melanoma patient survival and is decreased in BRAF kinase inhibitor-resistant melanomas. Knockdown (KD) of MCU_A suppresses melanoma cell growth and stimulates migration and invasion. In melanoma xenografts, MCU_{A_KD} reduces tumor volumes but promotes lung metastases. Proteomic analyses and protein microarrays identify pathways that link MCU_A and melanoma cell phenotype and suggest a major role for redox regulation. Antioxidants enhance melanoma cell migration, while prooxidants diminish the MCU_{A_KD}-induced invasive phenotype. Furthermore, MCU_{A_KD} increases melanoma cell resistance to immunotherapies and ferroptosis. Collectively, we demonstrate that MCU_A controls melanoma aggressive behavior and therapeutic sensitivity. Manipulations of mitochondrial calcium and redox homeostasis, in combination with current therapies, should be considered in treating advanced melanoma.

Keywords calcium; MCU; melanoma; mitochondria; ROS
Subject Categories Cancer; Membrane & Trafficking; Metabolism
DOI 10.15252/embr.202254746 | Received 28 January 2022 | Revised 29 August 2022 | Accepted 29 August 2022 | Published online 26 September 2022
EMBO Reports (2022) 23: e54746

Introduction

Melanoma is the most aggressive of all skin cancers (Schadendorf *et al*, 2018). Despite recent therapy improvements, advanced melanoma is difficult to cure and many patients still succumb to the disease; this is mostly influenced by low response rates to immunotherapies and drug resistance which commonly occur due to the high heterogeneity and plasticity of melanoma cells (Ribas & Wolchok, 2018; O'Donnell *et al*, 2019; Jenkins & Fisher, 2020).

Mitochondria were identified as important regulators of melanoma pathobiology (Haq *et al*, 2013; Roesch *et al*, 2013; Theodosakis *et al*, 2014; Zhang *et al*, 2019). Moreover, mitochondria are essential determinants of melanoma response to targeted therapies and immune checkpoint blockade (Vazquez *et al*, 2013; Harel *et al*, 2019). It is also becoming increasingly apparent that the tight interplay between mitochondria, bioenergetics, and reactive oxygen

1 Molecular Physiology, Department of Cardiovascular Physiology, University Medical Center, Georg-August-University, Göttingen, Germany
2 Biophysics, CIPMM, Saarland University, Homburg, Germany
3 The Wistar Institute, Melanoma Research Center, Philadelphia, PA, USA
4 Department of Dermatology, University Hospital Essen, West German Cancer Center, University Duisburg-Essen and the German Cancer Consortium (DKTK)
5 Department of Dermatology, Venereology and Allergology, University Medical Center, Georg-August-University, Göttingen, Germany
6 Department of Cardiovascular Physiology, University Medical Center Göttingen, Georg-August-University, Göttingen, Germany
7 Department of Gastroenterology, Gastrointestinal Oncology and Endocrinology, University Medical Center Göttingen, Göttingen, Germany
8 Department of NanoBiophotonics, Max Planck Institute for Multidisciplinary Sciences, Göttingen, Germany
9 Clinic of Neurology, University Medical Center Göttingen, Göttingen, Germany
10 Department of Anatomy and Cell Biology, University Medical Center, Georg-August-University, Göttingen, Germany
11 Bioanalytical Mass Spectrometry Group, Max Planck Institute for Multidisciplinary Sciences, Göttingen, Germany
12 Bioanalytics, Institute of Clinical Chemistry, University Medical Center Göttingen, Göttingen, Germany
13 Department of Cellular Biochemistry, University Medical Center Göttingen, GZMB, Göttingen, Germany
*Corresponding author. Tel: +0551 39 65520; Fax: +0551 39 5895; E-mail: ivan.bogeski@med.uni-goettingen.de
[†]These authors contributed equally to this work

© 2022 The Authors. Published under the terms of the CC BY 4.0 license.

species (ROS), plays an important role in melanoma biology and therapeutic sensitivity (Roesch *et al*, 2013; Piskounova *et al*, 2015; Chio & Tuveson, 2017). Our previous findings showed that highly aggressive, tumor-maintaining, and therapy-resistant melanoma cells rely on mitochondrial bioenergetic output (Roesch *et al*, 2010, 2013; Cappello *et al*, 2021). Other studies also demonstrated that increased mitochondrial capacity and resistance to oxidative stress define aggressive melanoma cell subsets (Haq *et al*, 2013; Vazquez *et al*, 2013) and that antioxidants and the environmental redox status control melanoma but also lung cancer cell metastatic spread (Sayin *et al*, 2014; Le Gal *et al*, 2015; Piskounova *et al*, 2015; Ubellacker *et al*, 2020). In addition, inhibitors of the antioxidant enzyme thioredoxin reductase 1, which is elevated in cancer, efficiently eliminated cancer cells including melanoma without affecting healthy cells (Stafford *et al*, 2018).

Similar to the redox signals, ionic Ca^{2+} is an important regulator of cancer cell biology (Prevarskaya *et al*, 2011; Hoth, 2016; Marchi *et al*, 2020). In mitochondria, the mitochondrial Ca^{2+} uniporter (MCU) complex is the main transporter of Ca^{2+} across the inner mitochondrial membrane (IMM). The MCU complex is composed of several proteins located within the IMM or in the mitochondrial intermembrane space (IMS) (Fig 1A). These include the bona fide channel pore MCU_A, its inhibiting isoform MCU_B, EMRE, MCUR1, and the three regulators MICU1-3, which sense the Ca^{2+} concentration in the IMS and thus control the activity of the whole MCU complex (Fig 1A) (Foskett & Philipson, 2015; Mammucari *et al*, 2018; Nemani *et al*, 2018; Pathak & Trebak, 2018; Pallafacchina *et al*, 2021). Given the functional importance of this complex, the relevance of MCU in cancers such as breast and hepatocellular carcinoma was investigated in the past (summarized in Vultur *et al*, 2018). These studies identified several important cancer-relevant mechanisms controlled by the MCU complex. However, they also presented opposing results, thus indicating that the role of MCU in tumor biology is far more complex and demands additional investigation.

Calcium signals are also important determinants of melanoma cell pathobiology (Stanisz *et al*, 2012, 2014, 2016; Hooper *et al*, 2015; Barceló *et al*, 2020). However, the role of the MCU complex and thereby mitochondrial Ca^{2+} ($_{mito}Ca^{2+}$) homeostasis in melanoma remains elusive.

Using an array of experimental techniques, proteomic and microarray screens, and bioinformatic approaches, we examined the role of the MCU_A in a panel of genetically diverse melanoma cells, in melanoma xenografts and in melanoma patient samples and datasets. Our findings demonstrate that MCU_A impacts aggressive melanoma cell behavior. Furthermore, we identify redox and metabolic signals as essential links between mitochondrial Ca^{2+} and melanoma cell aggressiveness. We also show that MCU_A controls melanoma cell sensitivity to immunotherapies and to inducers of ferroptotic cell death. Collectively, we propose that MCU_A can be utilized as a prognostic biomarker as well as a therapeutic target in treating melanoma.

Results

MCU_A expression determines melanoma patient survival

To evaluate the clinical relevance of the MCU complex, and thus mitochondrial Ca^{2+} homeostasis in melanoma, we performed bioinformatic processing of publicly available melanoma patient datasets (TCGA). To this end, we examined MCU_A expression and classified melanoma patients as high and low expressers. Using Kaplan–Meier analyses, we found that melanoma patients with lower MCU_A expression have a strongly reduced survival expectancy (Fig 1B). Further analyses suggested that this MCU_A association with patient survival is more pronounced in patients with mutations in the BRAF kinase, which is the most commonly altered molecule in cutaneous melanoma patients (Fig 1C). The association of MCU_A expression with patient survival was also observed in $BRAF_{WT}$ patients but this was less evident (Fig 1D). As BRAF kinase inhibitors such as vemurafenib or dabrafenib elevate cellular ROS production (Cesi *et al*, 2017), this finding indicated a potential role of redox signaling in the context of MCU_A expression and patient survival. The important role of MCU_A expression was also evident in the early stages (I–II) and in the late stages (III–IV) of the disease (Fig EV1A and B). These findings were intriguing since an inverse correlation between MCU_A expression and patient survival (high expression = low survival) was suggested in several other cancers such as breast and liver (Li *et al*, 2020; Zheng *et al*, 2020). To understand the role of MCU_A in a broader cancer-related context, we evaluated the influence of its expression on patient survival in a panel of additional cancer types. Our results showed that in some cancers such as kidney, bladder, cervical, colorectal, stomach, and thyroid, similarly to melanoma, MCU_A expression correlates with patient survival (i.e., low expression = low survival; Figs 1E and EV1C–G). We also found that in other cancer types such as pancreas, breast, and liver, this correlation is inverse (high expression = low survival) (Figs 1F and EV1H and I) and in cancers such as lung, brain, head and neck, ovarian, uterine, prostate, and testicular, MCU_A expression did not significantly affect patient survival (Figs 1G and EV1J–O). Given the potential clinical relevance of these findings, we sought to identify factors that regulate MCU_A expression and, more importantly, parameters that link low MCU_A levels with decreased melanoma patient survival. We thus first tested whether key genetic alterations such as BRAF or NRAS play a role in this context. Grouping melanomas by BRAF or NRAS status did not indicate that driver mutations play a role in the regulation of the MCU_A expression (Fig EV1P and Q). Moreover, our analyses also showed that other parameters such as disease stage, patients' age, or gender do not influence MCU_A expression (Fig EV1R–T). Considering the possible link between BRAF kinase inhibitors and MCU_A, we examined whether resistance to these therapeutics might affect the expression of MCU components. As seen in Fig 1H and I, MCU_A expression was decreased in both BRAF kinase inhibitor-resistant cell lines (451Lu-BR3 and WM983B-BR) as compared with their control cell lines (451Lu and WM983B). Notably, also EMRE and MICU1 were decreased in the resistant lines to some extent, suggesting that $_{mito}Ca^{2+}$ is an important parameter of acquired therapeutic resistance in melanoma.

Taken all together, we conclude that the role of MCU_A in cancer is tumor-type specific, and that low MCU_A expression is associated with decreased melanoma patient survival and therapeutic resistance.

MCU_A controls mitochondrial Ca^{2+} dynamics in melanoma cells

Upregulation of MCU_A induces elevated mitochondrial Ca^{2+} uptake while its downregulation suppresses $_{mito}Ca^{2+}$ in HEK cells

 © 2022 The Authors

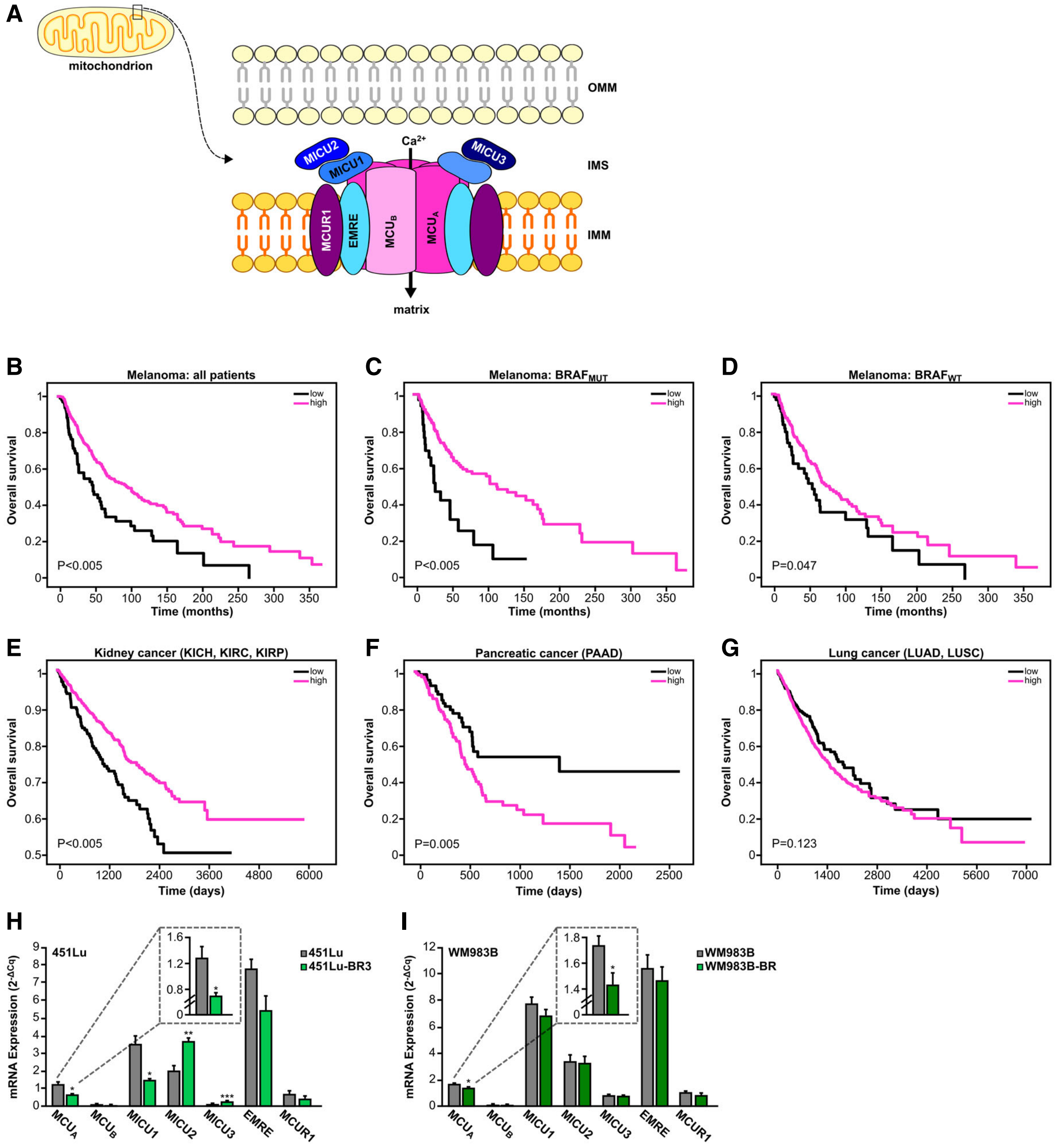

Figure 1. MCU_A controls melanoma patient survival.

A Schematic representation of the MCU complex. EMRE, essential MCU regulator; IMM, inner mitochondrial membrane; IMS, intermembrane space; MCU, mitochondrial calcium uniporter; MCUR, mitochondrial calcium uniporter regulator; MICU, mitochondrial calcium uptake; OMM, outer mitochondrial membrane.

B–D Kaplan–Meier survival plots comparing the survival probability of all melanoma patients (B), BRAF-mutated patients (C) and BRAF wild-type (WT) patients (D) with high- (magenta) and low (black) MCU_A mRNA expression.

E–G Kaplan–Meier survival plots comparing the survival probability of patients of indicated cancer types that are divided into either high- (magenta) or low (black) MCU_A mRNA expression. *P*-values were calculated using a log-rank test. KICH, kidney chromophobe; KIRC, kidney renal clear cell carcinoma; KIRP, kidney renal papillary cell carcinoma; LUAD, lung adenocarcinoma; LUSC, lung squamous cell carcinoma; PAAD, pancreatic adenocarcinoma.

H, I mRNA expression of MCU complex (MCU_A, MCU_B, MICU1, MICU2, MICU3, EMRE, and MCUR1) in 451Lu (H) and WM983B (I) melanoma cells with and without resistance to BRAF inhibitors ("BR"), normalized to housekeeping gene TBP, quantified by RT–qPCR ($n \geq 3$ biological replicates). Data are presented as mean ± SEM. Statistical significance was assessed using unpaired, two-tailed Student's *t*-test, *$P < 0.05$; **$P < 0.01$; ***$P < 0.005$; no asterisk means no statistical significance ($P > 0.05$).

(Petrungaro *et al*, 2015). To test whether this regulation applies to melanoma cells, we quantified $_{mito}Ca^{2+}$ using the 4mt-D_3cpV FRET-based biosensor (Fig 2A; Zhang *et al*, 2019). To this end, we generated two "stable" MCU_A knockdown (KD) cell lines using lentiviral transduction. Indeed, MCU_A downregulation caused a decrease of the resting $_{mito}Ca^{2+}$ as well as the

© 2022 The Authors

thapsigargin-induced mitochondrial Ca^{2+} uptake in both 1205Lu and WM3734 cell lines (Fig 2B–G). The KD efficiency was confirmed by RT–qPCR and Western blot (WB) analyses and showed an inhibition of around 70% at the mRNA level and around 95% at the protein level (Fig EV2A–D). siRNA-based silencing of MCU_A in WM3734 cells also decreased $_{mito}Ca^{2+}$ (Fig 2H), while transient overexpression caused elevation of the resting $_{mito}Ca^{2+}$ concentration (Fig 2I). The efficiency of the siRNA transfection was again confirmed by RT–qPCR and WB analyses (Fig EV2E–H). Importantly, downregulation of MCU_A did not significantly affect the expression levels of other MCU complex components (Fig EV2I–L).

To examine the contribution of MCU_A on $_{mito}Ca^{2+}$ following physiological stimulation, we first tested the effect of different stimuli on the store-operated Ca^{2+} entry (SOCE) in melanoma cells. As depicted in Fig 2J, ATP, histamine, and noradrenaline induced increase in SOCE. Because the effect of ATP was the strongest, we next evaluated its effect on $_{mito}Ca^{2+}$ in both 1205Lu and WM3734 cells. Our findings demonstrate that physiological stimulation-induced $_{mito}Ca^{2+}$ uptake is also strongly suppressed in the MCU_{A_KD} cells (Fig 2K–P).

Given that MCU_A expression is decreased in the BRAF kinase inhibitor-resistant cells, we next measured $_{mito}Ca^{2+}$ in the resistant (451Lu-BR3 and WM983B-BR) and the control (451Lu and

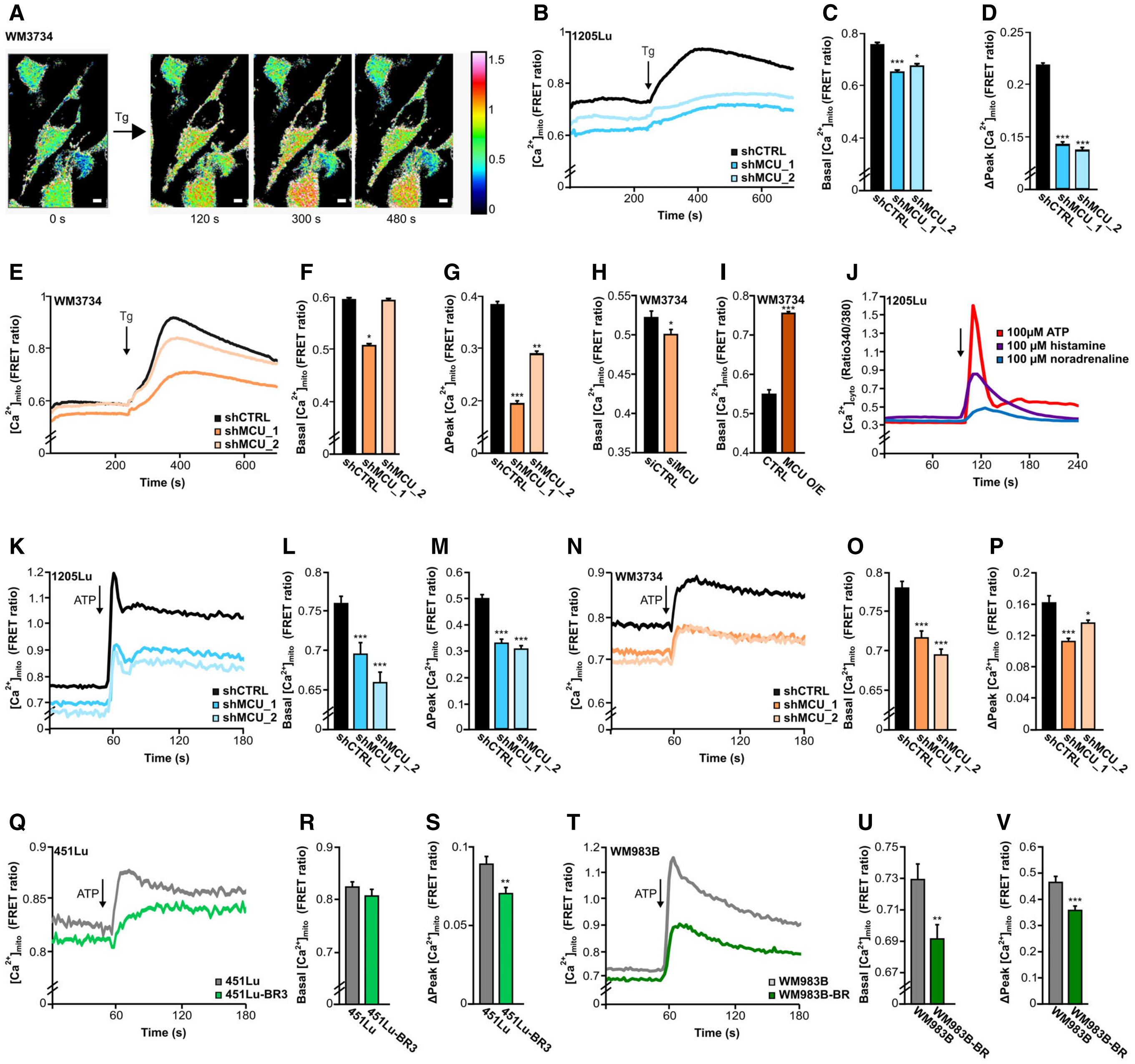

Figure 2.

 © 2022 The Authors

Figure 2. MCU_A controls mitochondrial calcium dynamics in melanoma cells.

A Representative images of mitochondrial Ca^{2+} measurements in WM3734 cells before and after thapsigargin (Tg; 1 μM) stimulation. Scale bar: 10 μm.

B–D Mitochondrial calcium uptake (represented as FRET ratio) in stable 1205Lu shCTRL (black; n = 72 cells from nine biological replicates), 1205Lu shMCU_1 (darker blue; n = 67 cells from three biological replicates) and 1205Lu shMCU_2 (lighter blue; n = 64 cells from nine biological replicates) cells upon stimulation with thapsigargin (Tg; 1 μM). (C, D) Quantification of basal levels (C) and mitochondrial Ca^{2+} uptake (Δpeak) (D) in 1205Lu stable lines with or without MCU_{A_KD}.

E–G Mitochondrial Ca^{2+} uptake (represented as FRET ratio) in stable WM3734 shCTRL (black; n = 143 cells from nine biological replicates), WM3734 shMCU_1 (darker orange; n = 156 cells from nine biological replicates) and WM3734 shMCU_2 (lighter orange; n = 135 cells from nine biological replicates) cells upon stimulation with thapsigargin (Tg; 1 μM). (F, G) Quantification of basal levels (F) mitochondrial Ca^{2+} influx (Δpeak) (G) in WM3734 stable lines with or without MCU_{A_KD}.

H Basal mitochondrial Ca^{2+} levels in WM3734 after transient $siMCU_{A\_KD}$ (siCTRL: n = 58 cells from three biological replicates; siMCU: n = 68 cells from three biological replicates).

I Basal mitochondrial Ca^{2+} levels in overexpressing (O/E) MCU WM3734 cells (CTRL: n = 52 cells from nine biological replicates; MCU O/E: n = 66 cells from nine biological replicates).

J Cytosolic Fura-2 AM-based Ca^{2+} measurements in 1205Lu cells upon physiological stimulation with ATP (100 μM), histamine (100 μM) and noradrenaline (100 μM) ($n \geq 50$ cells from three biological replicates).

K–M Mitochondrial calcium uptake (represented as FRET ratio) in stable 1205Lu shCTRL (black; n = 168 cells from nine biological replicates), 1205Lu shMCU_1 (darker blue; n = 91 from eight biological replicates) and 1205Lu shMCU_2 (lighter blue; n = 104 from eight biological replicates) cells upon physiological stimulation with ATP (100 μM). (L-M) Quantification of basal levels (L) and Ca^{2+} uptake (Δpeak) (M).

N–P Mitochondrial Ca^{2+} uptake (represented as FRET ratio) in stable WM3734 shCTRL (black; n = 147 cells from 16 biological replicates), WM3734 shMCU_1 (darker orange; n = 175 from 17 biological replicates) and WM3734 shMCU_2 (lighter orange; n = 140 from 17 biological replicates) cells upon physiological stimulation with ATP (100 μM). (O, P) Quantification of basal levels (O) and Ca^{2+} uptake (Δpeak) (P).

Q–S Mitochondrial calcium uptake (represented as FRET ratio) in 451Lu (gray; n = 157 cells from 11 biological replicates) and BRAF inhibitor-resistant 451Lu (451Lu-BR3; green; n = 164 cells from 12 biological replicates) upon physiological stimulation with ATP (100 μM). (R, S) Quantification of basal levels (R) and Ca^{2+} uptake (Δpeak) (S).

T–V Mitochondrial calcium uptake (represented as FRET ratio) in WM983B (gray; n = 151 cells from 14 biological replicates) and BRAF inhibitor-resistant WM983B (WM983B-BR; green; n = 133 cells from 11 biological replicates) upon physiological stimulation with ATP (100 μM). (U, V) Quantification of basal levels (U) and Ca^{2+} uptake (Δpeak) (V).

Data information: Data in (B–I) were obtained by imaging single cells in Ringer's buffer containing 1 mM Ca^{2+}, data in (J–V) were obtained in Ringer's buffer containing 0.5 mM Ca^{2+} and are presented as mean ± SEM. Statistical significance was determined using unpaired, two-tailed Student's t-test (shMCU, siMCU, O/E or BR cells were compared to their respective control), *P < 0.05; **P < 0.01; ***P < 0.005; no asterisk means no statistical significance (P > 0.05).

WM983B) melanoma cells. Our results showed that ATP-induced $_{mito}Ca^{2+}$ uptake is decreased in the resistant lines (Fig 2Q–V). These data thus strengthen the theory that low $_{mito}Ca^{2+}$ promotes melanoma aggressiveness.

In immune cells, mitochondrial Ca^{2+} uptake and thus MCU control SOCE by buffering Ca^{2+} and by preventing premature closure of the Orai Ca^{2+} channels (Hoth *et al*, 1997; Samanta *et al*, 2014). However, in other cell types, this mitochondria-controlled SOCE regulation was not observed. Because STIM-gated Orai channels affect melanoma cell biology (Stanisz *et al*, 2016), we measured SOCE in WM3734 MCU_{A_KD} cells and found no overt differences between the control and the MCU_{A_KD} cells. These results suggest that STIM-gated Orai channels are not involved in the MCU_A-controlled melanoma cell phenotype (Fig EV2M and N).

In summary, we confirm that MCU_A and thereby the MCU complex are central regulators of the mitochondrial Ca^{2+} homeostasis in melanoma cells. Moreover, we demonstrated that $_{mito}Ca^{2+}$ is activated by physiological stimuli and is decreased in BRAF kinase inhibitor-resistant cells.

MCU_A controls melanoma cell growth and invasion *in vitro*

Given that MCU_A expression determines $_{mito}Ca^{2+}$, melanoma patient survival and therapeutic resistance, we explored its effect on cell growth and invasive potential. To this end, we determined cell proliferation of the WM3734 MCU_{A_KD} and 1205Lu MCU_{A_KD} cells. After 24 h, we recorded a mild increase in cell growth in the MCU_{A_KD} cells. However, after 72 h, three out of four MCU_{A_KD} cell lines displayed significantly decreased cell proliferation (Fig 3A and B). To test the invasive potential of the MCU_{A_KD} cells, we performed transwell migration assays and observed a robust increase in transwell migration of 1205Lu MCU_{A_KD} cell lines (Fig 3C and D). As many cellular traits are affected by cell culture conditions and the microenvironment, especially when comparing 2D and 3D cellular cultures (Pampaloni *et al*, 2007; Kapałczyńska *et al*, 2018), we next examined the role of MCU_A in 3D collagen-embedded melanoma spheroids. As shown in Fig 3E–H, the spheroid size was reduced in all MCU_{A_KD} cell lines. Importantly, quantification of melanoma cell collagen invasion indicated that following a longer period (10 days), MCU_{A_KD} promotes melanoma cell invasive properties (Fig 3I). Next, we examined migration and invasion potentials of the BRAF kinase inhibitor-resistant cell lines and found that in both resistant lines, the transwell migration and spheroid invasion were elevated as compared with the control lines (Fig 3J–N).

Collectively, our findings suggested that lowered MCU_A levels cause decreased melanoma cell growth but augmented migration and invasion.

MCU_A regulates melanoma growth and invasion *in vivo*

The importance of MCU_A *in vivo* was examined using melanoma xenografts in immunodeficient mice. Two clones of the 1205Lu MCU_{A_KD} cells and a control transduced cell line were inoculated, and the tumor volume was measured over 36 days. As seen in Fig 3O–Q, both MCU_{A_KD} clones resulted in smaller tumors. The difference in tumor volume and weight was detected 2 week after inoculation and reached 40% smaller volumes after 6 week (Fig 3P and Q). These findings confirmed the *in vitro* observations regarding the role of MCU_A in melanoma cell growth. To examine whether this conclusion also applies to the invasive behavior, we evaluated the metastatic burden in a postmortem examination of the mouse lungs. We found that the number of metastatic lesions per mouse, the

© 2022 The Authors

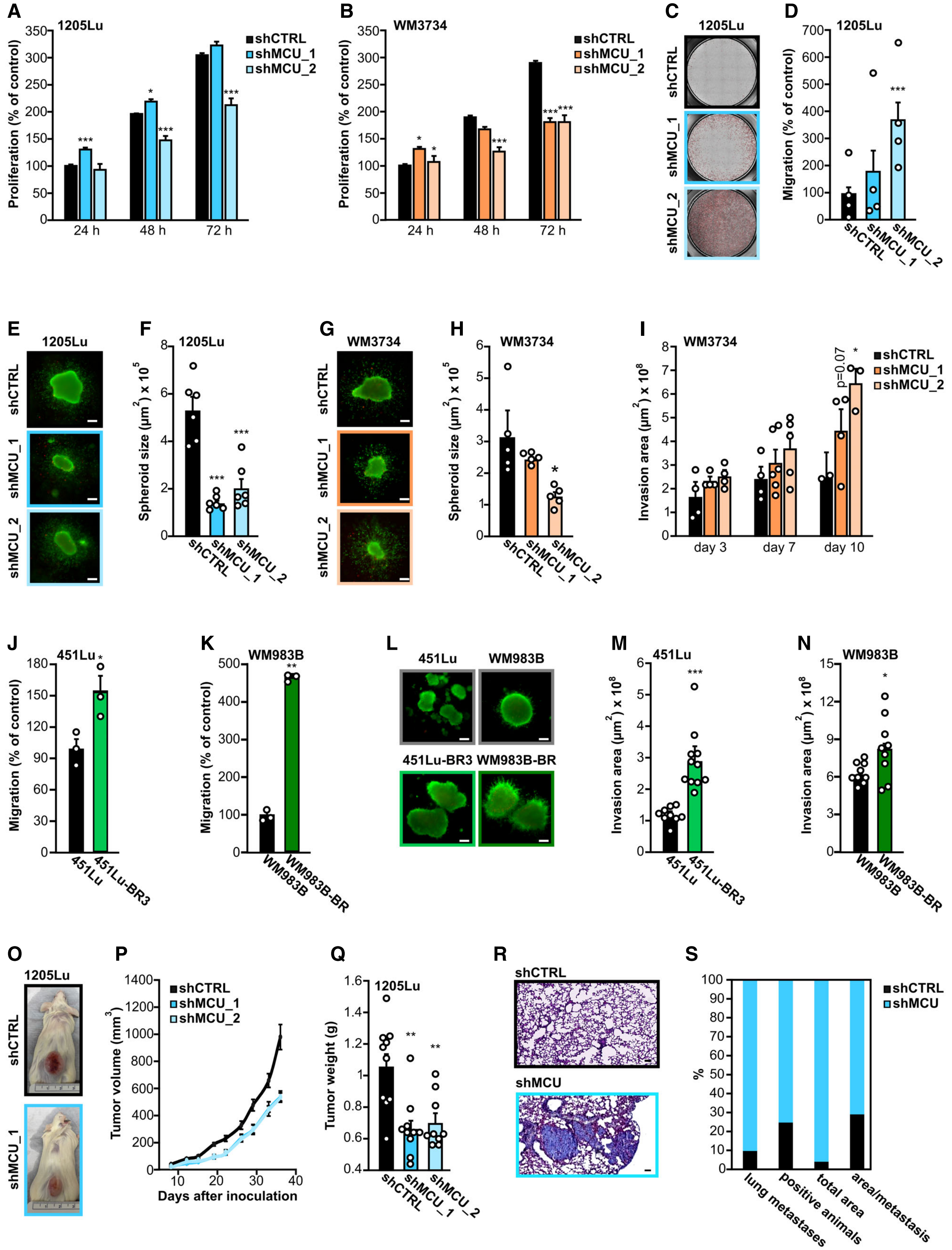
A
1205Lu
shCTRL
shMCU_1
shMCU_2
Proliferation (% of control)
24 h
48 h
72 h
B
WM3734
C
1205Lu
D
Migration (% of control)
E
F
Spheroid size (μm²) x 10⁵
G
H
I
Invasion area (μm²) x 10⁸
day 3
day 7
day 10
p=0.07
J
451Lu
451Lu-BR3
K
WM983B
WM983B-BR
L
M
N
O
P
Tumor volume (mm³)
Days after inoculation
Q
Tumor weight (g)
R
shMCU
S
%
lung metastases
positive animals
total area
area/metastasis

Figure 3.

Figure 3. MCU_A knockdown suppresses tumor growth and promotes invasion *in vitro* and *in vivo*.

A, B Proliferation of 1205Lu (A) and WM3734 (B) stable MCU_{A_KD} lines over 72 h, shown as percent of the respective control (shCTRL at 24 h) ($n \geq 7$ biological replicates/condition/day).
C Representative images of the migrated stained 1205Lu shCTRL (black frame), shMCU_1 (darker blue frame) and shMCU_2 (lighter blue frame) cells on the lower side of the insert.
D Quantification of the transwell migration in 1205Lu stable MCU_{A_KD} lines, based on the number of stained cells ($n = 4$ biological replicates/condition, shown also by individual data points).
E Representative images of 1205Lu shCTRL (black frame), shMCU_1 (darker blue frame) and shMCU_2 (lighter blue frame) melanoma spheroids after 72-h invasion in collagen. Live cells are shown in green. Scale bar: 100 μm.
F Quantification of 1205Lu stable MCU_{A_KD} spheroid core size ($n = 6$ biological replicates/condition, shown also by individual data points).
G Representative images of WM3734 shCTRL (black frame), shMCU_1 (darker orange frame) and shMCU_2 (lighter orange frame) melanoma spheroids. Live cells are shown in green. Scale bar: 100 μm.
H Quantification of WM3734 stable MCU_{A_KD} spheroid core size ($n = 5$ biological replicates/condition, shown also by individual data points).
I Invasion potential of WM3734 shCTRL (black), shMCU_1 (darker orange) and shMCU_2 (lighter orange) over a period of 10 days ($n \leq 4$ biological replicates/condition/day, shown also by individual data points).
J, K Migration potential of 451Lu and 451Lu BRAF inhibitor-resistant (451Lu-BR3) (J) and WM983B and WM983B BRAF inhibitor-resistant (WM983B-BR) cells (K) over 24 h ($n = 3$ biological replicates/condition, shown also by individual data points).
L Representative images of 451Lu wild-type (gray frame), 451Lu-BR3 (lighter green frame), WM983B wild-type (gray frame) and WM983B-BR (darker green frame) melanoma spheroids after 72-h invasion in collagen. Live cells are shown in green. Scale bar: 100 μm.
M, N Quantification of 451Lu versus 451Lu-BR3 (M) and WM983B versus WM983B-BR (N) spheroid core size ($n \geq 9$ biological replicates/condition, shown also by individual data points).
O Representative images of mice in the shCTRL group and the shMCU_1 group after 36 days of tumor growth ($n = 10$ mice/group).
P Tumor volume growth over 36 days postinoculation.
Q Average weight of the tumors at the end point (36 days) upon removal (individual values shown as points).
R Exemplary image of 1205Lu shCTRL (black frame) and shMCU (blue frame) metastases in mouse lung tissue sections; paraffin sections were made from the removed lungs; H&E staining was carried out and the sections were scanned using an Axio Scan.Z1 microscope. Scale bar: 100 μm.
S Quantitative evaluation of the incidence (shown in percent) of lung metastases, total amount of positive animals identified with metastases, total area of all metastases in a group, and the average area per metastasis in shCTRL group (black) and shMCU group (blue).

Data information: Data are presented as mean ± SEM. Statistical significance was determined using unpaired, two-tailed Student's *t*-test (shMCU cells were compared with their respective control, shCTRL), $^{*}P < 0.05$; $^{**}P < 0.01$; $^{***}P < 0.005$; no asterisk means no statistical significance ($P > 0.05$).

number of metastasis-positive mice, the total metastatic area, and the area/metastatic lesion ratio were higher in the mice inoculated with 1205Lu MCU_{A_KD} cells compared with the control cell line (Fig 3R and S). The MCU_{A_KD} efficiency was maintained *in vivo* over the 6 week after inoculation, as confirmed by WB analyses of tumor lysates (Fig EV3A and B).

Our *in vivo* experiments suggest that MCU_A and thereby the mitochondrial Ca^{2+} homeostasis play an important role as regulators of melanoma pathobiology.

Proteomic analyses identify proteins and signaling pathways regulated by MCU_A

To identify molecular determinants and signaling mechanisms that are influenced by the abundance and activity of MCU_A, we performed proteomic and microarray analyses of MCU_{A_KD} cells and the corresponding control cell lines. The volcano plot depicting the data from proteome analyses indicates that the abundance of a number of proteins is significantly altered (Fig 4A, red and green, see also Dataset EV1) following MCU_A knockdown in the WM3734 cells. A "proteomap"-based pathway analysis indicated a high contribution of cellular metabolism and bioenergetics, protein folding, sorting and translation, as well as vesicular transport and cell adhesion, in the MCU_{A_KD}-regulated proteins (Fig 4B and C). Similar findings were observed when we analyzed the proteomic data obtained from control and MCU_{A_KD} 1205Lu cells (Fig EV3C–E, see also Dataset EV2). The subsequent KEGG-based pathway analysis depicted a robust melanoma cell metabolic reprograming as a number of metabolic pathways such as the pentose phosphate pathway, the TCA cycle, and the glutathione metabolism were significantly enriched following MCU_A downregulation (Figs 4D and EV3F). In addition, the evaluation of the affected biological processes and molecular functions identified response and binding to ascorbic acid, dioxygenase activity as well as NADPH regeneration and NAD binding as processes controlled by MCU_A. These data thus suggested that in the context of the MCU_{A_KD}-induced metabolic reprograming, redox regulation plays a central role in melanoma cell phenotype control (Fig 4E and F). We also assessed the proteomic data according to the "Hallmarks of Cancer" classification and calculated the fraction of MCU_A-controlled proteins into each hallmark as defined by Hanahan & Weinberg (2011) (Figs 4G and EV3G). This analysis identified MCU_A-controlled proteins involved in several cancer hallmarks such as reprograming energy metabolism, activating invasion and metastasis and resisting cell death and further strengthened the hypothesis that MCU_A is a critical regulator of melanoma cell pathobiology.

To further decipher the role of MCU_A in melanoma, we performed reverse phase protein array (RPPA), an antibody-based screening tool that allows evaluation of abundance and/or activation state of cancer-relevant proteins (Lu *et al*, 2016). As shown in the cluster map (Fig 4H, see also Dataset EV3), MCU_{A_KD} significantly affected the abundance and/or activity of several proteins. The proteomaps and KEGG-based pathway analyses again suggested a significant contribution of MCU_A in cell metabolism but also in transcription, signaling, protein quality control and cell growth, death, and invasion (Fig 4I and J). The subsequent pathway and functional analyses (Fig 4K–M) indicated that a number of metabolic and signaling pathways, T cell-relevant biological processes, kinase and phosphatase activity and cell death mechanisms are under control of or are influenced by the mitochondrial Ca^{2+} homeostasis. The Hallmarks of Cancer analyses indicated a high occurrence of the identified "hits" and confirmed the proteomic findings

© 2022 The Authors

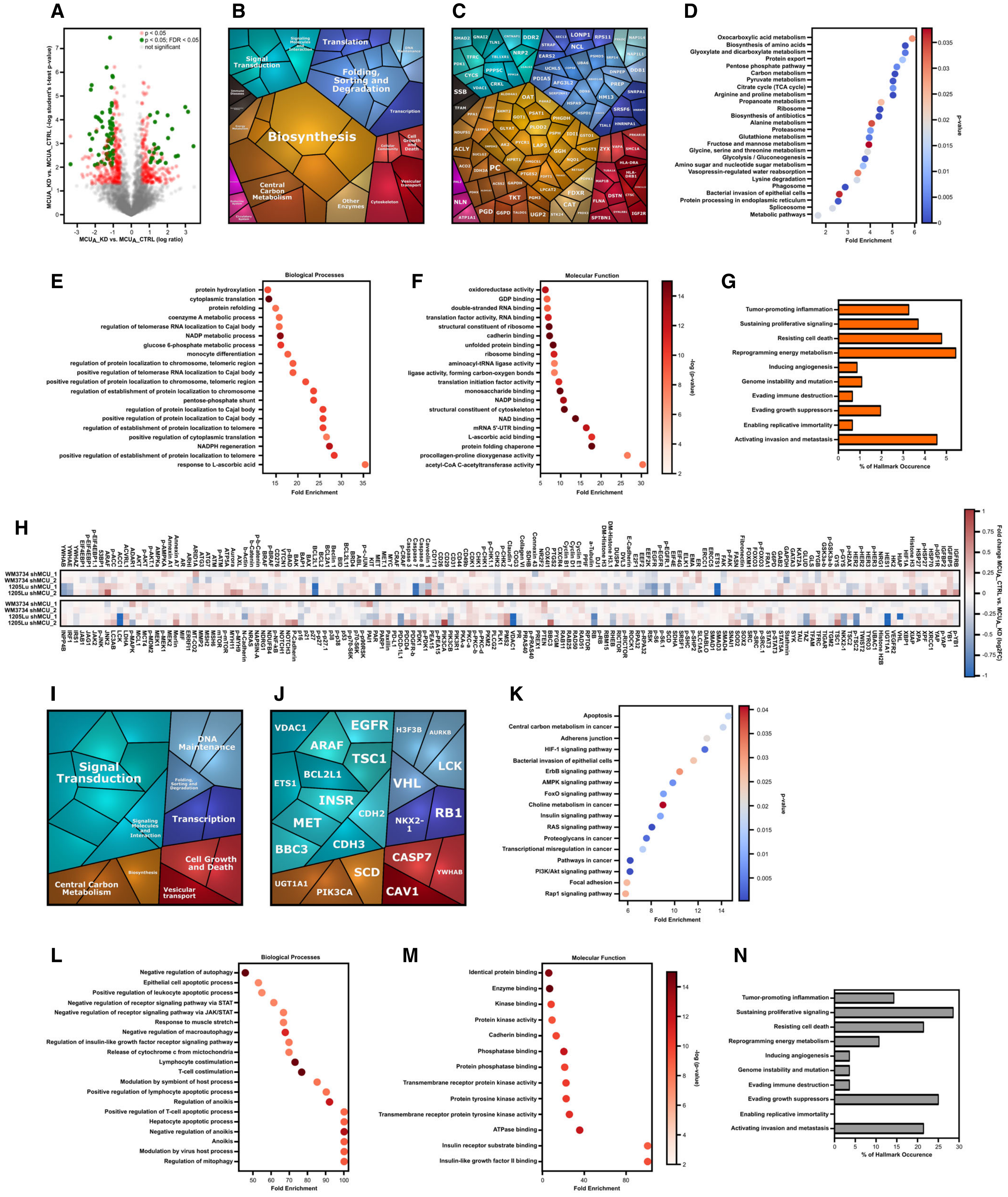

Figure 4.

 © 2022 The Authors

◀ **Figure 4. MCU_A controls metabolic signaling in melanoma cells.**

A Volcano plot of proteomics data for WM3734 MCU_{A_KD} versus shCTRL. Protein hits with significant *P*-values ($P < 0.05$) are marked in red. Protein hits with both significant *P*-value and FDR (false discovery rate) ($P < 0.05$; FDR < 0.05) are marked in green.
B, C Proteomap analyses in WM3734 MCU_{A_KD} cells, based on data shown in (A) (red dots).
D KEGG-based analysis of cellular components and processes based on protein hits that show differential expression between in WM3734 MCU_{A_KD} and shCTRL cells, revealed via proteomics.
E, F Enrichment analyses of GO terms and pathways in WM3734 MCU_{A_KD} cells, based on proteomic analysis. GO analyses were performed according to two GO categories: biological processes (E) and molecular function (F).
G Cancer hallmark-based enrichment analysis based on proteomics data shown in (A). Percentage of hits in the specific gene set is displayed.
H Heatmap encompassing RPPA values of proteins detected in WM3734 and 1205Lu MCU_{A_KD} (shMCU_1 and shMCU_2) cells.
I, J Proteomap-based pathway analyses in WM3734 and 1205Lu MCU_{A_KD} cells, based on RPPA data shown in (H).
K KEGG-based analysis of cellular components and processes involved in WM3734 and 1205Lu MCU_{A_KD} cell regulation.
L, M Enrichment analyses of GO (gene ontology) terms and pathways in WM3734 and 1205Lu MCU_{A_KD} cells, based on RPPA data. GO analyses were performed according to two GO categories: biological processes (L) and molecular function (M).
N Hallmark of cancer-based enrichment analysis in WM3734 and 1205Lu MCU_{A_KD} cells, based on RPPA data. Percentage of hits in the specific gene set is displayed. Gene sets (10 hallmarks): http://bio-bigdata.hrbmu.edu.cn/CHG/index.html.

that MCU_A is involved in hallmarks such as activating invasion and metastasis, sustaining proliferation, and reprograming energy metabolism (Fig 4N).

Redox signals underlie the MCU_A-driven melanoma cell phenotype

Our proteomic and RPPA screens indicated that MCU_A and thereby ${}_{mito}Ca^{2+}$ play a distinctive role in several cancer-relevant signaling pathways and cellular functions. Based on these findings and given that ${}_{mito}Ca^{2+}$ is an important regulator of mitochondrial metabolic output and redox signaling (Hoffman *et al*, 2014; Tosatto *et al*, 2016; Joseph *et al*, 2019; Zhang *et al*, 2019; Booth *et al*, 2021), we next examined the role of MCU_A in mitochondrial redox homeostasis, that is, H_2O_2 dynamics. Fluorescence live-cell microscopy using H_2O_2 biosensors (HyPer and mito-HyPer) demonstrated that the cytosolic and mitochondrial H_2O_2 levels in both 1205Lu and WM3734 MCU_{A_KD} cells were significantly decreased (Fig 5A–F). Moreover, transient silencing of MCU_A caused decreased cytosolic H_2O_2 levels (Fig 5G). On the contrary, overexpression of MCU_A, which led to elevated ${}_{mito}Ca^{2+}$, induced an increase in the cytosolic H_2O_2 concentration (Fig 5H). Measurements of the mitochondrial glutathione redox potential with another mitochondria-targeted biosensor (${}_{mito}$Grx1-roGFP2) demonstrated a more reducing redox state in the mitochondrial matrix of all four MCU_{A_KD} cell lines (Fig 5I–K). Measurements of mitochondrial H_2O_2 in the BRAF kinase inhibitor-resistant cells showed decreased mitochondrial H_2O_2 levels relative to the control cells (Fig 5L and M). Of note, the control SypHer-based measurements indicated that mitochondrial and intracellular pH was not altered in the MCU_{A_KD} cells (Fig EV4A–F). Collectively, the redox measurements strengthen the hypothesis that MCU_A is an important regulator of the mitochondrial and cellular redox homeostasis.

Redox signals have been identified as regulators of melanoma invasive potential and metastatic spread *in vitro* and *in vivo* (Le Gal *et al*, 2015; Piskounova *et al*, 2015); hence, we hypothesized that MCU_A-controlled H_2O_2 production, that is, redox regulation, is a molecular mechanism that links decreased ${}_{mito}Ca^{2+}$ and increased melanoma cell invasiveness. We tested this theory by measuring transwell migration of melanoma cells treated with antioxidants such as N-acety-cystein (NAC) and prooxidants such as buthionine sulphoximine (BSO) and H_2O_2. Our results confirmed that antioxidants promote melanoma cell migration and also showed that this increase can be reversed by the addition of oxidants (Fig 5 N and O). Moreover, we found that the MCU_{A_KD}-induced increase in melanoma cell migration is diminished when cells are pretreated with H_2O_2 (Fig 5P). Of note, the BSO, NAC, and H_2O_2 concentrations used for these experiments did not affect the viability of 1205Lu and WM3734 melanoma cells following 4 h of stimulation (Fig EV4G–I) but NAC and the mitochondria-targeted antioxidant mitoTEMPO suppressed the mitochondrial H_2O_2 production (Fig EV4J and K). These findings thus corroborated the hypothesis that MCU_A-controlled redox signals are involved in regulating melanoma aggressive behavior.

MCU_A controls mitochondrial ATP production

To evaluate the impact of MCU_A on mitochondrial function, we determined the inner mitochondria membrane (IMM) potential. Our single-cell-based fluorescence measurements showed that in the MCU_{A_KD} melanoma cells, the IMM potential is significantly reduced (Fig 6A–D). These findings suggested that mitochondrial ATP production might also be affected by MCU_{A_KD}. We thus measured ATP levels using a fluorescent dye as well as a genetically encoded mitochondrial ATP sensor, mito-A_{TEAM}. Both measurements demonstrated that mitochondrial ATP levels are diminished in the 1205Lu- and the WM3734 MCU_{A_KD} cells (Fig 6E–I).

Mitochondrial Ca^{2+} induces dehydrogenase activity in the matrix and thus fuels ATP synthesis. Accordingly, the decreased ATP levels following MCU_{A_KD} were not an unexpected finding; however, MCU_A could have additional effects that may influence mitochondrial bioenergetics. For example, MCU_A could modulate mitochondrial dynamics and structure. We explored this possibility by performing confocal- and electron microscopy. As seen in Fig 6J–O, neither the overall morphology of the mitochondrial network, nor the parameters such as mitochondrial diameters and the distances between cristae were altered in the MCU_{A_KD} melanoma cells, indicating that the regulation of melanoma biology by MCU_A is mostly governed by the ${}_{mito}Ca^{2+}$-ATP-redox axis.

MCU_A affects melanoma cell therapeutic sensitivity

Current treatments of advanced melanoma encompass targeted- and immunotherapies (Pasquali *et al*, 2018; Schadendorf *et al*, 2018;

Jenkins & Fisher, 2020). Given the effects of MCU_A expression and its effects on melanoma cell growth, invasion, and patient survival, we assessed the involvement of MCU_A in melanoma cell therapeutic sensitivity. To this end, we first identified transcripts that correlate with those of MCU_A in the TCGA-derived melanoma patient dataset (Fig 1). The proteomaps (Fig 7A and B) showed that in addition to metabolism, genetic information processing and signal transduction are processes whose expression correlated with MCU_A and are also involved in regulating immune responses (Dataset EV4). The subsequent KEGG-based pathway analysis further strengthened these findings (Fig 7C). As shown, the correlating genes were involved in a number of immune cell-relevant and metabolic pathways. This was further corroborated by the GO term-based evaluation of biological processes and molecular functions which demonstrated a robust enrichment of transcripts involved in antigen presentation, MHC activity, cytokine secretion, and other functions that can determine melanoma cell interaction with T- and NK cells (Fig 7D and E). These findings thus suggested that MCU_A is an important determinant of melanoma cell therapeutic responses. To test this hypothesis, we first treated MCU_{A_KD} cells with clinically relevant small molecule inhibitors, that is, trametinib (MEK inhibitor) and vemurafenib (BRAF inhibitor). As expected, both drugs reduced melanoma cell viability in a concentration-dependent manner (Figs 7F and G, and EV5A and B). However, we did not observe differences between the parental and the MCU_{A_KD} cells. To test their sensitivity to immunotherapies, we used primary human natural killer (NK) cells from healthy human donors and determined NK cell-mediated melanoma cell killing (*NKmK*; Cappello *et al*, 2021). Notably, *NKmK* of MCU_{A_KD} cells was diminished compared with the control cells (Fig 7H and I). Next, we treated the cells with the antineoplastic chemotherapeutic agent mitoxantrone, which is not used to treat melanoma but was recently reported as a potent inhibitor of MCU (Arduino *et al*, 2017). To this end, we examined its effect on melanoma cell viability and found that MCU_{A_KD} cells are less sensitive to this inhibitor (Fig 7J). Based on our findings regarding the role of MCU_A on the redox status of melanoma cells and the fact that ferroptosis is a cell death mechanism controlled by oxidative stress (Jiang *et al*, 2021), we treated both control and MCU_{A_KD} cells with two ferroptosis inducers (FINs). As demonstrated, MCU_{A_KD} cells were more resistant to BSO, an agent that lowers glutathione levels

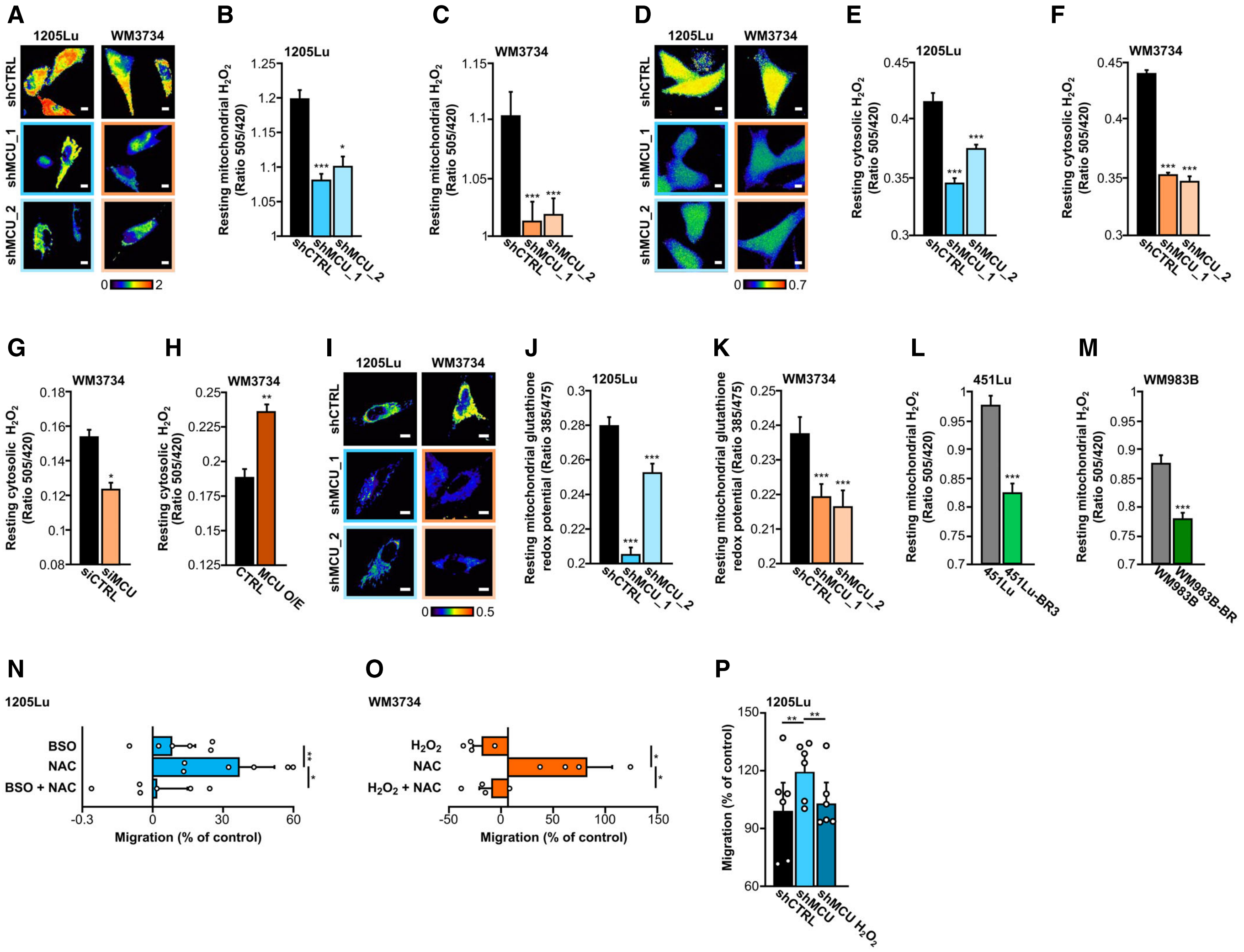

Figure 5.

 © 2022 The Authors

Figure 5. MCU_A controls redox signaling in melanoma cells.

A–C Mitochondrial hydrogen peroxide (H_2O_2) measurement in 1205Lu and WM3734 with and without stable MCU_{A_KD} using mito-HyPer. Exemplary ratiometric images (F505 nm/F420 nm) are shown for all conditions (A). Scale bar: 10 μm. Quantification of mito-HyPer ratio in 1205Lu (B) and WM3734 (C) under resting state (1205Lu—shCTRL: n = 217 cells from six biological replicates; shMCU_1: n = 197 cells from seven biological replicates; shMCU_2: n = 212 cells from seven biological replicates; WM3734—shCTRL: n = 104 cells from six biological replicates; shMCU_1: n = 107 cells from seven biological replicates; shMCU_2: n = 114 cells from seven biological replicates).

D–F Cytosolic hydrogen peroxide (H_2O_2) measurement in 1205Lu and WM3734 with and without stable MCU_{A_KD} using HyPer. Exemplary ratiometric images (F505 nm/F420 nm) are shown for all conditions (D). Scale bar: 10 μm. Quantification of HyPer ratio in 1205Lu (E) and WM3734 (F) under resting state (1205Lu—shCTRL: n = 232 cells from six biological replicates; shMCU_1: n = 290 cells from six biological replicates; shMCU_2: n = 290 cells from six biological replicates; WM3734—shCTRL: n = 443 cells from nine biological replicates; shMCU_1: n = 297 cells from nine biological replicates; shMCU_2: n = 233 cells from nine biological replicates).

G, H Quantification of resting cytosolic H_2O_2 levels (HyPer) in WM3734 upon siRNA-mediated MCU_{A_KD} (siCTRL: n = 58 cells from nine biological replicates; siMCU: n = 68 cells from nine biological replicates) (G) and MCU_A overexpression (O/E) (CTRL: n = 52 cells from nine biological replicates; MCU O/E: n = 66 cells from nine biological replicates) (H).

I–K Mitochondrial glutathione redox potential, measured with mito-Grx1-roGFP2 in 1205Lu and WM3734 with and without stable MCU_{A_KD}. Representative ratiometric images (F385 nm/F475 nm) are shown for all conditions (I). Scale bar: 10 μm. Quantification in 1205Lu (J) and WM3734 (K) under resting state (1205Lu—shCTRL: n = 231 cells from 10 biological replicates; shMCU_1: n = 176 cells from seven biological replicates; shMCU_2: n = 164 cells from seven biological replicates; WM3734—shCTRL: n = 127 cells from seven biological replicates; shMCU_1: n = 142 cells from seven biological replicates; shMCU_2: n = 116 cells from seven biological replicates).

L, M Resting mitochondrial hydrogen peroxide (H_2O_2; mito-HyPer) in 451Lu and BRAF inhibitor-resistant 451Lu-BR3 (L) and WM983B and BRAF inhibitor-resistant WM983B-BR (M) (451Lu: n = 227 cells from eight biological replicates; 451Lu-BR3: n = 166 cells from six biological replicates; WM983B: n = 193 cells from eight biological replicates; WM983B-BR: n = 208 cells from nine biological replicates).

N Migration (4 h) of 1205Lu wild-type cells upon 4-h pretreatment with 1 mM BSO, 200 μM NAC and 1 mM BSO + 200 μM NAC (n = 6 biological replicates/condition, shown also by individual data points).

O Migration (4 h) of WM3734 wild-type cells upon 4-h pretreatment with 100 μM H_2O_2, 200 μM NAC and 100 μM H_2O_2 + 200 μM NAC (n = 4 biological replicates/condition, shown also by individual data points).

P Migration of 1205Lu shCTRL and shMCU untreated and shMCU pretreated for 4 h with 100 μM H_2O_2 (n = 6 biological replicates/condition, shown also by individual data points).

Data information: All data are presented as mean ± SEM. (A–F) and (I–M) were measured in Ringer's buffer containing 0.5 mM Ca^{2+} and (G-H) in Ringer's buffer containing 1 mM Ca^{2+}. For (A–M), statistical significance was determined using unpaired, two-tailed Student's t-test, *$P < 0.05$; **$P < 0.01$; ***$P < 0.005$. For (N, O), statistical significance was determined using paired, one-tailed Student's t-test, *$P < 0.05$; **$P < 0.01$. For (P), statistical significance was assessed using paired, two-tailed Student's t-test, **$P < 0.01$. In all statistical analyses, KD, O/E or treated cells were compared with their respective control.

and thus induces ferroptosis (Fig 7K). This ferroptotic resistance was even more prominent when melanoma cells were treated with RSL3 (RAS-selective lethal), a drug that induces ferroptosis by inhibiting the glutathione peroxidase 4 (GPX4) (Jiang *et al*, 2021) (Figs 7L and EV5C).

In summary, the presented bioinformatic- and experimental data indicate that MCU_A regulates the immunogenicity of melanoma cells and thus their sensitivity to immune cell-based therapies. In addition, the findings that MCU_A abundance determines the sensitivity to ferroptosis established a direct link between the altered metabolism, that is, redox status and melanoma aggressive behavior.

Discussion

The importance of Ca^{2+} and redox regulation in cancer is nowadays widely accepted, and the number of genes and proteins involved in controlling molecular mechanisms that link Ca^{2+} and redox signals with cancer biology is constantly increasing (Chio & Tuveson, 2017; Monteith *et al*, 2017; Marchi *et al*, 2020). In this context, several studies suggest that the MCU complex is an important determinant of cancer biology (summarized in Vultur *et al*, 2018). However, the current knowledge regarding the role of MCU in cancer is incomplete, and the molecular mechanisms involved and controlled by the Ca^{2+}-transporting complex are not fully understood.

Our findings revealed that MCU_A and thereby ${}_{mito}Ca^{2+}$ control melanoma patient survival and melanoma cell aggressive behavior. In this context, we demonstrated that acquired resistance to BRAF kinase inhibitors causes reduced MCU_A expression and reduced mitochondrial Ca^{2+} uptake. We also found that melanoma cell phenotype switching, a concept known to control melanoma progression and therapeutic sensitivity, is of central importance in this regard (Rambow *et al*, 2019). Indeed, we observed a decreased cell growth and increased invasive properties of MCU_{A_KD} cells *in vitro* and *in vivo*. The proteomic and RPPA-based screens demonstrated that the highest fraction of proteins affected by MCU_{A_KD} are involved in controlling cellular metabolism and redox signaling, in addition to environmental- and genetic information processing. Moreover, measurements of parameters, such as ATP production, IMM potential, and mitochondrial structure, suggest that MCU_A controls mitochondrial function and bioenergetic output in melanoma cells. Given that bioenergetics, metabolism, and redox signaling play a critical role in cancer pathobiology (Diebold & Chandel, 2016; Panieri & Santoro, 2016; Hempel & Trebak, 2017; Zhang *et al*, 2019; Tasdogan *et al*, 2020), we sought to decipher the role of MCU_A in melanoma by exploring the contribution of the involved signaling mechanisms and pathways. In addition, we pursued this direction based on studies which suggested that redox signals determine the melanoma cell invasive phenotype, whereby ROS, antioxidants, and the redox state of the cellular environment, that is, the blood and the lymph system control melanoma metastatic spread and sensitivity to ferroptotic cell death (Le Gal *et al*, 2015; Piskounova *et al*, 2015; Ubellacker *et al*, 2020).

Our findings are in line with these studies but also provide novel insights and identify molecular players that are involved in the Ca^{2+}-controlled metabolic regulation of melanoma. This study also indicates that manipulations of ${}_{mito}Ca^{2+}$ as a tool to modulate redox signaling might be applicable in treating melanoma and other

© 2022 The Authors

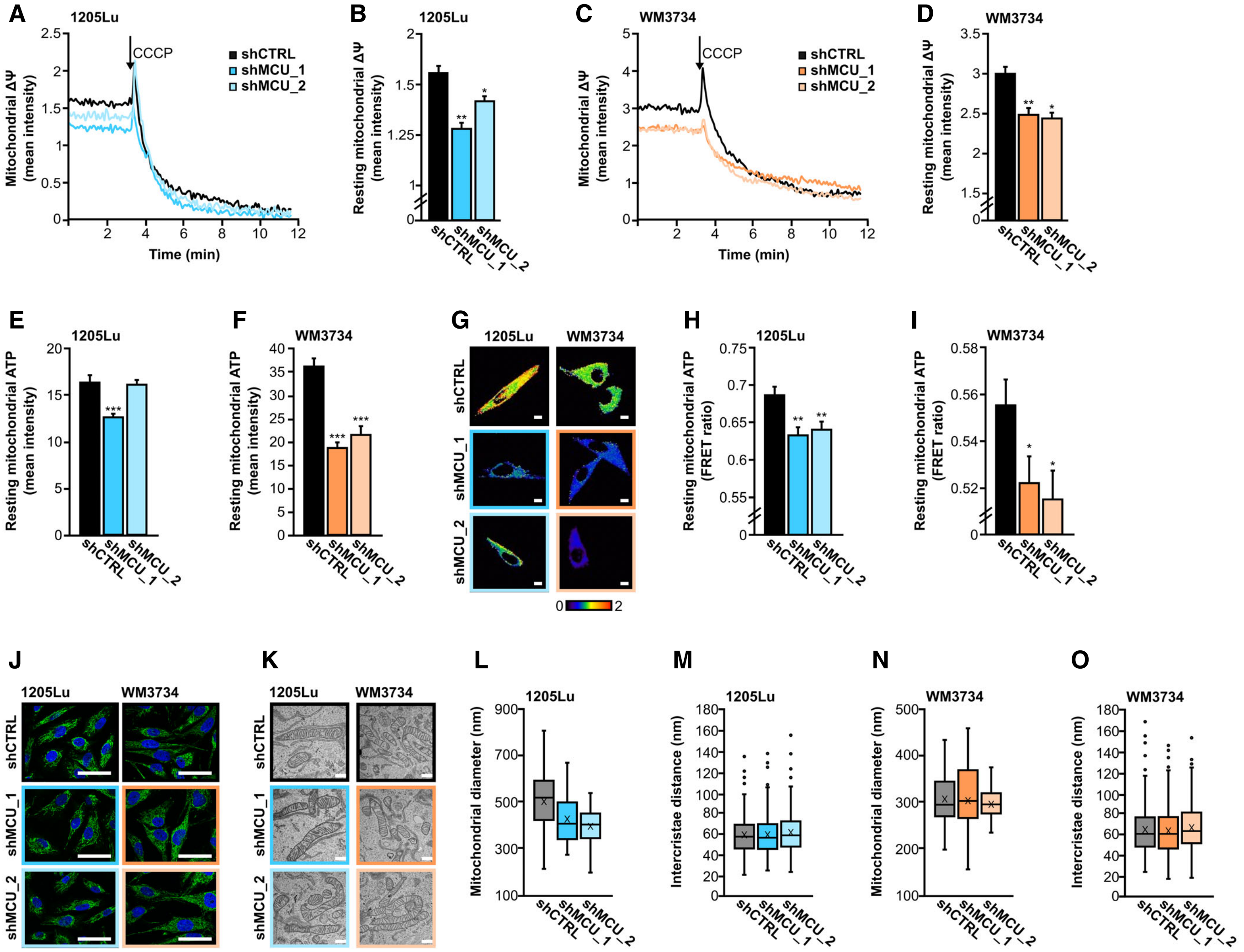

Figure 6. MCU_A controls mitochondrial membrane potential and ATP production.

A–D Resting mitochondrial membrane potential (ΔΨ), measured with TMRE in 1205Lu (A, B) and WM3734 (C, D) with and without stable MCU_{A_KD} (1205Lu—shCTRL: *n* = 81 cells from three biological replicates; shMCU_1: *n* = 81 cells from three biological replicates; shMCU_2: *n* = 156 cells from three biological replicates; WM3734—shCTRL: *n* = 108 cells from three biological replicates; shMCU_1: *n* = 73 cells from three biological replicates; shMCU_2: *n* = 110 cells from three biological replicates).

E, F Resting mitochondrial ATP levels, measured using the ATP-Red dye in 1205Lu (E) and WM3734 (F) with and without stable MCU_{A_KD} (1205Lu—shCTRL = 67 cells from at least three biological replicates; shMCU_1: *n* = 80 cells from at least three biological replicates; shMCU_2: *n* = 69 cells from at least three biological replicates; WM3734—shCTRL: *n* = 40 cells from at least three biological replicates; shMCU_1: *n* = 38 cells from at least three biological replicates; shMCU_2: *n* = 21 cells from at least three biological replicates).

G–I Mitochondrial ATP, measured using mito-ATEAM in 1205Lu and WM3734 with and without stable MCU_{A_KD}. Exemplary ratiometric images (FRET/CFP) are shown for all conditions (G). Scale bar: 10 μm. Quantification of basal levels in 1205Lu (H) and WM3734 (I) (1205Lu—shCTRL: *n* = 141 cells from 14 biological replicates; shMCU_1: *n* = 143 cells from 12 biological replicates; shMCU_2: *n* = 143 cells from 13 biological replicates; WM3734—shCTRL: *n* = 122 cells from 12 biological replicates; shMCU_1: *n* = 103 cells from 12 biological replicates; shMCU_2: *n* = 99 cells from 13 biological replicates).

J Exemplary confocal microscope images of the mitochondrial network (blue: DAPI staining of the nucleus; green: TOMM20 staining of mitochondria); scale bar: 50 μm. (K–O) Electron microscopy of mitochondria of stable MCU_{A_KD} cell lines.

K Exemplary images of stable MCU_{A_KD} cells' mitochondria. Scale bar: 500 nm.

L–O Quantification of mitochondrial diameter (L and N) and intercristae distance (M and O) of 1205Lu (L, M) and WM3734 (N, O) cells with and without stable MCU_{A_KD}, presented as boxplot. The box presents the 25%- quartile, median and 75%-quartile, the X represents the mean and the whiskers the minimum and maximum, outliers are represented as dots (mitochondrial diameter: 1205Lu—shCTRL: *n* = 47 technical replicates; shMCU_1: *n* = 44 technical replicates; shMCU_2: *n* = 40 technical replicates; WM3734—shCTRL: *n* = 35 technical replicates; shMCU_1: *n* = 52 technical replicates; shMCU_2: *n* = 38 technical replicates; intercristace distance: 1205Lu—shCTRL: *n* = 330 technical replicates; shMCU_1: *n* = 285 technical replicates; shMCU_2: *n* = 255 technical replicates; WM3734—shCTRL: *n* = 352 technical replicates; shMCU_1: *n* = 433 technical replicates; shMCU_2: *n* = 357 technical replicates).

Data information: Statistical significance was determined using unpaired, two-tailed Student's *t*-test, $*P < 0.05$; $**P < 0.01$; $***P < 0.005$; no asterisk means no statistical significance ($P > 0.05$).

© 2022 The Authors

cancers. More concretely, our results suggest that mitochondrial Ca^{2+} uptake alters the cellular redox status and causes a more proliferative but less invasive melanoma cell phenotype. Given that such growth-oriented phenotypes are in general more responsive to conventional anticancer therapies, we hypothesize that the activation of MCU will increase the therapeutic sensitivity of melanoma cells and have a positive effect on patient survival. To this end, not only activators of MCU_A can be applied but also modulators of the other MCU complex components such as MICU1-2, EMRE, and MCUR1 (Di Marco et al, 2020; De Mario et al, 2021). Inhibitors of other mitochondrial proteins responsible for Ca^{2+} transport across the IMM such as NCLX, UCPs, and Letm1 might have similar

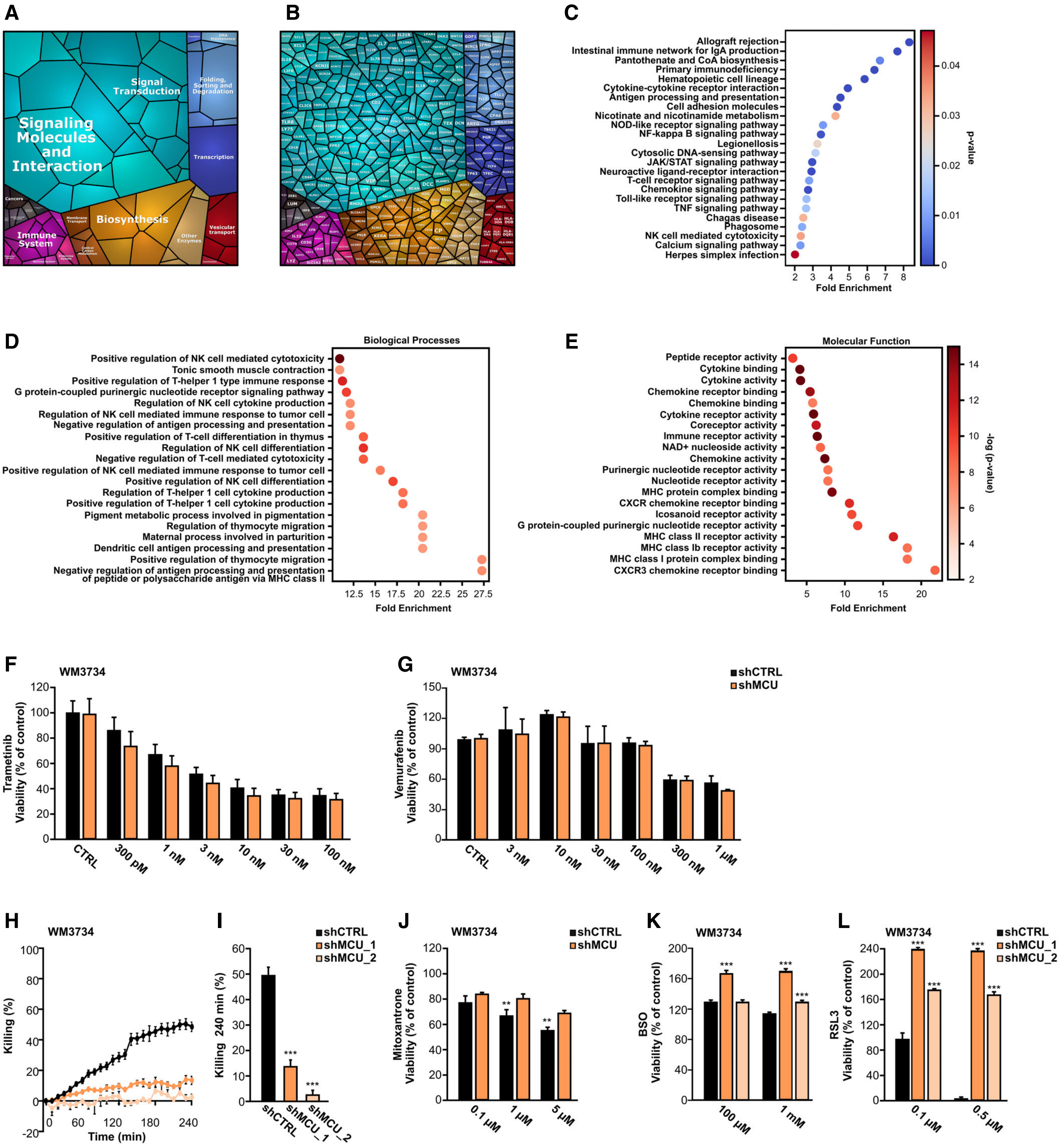

Figure 7.

Figure 7. MCU_A controls melanoma cell therapeutic sensitivity.

A, B Proteomap analyses of genes significantly correlating with MCU_A expression in the TCGA-derived melanoma patient dataset.

C KEGG-based analysis of cellular components and processes based on protein hits shown in A and B.

D, E Enrichment analyses of GO terms and pathways based on protein hits shown in A and B. GO analysis were performed according to two GO categories: biological processes (D) and molecular function (E). Significant cellular components and biological processes ($P < 0.05$) are indicated and ranked by fold enrichment.

F, G WM3734 shCTRL and MCU_{A_KD} cell viability following incubation with different concentrations of trametinib (F) and vemurafenib (G) for 72 h ($n = 3$ biological replicates).

H, I (H) Kinetics and (I) quantification of NK cell-mediated melanoma killing in WM3734 shCTRL and the two MCU_{A_KD} clones ($n = 6$ biological replicates).

J WM3734 shCTRL and shMCU cell viability upon mitoxantrone treatment. Results are depicted as percent of control (DMSO-treated cells, not shown) ($n = 3$ biological replicates).

K, L WM3734 cell viability upon incubation with different concentrations of BSO (K) and RSL3 (L) for 72 h. Data are shown as percent of control (untreated cells, not shown) ($n \leq 12$ wells from three biological replicates/condition). BSO, L-buthionine-sulfoximine; RSL, RAS-selective lethal.

Data information: Data are presented as mean ± SEM. Statistical significance was determined using unpaired, two-tailed Student's *t*-test, $^{**}P < 0.01$; $^{***}P < 0.005$; no asterisk means no statistical significance ($P > 0.05$).

therapeutic effects (Bondarenko *et al*, 2015; Lin & Stathopulos, 2019; Pathak *et al*, 2020; Madreiter-Sokolowski *et al*, 2021).

Our therapeutic assays demonstrate that MCU_A affects melanoma cell sensitivity to immunotherapies and ferroptosis. To this end, the bioinformatic analyses of melanoma patient datasets identified genes whose expression correlates with the one of MCU_A. Notably, most of these genes are involved in shaping the immunogenicity of melanoma tumors. These molecular determinants, together with $MCU_{A,}$ have thus the potential to be explored as biomarkers and predictors of patient therapeutic responses. In addition, the identification of the molecular determinants underlying the robust resistance toward ferroptosis inducers in cells lacking MCU_A might provide novel therapeutic targets for treating metastatic melanoma.

Our study demonstrates that in melanoma, MCU_A inhibits tumor aggressiveness by controlling a signaling cascade which encompasses mitochondrial- Ca^{2+} dynamics, bioenergetics, and ROS production. Based on this model, increased MCU_A expression causes elevated $_{mito}Ca^{2+}$, increased ATP synthesis but also higher ROS production that ultimately causes oxidative stress. Through a regulation of several pathways and cellular functions, this transformation induces a switch that drives melanoma cells toward a more proliferative and less invasive phenotype. Moreover, this MCU-Ca^{2+}-ATP-ROS axis-induced phenotypic transformation will influence the immunogenicity and the therapeutic sensitivity of melanoma cells. According to this concept, melanomas with high MCU_A abundance/activity are associated with higher therapeutic sensitivity and will manifest into a less aggressive disease (Fig 8).

Within this study, we demonstrate that MCU_A controls melanoma aggressive behavior and therapeutic sensitivity. We identify novel biomarkers and signaling pathways that control melanoma cell aggressiveness and suggest that manipulations of mitochondrial Ca^{2+} and redox homeostasis, in combination with current therapies, should be considered in treating advanced melanoma.

Materials and Methods

Cell culture and reagents

Human melanoma cell lines (1205Lu, 451Lu, 451Lu-BR3, WM983B, WM983B-BR, and WM3734) and HEK293T cells for lentiviral production were provided by Meenhard Herlyn (The Wistar Institute, Philadelphia, USA). Cells were cultured in TU 2% medium (80% MCDB153 basal medium (#P04-80062, PAN Biotech), 20% Leibovitz's L-15 medium (#P04-27055, PAN Biotech) supplemented with 2 mM L-Glutamin, 1.68 mM $CaCl_2$ and 2% FCS) and were maintained at 37°C in 5% CO_2. All cell lines tested negative for Mycoplasma using regularly the PCR Mycoplasma Test Kit I/C (PromoKine #PK-CA91-1048, PromoCell GmbH, Heidelberg, Germany). Chemicals were purchased from Sigma-Aldrich (Munich, Germany), unless otherwise indicated. Small molecule inhibitors were purchased from Selleckchem (Absource, Munich, Germany).

Protein knockdown

Transient KD was performed using siRNA sequences from Qiagen (Hilden, Germany) and MicroSynth (Balgach, Switzerland), delivered to the cells by nucleofection (Amaxa Nucleofector, Lonza GmbH, Cologne, Germany). Two million cells and 4 µl of 20 pmol siRNA were used for each transfection. Measurements were taken 48 h post-transfection. All siRNA sequences are listed in Table EV1.

For stable KD, plasmids expressing shRNA (pLKO.1) were obtained from the Broad Institute's RNAi Consortium (Sigma-Aldrich, St. Louis, MO, USA). Lentiviral vector pLKO.1 was used as an empty vector control (shCTRL) or with the shRNA sequences as follows: shMCU_1 (#TRCN133861) and shMCU_2 (#TRCN420533). Lentiviruses were produced by transfecting HEK293T cells with the packaging plasmids (pPAX2 and pMD2.G) along with 4 µg lentiviral shRNA vector using Lipofectamine 2000 reagent (#116680, Invitrogen, Waltham, MA, USA), according to the manufacturer's instructions. Melanoma cells were transduced with virus in the presence of 8 µg/ml polybrene (Sigma-Aldrich, St. Louis, MO, USA) for 18 h. Transduced cell populations were selected with 1 µM puromycin (Sigma-Aldrich, St. Louis, MO, USA). shRNA KD efficiency was confirmed by WB analysis and RT–qPCR.

Fura-2-AM-based Ca^{2+} imaging

Cytosolic Ca^{2+} measurements were performed as described in (Saul *et al*, 2016). Shortly, melanoma cells were loaded with 1 µM Fura-2 AM (#F1221, Thermo Fisher Scientific GmbH, Schwerte, Germany) in culture medium and incubated at room temperature for 30 min. All measurements were taken in Ringer's buffer (pH = 7.4) containing 145 mM NaCl, 4 mM KCl, 10 mM glucose, 10 mM HEPES (4-(2-hydroxyethyl)-1-piperazineethanesulfonic acid), 2 mM $MgCl_{2,}$ and 0–0.5 mM Ca^{2+} (the 0 mM $CaCl_2$ buffer was supplemented with

 © 2022 The Authors

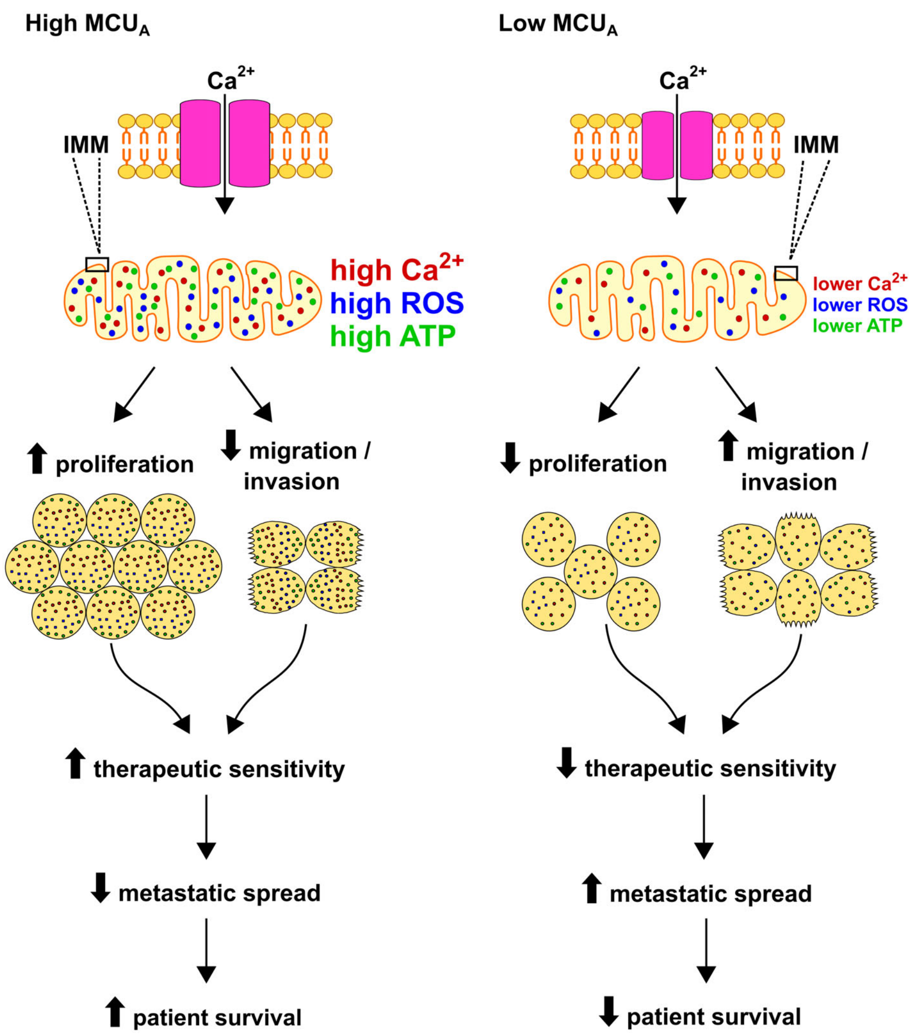

Figure 8. MCU controls melanoma aggressive behavior via regulation of mitochondrial calcium, ATP and redox signals.
High MCU$_A$ abundance results in elevated $_{mito}Ca^{2+}$, which in turn enhances mitochondrial ROS and ATP production. This Ca^{2+}-induced alterations induce metabolic and redox reprograming of melanoma cells and promote a more proliferative yet less invasive cellular phenotype. The high $_{mito}Ca^{2+}$-induces phenotype sensitizes melanoma cells to therapeutic treatments, decreases metastatic spread and has a positive effect on melanoma patient survival. Conversely, low MCU$_A$ abundance causes lower $_{mito}Ca^{2+}$, lower ROS and ATP production, promotes a less proliferative yet more invasive melanoma cell phenotype, promotes melanoma cell resistance to therapeutic treatments and decreases patient survival rate.

1 mM EGTA). One μM thapsigargin (Tg) (#T9033, Sigma-Aldrich, Munich, Germany) and 4 μM ionomycin (Iono) (#407950, Merck Millipore Calbiochem, Burlington, MA, USA) were used to facilitate Ca^{2+} store depletion. Time-lapse ratiometric imaging was carried out using an Olympus IX70 microscope equipped with Xenon-lamp, Polychrome V Monochromator, and CCD-Kamera T.I.L.L. Imago (Olympus, Hamburg, Germany).

Fluorescence microscopy

Imaging experiments were performed at 37°C in 0.5 mM or 1 mM Ca^{2+} Ringer's buffer (as indicated in the figure legends) using either a Zeiss Cell Observer Z1 microscope equipped with a 40× oil "Fluar" (N.A.: 1.3) objective, multi-filter system, fast acquisition EMCCD camera (Evolve® 512 Delta) and LED system (Colibri, Zeiss) or a

© 2022 The Authors

Zeiss Observer D1 equipped with a 40X oil Neofluar (N.A.: 1.3) objective, Axiocam 702 mono and LED system (Colibri, Zeiss) or a Zeiss Axio Observer 7 equipped with 40× oil "Neofluar" (N.A.: 1.3), Axiocam 702 mono and LED system (Colibri 7, Zeiss). Subsequent obtained data were processed with the AxioVision, Zen 2.6, or Zen 3.2 softwares (Zeiss, Oberkochen, Germany). All plasmids and constructs used are listed in Table EV2.

FRET measurements

Mitochondrial Ca^{2+} (4mt-D_3cpV) and mitochondrial ATP (mito-A_{TEAM}) were measured using fluorescence resonance energy transfer (FRET) sensors (CFP excitation: 420/40 nm; emission: 483/32 nm and YFP excitation: 505/15 nm; emission: 542/27 nm). Ca^{2+} uptake was initiated using 1 μM thapsigargin (Tg) or 100 μM ATP. FRET was calculated using the background and bleed-through corrected FRET/donor ratio (equation 1).

$$FRET/donor\ ratio = \frac{(FRET - background) - [(donor - background)\cdot CFd] - [(acceptor - background)\cdot CFa]}{(donor - background)}. \quad (1)$$

*CF: correction factor for donor (d) and acceptor (a) bleed-through.

H_2O_2 and glutathione measurements

Hydrogen peroxide (H_2O_2) measurements were carried out using the ratiometric protein sensor HyPer (excitation: 420/40 and 505/15 nm; emission: 542/28 or 539/25 nm). SypHer sensor was used as a pH sensitivity control. Glutathione redox potential was assessed using the mito-Grx1-roGFP2 ratiometric sensor (excitation 385/30 and 469/28 nm; emission 525/50 nm).

TMRE

Mitochondrial membrane potential (ΔΨ) was measured with TMRE (Tetramethylrhodamine, Ethyl Ester, Perchlorate; #T669; ThermoFisher) on a single cell level. Cells were loaded with 100 nM TMRE for 15 min and imaged (excitation: 550/32 nm; emission: 630/92 nm), 1 μM CCCP (carbonylcyanid-3-chlorophenylhydrazone) was added as a control.

ATP measurements

Mitochondrial ATP was measured using the BioTracker™ ATP-Red Live Cell dye (# SCT045; Merck) on a single cell level. Cells were loaded with 5 μM ATP-Red dye for 15 min at 37°C and imaged (excitation: 550/32 nm; emission: 630/92 nm).

Electron microscopy

Cells grown on glass coverslips were immobilized using a prewarmed solution consisting of 2.5% glutaraldehyde in 0.1 M cacodylic buffer at pH 7.4. Sample immobilization was continued for 1 h at room temperature and was completed at 4°C overnight. Staining and secondary fixation was started using 1% osmium tetroxide in 1.5% $K_4[Fe(CN)_6]$ in 0.1 M cacodylic buffer at pH 7.4 for 1 h at room temperature. Without any washing in-between, the solution was replaced with 1% osmium tetroxide in 0.1 M cacodylic buffer at pH 7.4 and incubated for an additional hour at room temperature. During three extensive washing steps using double distilled water for 10 min each, the Walton's lead aspartate solution was prepared. Therefore, 40 mg aspartic acid was dissolved in double distilled water, and the solution brought up to 60°C in an oven. Next, 66 mg lead nitrate was added to the solution. The pH of the mixture was then adjusted using 1N sodium hydroxide solution. The samples were then immersed in the lead aspartate solution and kept at 60°C for 1 h. After three washing steps with double distilled water for 10 min each, the samples were dehydrated with a graded ethanol series starting with 30% over 50, 70 up to 100% with several exchanges in-between. Final dehydration prior to resin infiltration was facilitated with two exchanges of propylene oxide for 5 min each. Resin infiltration was mediated with a starting solution of 1:1 Epon resin and propylene oxide for 1 h at room temperature followed by placing the samples into fresh 100% Epon resin for an additional hour. For overnight infiltration, the samples were placed again in fresh Epon resin on a rocker table overnight at room temperature. The following day, the samples were embedded in BEEM capsules, and resin polymerization took place over 48 h at 60°C.

Thin sections of 60 nm thickness were prepared from the final resin blocks and collected on formvar-coated mesh grids. The electron micrographs were recorded on a Philips CM120 transmission electron microscope equipped with a LaB6-source and a TVIPS 2x2 slow-scan CCD camera at an original magnification of 8,600×.

Immunolabeling and confocal microscopy

Cells were cultured on glass coverslips and fixed with prewarmed (37°C) 8% formaldehyde in PBS for 10 min at room temperature. After washing with PBS, cells were permeabilized with 0.5% Triton X-100 in PBS for 5 min and blocked with 5% BSA in PBS for 5 min. Anti-TOMM20 antibody coupled to Alexa Fluor 488 (1:100, #ab205486, Abcam) was diluted in 5% BSA in PBS and incubation was performed in dark for 1 hour at room temperature. Cells were washed in PBS and 2.5 μg/ml 40,6-Diamidin-2-phenylindol (DAPI) (Sigma-Aldrich) was added to the PBS during one washing step. Cells were then mounted in Mowiol with 0.1% 1,4-Diazabicyclo [2.2.2]octan (DABCO).

Confocal microscopy was performed with a TCS SP8 (Leica, Wetzlar, Germany) with a 63X oil immersion objective (HC PL APO 63×/1,40 oil CS2). No image processing was applied except for adjustment of brightness and contrast.

Proliferation, viability, migration, and invasion assays

Melanoma cell proliferation was evaluated by seeding 10,000 cells/well in 24-well plates and incubating them at 37°C over a period of 72 h. Following cell growth, cells were fixed with methanol for 15 min and stained with a 0.05% crystal violet solution for 30 min at room temperature. Next, cells were destained with a 40% acetic acid solution, and absorbance at 595 nm was measured using a

 © 2022 The Authors

Mithras LB 940 plate reader (Berthold Technologies, Bad Wildbad, Germany).

Cell viability was assessed using the CellTiter-Blue® Cell Viability Assay kit (Promega GmbH, Walldorf, Germany), according to the manufacturer's instructions. Briefly, 5,000 cells/well were seeded in 96-well plates and allowed to attach overnight. Cells were afterward treated with small molecule inhibitors or ferroptosis inducers for 24–72 h. The resazurin-based reagent was added 3 h prior to fluorescence measurement using a CLARIOstar® plate reader (BMG LABTECH, Ortenberg, Germany).

To assess the *in vitro* migration ability of cells, a transwell migration setup featuring 8 µm pore size inserts (Corning®, Kennebunk ME, USA) was used. Briefly, 200,000 cells in 150 µl of fresh, serum-free medium were pipetted into the top compartment of an insert and were allowed to migrate for 4–48 h toward preconditioned medium containing 10% FCS. Prior to imaging, nonmigrated cells were washed out from the inserts, and the remaining migrated cells were stained with either 1 µM calcein-AM (#C1430, Thermo Fisher Scientific, Waltham, Massachusetts, USA) or with 0.5 µg/ml Hoechst 33342 (#H1399, Thermo Fisher Scientific, Waltham, Massachusetts, USA). Fluorescence intensity was measured using an Infinite 200 PRO plate reader (Tecan, Männedorf, Switzerland), or images of migrated cells were acquired using a Zeiss Axiovert S100TV inverted microscope (Oberkochen, Germany) featuring a sCMOS pco.edge camera and later analyzed using the ImageJ software.

Spheroids were generated as previously described (Roesch *et al*, 2013). Shortly, 5,000 cells/well were seeded in 96-well plates on top of a nonadhesive layer of agar. Following 96 h and the formation of a 3D structure, spheroids were harvested and embedded in a collagen I mixture and were allowed to invade for a given period of time. Spheroids were then stained with the Live/Dead™ Viability/Cytotoxicity Kit (#L3224, Invitrogen, Carlsbad, California, USA) and were imaged using a Zeiss Primo Vert and a Zeiss Axiovert S100TV microscope equipped with a 10× objective. Images were acquired using the AxioVision and the VisiView® softwares. Spheroid size evaluation was determined using the ImageJ software. Spheroid invasion was measured by subtracting the mask for the core of each spheroid from the total area covered by all the cells of a given spheroid (invasion area [μm^2] = total area − spheroid core) using ImageJ.

RT–qPCR

The total RNA (800 ng of template RNA was used per condition) was isolated and reverse transcribed to cDNA using Superscript™ II (#18064022, Invitrogen), according to the manufacturer's instructions. 0.5 µl of cDNA was used for RT–qPCR using the QuantiTect SYBR Green Kit (#204145, Qiagen) and Bio-Rad CFX96™ Real-Time System. The CT values of the target mRNAs were normalized to the CT values of TATA box binding protein (TBP) which was used as the housekeeping gene. Data were quantified using the $2^{-\Delta CT}$ method. Gene-specific primer sets were purchased from Qiagen or Sigma-Aldrich. Primer sequences are listed in Table EV3.

Western blots

For Western blot (WB), proteins were extracted as previously described in (Stanisz *et al*, 2014). Shortly, 40–75 µg of protein were resolved on a 10% SDS–polyacrylamide gel and transferred onto a 0.45 µm nitrocellulose membrane (#10600003, Amersham Protran Premium, GE Healthcare). After transfer, membranes were blocked in 5% BSA or 5% skim milk powder solution, followed by an overnight incubation with primary antibodies. Membranes were incubated with secondary antibodies for 1 h in the dark at room temperature. Imaging and quantification of the blots was performed using an Odyssey infrared imaging system (LI-COR, Lincoln, Nebraska, USA). Primary and secondary antibodies used for immunoblotting are listed in Tables EV4 and EV5.

Real-time killing assay

Human primary NK cell isolation and the subsequent melanoma killing assay were performed as described in (Cappello *et al*, 2021). Briefly, NK cells were obtained by negative bead isolation (#11349D, Dynabeads™ Untouched Human NK cell Kit) from human peripheral blood mononuclear cells (PBMCs) of healthy thrombocyte donors of the local blood bank (University Medical Center Göttingen, Ethics approval 2/3/18). Melanoma cells were seeded in a black, clear bottom 96-well plates and loaded with 0.5 µM calcein-AM (#C1430, Thermo Fisher Scientific, Waltham, MA, USA). Interleukin-2-stimulated (0.05 µg/ml; #15596–026, Thermo Fisher Scientific GmbH, Schwerte, Germany) primary human NK cells were added to melanoma cells in a NK cell to target cell ratio of ~ 5:1. NK cell-mediated melanoma killing was measured for 4 h at 37°C in 5% CO_2 using a CLARIOstar® plate reader (BMG LABTECH, Ortenberg, Germany). Killing efficiency was evaluated by fluorescence signal decrease.

Melanoma xenografts

All animal experiments were performed in accordance with The Wistar IACUC in NOD/LtSscidIL2Rγnull mice (NSG). Mice were kept in the same room, in germ-free environment, socially caged as five mice/unit cage and fed with sterile food pellets and water. Wistar Animal Facility has a quality control program in place wherein 5% of mice in each holding room were periodically tested serologically for common murine viruses, Mycoplasma pulmonis and Helicobacter. Human melanoma cells were injected with the MCU_{A_KD} cells and their respective control (1205Lu shCTRL, shMCU_1 or shMCU_2); 10 animals were used per group, randomized. Each mouse was inoculated subcutaneously with 400,000 melanoma cells in a 1:1 suspension of Matrigel (#354230, BD Matrigel™ Basement Membrane Matrix, Growth Factor Reduced; Becton Dickinson, Franklin Lakes, NJ, USA) and complete media. Seven days after cell injection, tumor growth was measured every 2–3 days, for 36 days, using a caliper and volumes calculated according to the formula $V = (W \times D \times H)/2$ (mm^3). On the last experimental day, a final measurement of the tumor was taken before tumor extraction. Other organs such as liver, lung, kidneys, and brain were isolated for further examinations. Tumor samples were snap-frozen in liquid nitrogen for subsequent protein analyses or fixed in formalin for the generation of paraffin blocks for immunostaining.

Immunohistochemistry

Hematoxylin and eosin (H&E) staining was performed on sections of lung tissue from mice; these were deparaffinized and returned to

an aqueous medium by consecutively immersing them in xylene, 100% isopropanol, and 70% isopropanol. For the hematoxylin staining, a hemalum solution diluted 1:10 in water was prepared. Following dipping for 6–10 times into the hemalum solution, tissue sections were rinsed with tap water which resulted in a color change from reddish-brown to blue-violet. Tissue sections were consequently counterstained with eosin and were scanned using an Axio Scan.Z1 microscope.

Reverse Phase Protein Array (RPPA)

The RPPA assay was performed by the MD Anderson Center RPPA core facility, as previously described (Paweletz *et al*, 2001; Grote *et al*, 2008). Shortly, serial-diluted lysates were printed on nitrocellulose-coated slides using a 2470 Microarray printer from Aushon Biosystems (Billerica, Massachusetts, USA). Slides were probed with primary antibodies plus biotin-conjugated secondary antibodies. The signal obtained was amplified using a Dako signal amplification system (Copenhagen, Denmark) and visualized by DAB colorimetric reaction. The intensity of each spot was calculated using the MicroVigeneTM (VigeneTech, Billerica, Massachusetts, USA). The data obtained contained normalized and linearized values of protein. The values of the KD cells were normalized to the associated control. Primary and secondary antibodies used for the RPPA assay can be found on the website of the MD Anderson Cancer Center (https://www.mdanderson.org/research/research-resources/core-facilities/functional-proteomics-rppa-core/antibody-information-and-protocols.html).

Mass spectrometric proteome analysis

A total of 50 µg of protein per sample were loaded onto a 4–12% NuPAGE Novex Bis-Tris Minigels (Invitrogen) and run into the gel for 1.5 cm. Following Coomassie staining, the protein areas were cut out, diced, and reduced with dithiothreitol, alkylation with iodoacetamide, and digested overnight with trypsin. Tryptic peptides were extracted from the gel, the solution dried in a Speedvac, and kept at −20°C for further analyses (Atanassov & Urlaub, 2013). For the generation of a peptide library, equal amount of aliquots from each sample were pooled to the total amount of 80 µg, and separated into eight fractions using a reversed phase spin column (Pierce High pH Reversed-Phase Peptide Fractionation Kit, Thermo Fisher Scientific).

Digested proteins were analyzed on a nanoflow chromatography system (Eksigent nanoLC425) hyphenated to a hybrid triple quadrupole-TOF mass spectrometer (TripleTOF 5600+) equipped with a Nanospray III ion source (Ionspray Voltage 2400 V, Interface Heater Temperature 150°C, Sheath Gas Setting 12) and controlled by Analyst TF 1.7.1 software build 1163 (all AB Sciex). In brief, peptides were dissolved in loading buffer (2% acetonitrile, 0.1% formic acid in water) to a concentration of 0.3 µg/µl. For each analysis, 1.5 µg of digested protein was enriched on a self-packed precolumn (0.15 mm ID x 20 mm, Reprosil-Pur120 C18-AQ 5 µm, Dr. Maisch, Ammerbuch-Entringen, Germany) and separated on an analytical RP-C18 column (0.075 mm ID x 250 mm, Reprosil-Pur 120 C18-AQ, 3 µm, Dr. Maisch) using a 100-min linear gradient of 5–35% acetonitrile/0.1% formic acid (v:v) at 300 nl min^{-1}.

Qualitative LC/MS/MS analysis was performed using a Top30 data-dependent acquisition method with an MS survey scan of *m/z* 380–1,250 accumulated for 250 ms at a resolution of 35,000 full width at half maximum (FWHM). MS/MS scans of *m/z* 180–1,500 were accumulated for 100 ms at a resolution of 17,500 FWHM and a precursor isolation width of 0.7 FWHM, resulting in a total cycle time of 3.4 s. Precursors above a threshold MS intensity of 200 cps with charge states 2+, 3+, and 4+ were selected for MS/MS, and the dynamic exclusion time was set to 15 s. MS/MS activation was achieved by CID using nitrogen as a collision gas and the manufacturer's default rolling collision energy settings. Two technical replicates per reversed phase fraction were analyzed to construct a spectral library.

For quantitative SWATH analysis, MS/MS data were acquired using 100 variable size windows across the 400–1,200 *m/z* range (Zhang *et al*, 2015). Fragments were produced using rolling collision energy settings for a charge state of 2+, and fragments acquired over an *m/z* range of 180–1,500 for 40 ms per segment. An overall cycle time of 4.3 s was the result of a 250 ms survey scan. Two replicate injections were acquired for each biological sample.

Protein identification was achieved using ProteinPilot Software version 5.0 build 4769 (AB Sciex) at the appropriate settings. A total of 126,301 MS/MS spectra from the combined qualitative analyses were searched against the UniProtKB *Homo sapiens* reference proteome (revision 04–2018, 93,610 entries) augmented with a set of 51 known common laboratory contaminants to identify proteins at a false discovery rate (FDR) of 1%.

Spectral library generation and SWATH peak extraction were achieved in PeakView Software version 2.1 build 11041 (AB Sciex) using the SWATH quantitation microApp version 2.0 build 2003. Following retention time correction on endogenous peptides spanning the entire retention time range, peak areas were extracted using information from the MS/MS library at a FDR of 1% (Lambert *et al*, 2013). The resulting peak areas were then summed to peptide and finally protein area values, which were used for further statistical analyses. A 1.698 proteins could be quantified consistently across all samples at a FDR of 1%

Bioinformatic analyses

Patient survival data

To generate survival plots, melanoma patient datasets (TCGA) were downloaded from cBioPortal on 26.08.2020. Data on other cancers were downloaded from Human Protein Atlas on 18.03.2021. Kaplan–Meier plots were calculated using lifelines (10.5281/zenodo.4816284). Patients were divided into patients with either low or high MCU_A expression by scanning 20–80% of MCU_A's expression value and determining the optimal separation in terms of survival based on a log-rank test. For detailed analyses, patients were separated by different melanoma tumor stages (I–II and III–IV), by age, gender, or by BRAF- and NRAS-wild-type or -mutant. Melanoma patient survival data (low and high MCU_A) were utilized for further analyses (based on hits evaluation). Hits were determined by calculating *P*-values (< 0.001) using Wilcoxon test adjusted by the Benjamini–Hochberg procedure, (FDR < 0.05) and selected according to an absolute logarithmic fold change higher than 1.

Volcano plots

Volcano plots were created to display protein hits. Student's *t*-tests were calculated for each shCTRL and MCU_{A_KD} cell line

 © 2022 The Authors

independently. Hits were shown in red if the unadjusted *P*-value was significant ($P < 0.05$) and the absolute logarithmical fold change was higher than 0.5. Hits were shown in green if the adjusted (Benjamini–Hochberg procedure, FDR < 0.05) *P*-value was significant and an absolute logarithmical fold change higher than 1 occurred. Only the hits shown in red in the volcano plot were further analyzed.

RPPA heatmap

To assess the RPPA data, a heatmap was generated. Hits were selected by considering a logarithmical fold change higher than 1 for each MCU_{A_KD} (WM3734 shMCU_1, WM3734 shMCU_2, 1205Lu shMCU_1, 1205Lu shMCU_2) cell line independently.

Proteomaps

Proteomaps were generated via (http://bionic-vis.biologie.uni-greifswald.de/) by importing patient data-, proteomic-, and RPPA hits. All proteomaps were generated considering all human genes as a background. The size of each polygon represents the protein abundance.

KEGG-based enrichment analyses

KEGG (Kyoto encyclopedia of genes and genomes)-based enrichment analysis were generated using DAVID bioinformatics resources that analyzed patient data-, proteomic-, and RPPA hits (red) (Huang da *et al*, 2009). All results were filtered by FDR adjusted *P*-value threshold of 0.05 and sorted by fold enrichment.

GO Terms

Patient data-, proteomic-, and RPPA hits were analyzed using GO (Gene Ontology) terms (Ashburner *et al*, 2000). All results were filtered by FDR adjusted *P*-value threshold of 0.05 and sorted by fold enrichment.

Hallmarks

Cancer Hallmarks Gene sets were taken from (Zhang *et al*, 2020). Proteomic- and RPPA hits were analyzed considering their occurrence in each gene set. The occurrence of each hallmark was calculated and shown in percentage. *P*-values were calculated using hygeometric tests.

Statistical analyses

Data were analyzed and processed using Zeiss AxioVision, Zeiss Zen, VisiView®, TILLVISION, Bio-Rad Quantity One, ImageJ/FIJI and Microsoft Excel. Data are shown as mean ± SEM. Unpaired, two-tailed Student's *t*-tests were carried out to test statistical significance, unless otherwise specified. The significant differences are marked with asterisks: $^*P < 0.05$, $^{**}P < 0.01$, and $^{***}P < 0.005$; a *P*-value higher than 0.05 means that no significant difference was observed.

Data availability

The datasets produced in this study are available in the following databases:
RPPA: These data are available in Dataset EV3.
Mass spectrometry proteomics: These data are available in Datasets EV1 and EV2 and on the ProteomeXchange Consortium via the PRIDE partner repository, dataset identifier: PXD029132 (http://www.ebi.ac.uk/pride/archive/projects/PXD029132).

Expanded View for this article is available online.

Acknowledgments

We thank Prof. Markus Hoth for his continuous support. Many thanks also to Sandra Janku, Alexandra Stark, Andrea Paluschkiwitz, Josh Wang, Annette Bennemann, Sonja Schwoch, and Berti Manshausen for their technical assistance. This work was supported by German Research Foundation (DFG) projects: RO 3577/3-2 (to A.R.), RO 3577/7-1 (to A.R.), SFB1430 project A11 (to A.R.), SFB1190 projects 1 (to S.J.), 13 (to P.R.) and 17 (to I.B.), SFB1027 Project C4 (to I.B.), IRTG1816 (to I.B.), TRR 274 Z01 (to S.J.). H.S. acknowledges financial support from the Medical School, University of Saarland via the HOMFOR program. Open Access funding enabled and organized by Projekt DEAL.

Author contributions

Ioana Stejerean-Todoran: Formal analysis; investigation; visualization; writing – original draft. **Katharina Zimmermann:** Formal analysis; investigation; visualization. **Christine Gibhardt:** Formal analysis; investigation; visualization. **Adina Vultur:** Formal analysis; supervision; investigation; writing – original draft. **Christian Ickes:** Formal analysis; investigation; visualization. **Batool Shannan:** Formal analysis; investigation. **Zurine Bonilla del Rio:** Formal analysis; investigation. **Anna Wölling:** Formal analysis; investigation. **Sabrina Cappello:** Formal analysis; investigation. **Hsu-Min Sung:** Formal analysis; investigation. **Magdalena Shumanska:** Formal analysis; investigation; visualization. **Xin Zhang:** Formal analysis; investigation. **Maithily Nanadikar:** Formal analysis; investigation. **Muhammad U Latif:** Formal analysis; investigation; visualization. **Anna Wittek:** Formal analysis; investigation; visualization. **Felix Lange:** Formal analysis; investigation; visualization. **Andrea Waters:** Investigation. **Patricia Brafford:** Investigation. **Jörg Wilting:** Formal analysis; supervision; investigation. **Henning Urlaub:** Resources; supervision; funding acquisition. **Dörthe M Katschinski:** Supervision; funding acquisition. **Peter Rehling:** Supervision; funding acquisition. **Christof Lenz:** Supervision; investigation; methodology. **Stefan Jakobs:** Conceptualization; funding acquisition. **Volker Ellenrieder:** Conceptualization; supervision; funding acquisition. **Alexander Roesch:** Supervision; funding acquisition. **Michael P Schön:** Supervision; funding acquisition. **Meenhard Herlyn:** Resources; supervision; funding acquisition. **Hedwig Stanisz:** Conceptualization; supervision; funding acquisition; writing – original draft; writing – review and editing. **Ivan Bogeski:** Conceptualization; supervision; funding acquisition; writing – original draft; writing – review and editing.

Disclosure and competing interests statement

The authors declare that they have no conflict of interest.

References

Arduino DM, Wettmarshausen J, Vais H, Navas-Navarro P, Cheng Y, Leimpek A, Ma Z, Delrio-Lorenzo A, Giordano A, Garcia-Perez C *et al* (2017) Systematic identification of MCU modulators by orthogonal interspecies chemical screening. *Mol Cell* 67: 711–723.e717

Ashburner M, Ball CA, Blake JA, Botstein D, Butler H, Cherry JM, Davis AP, Dolinski K, Dwight SS, Eppig JT *et al* (2000) Gene ontology: tool for the unification of biology. The Gene Ontology Consortium. *Nat Genet* 25: 25–29

Atanassov I, Urlaub H (2013) Increased proteome coverage by combining PAGE and peptide isoelectric focusing: comparative study of gel-based separation approaches. *Proteomics* 13: 2947–2955
Barceló C, Sisó P, Maiques O, de la Rosa I, Martí RM, Macià A (2020) T-type calcium channels: a potential novel target in melanoma. *Cancer* 12: 391
Bondarenko AI, Parichatikanond W, Madreiter CT, Rost R, Waldeck-Weiermair M, Malli R, Graier WF (2015) UCP2 modulates single-channel properties of a MCU-dependent Ca(2+) inward current in mitochondria. *Pflugers Arch* 467: 2509–2518
Booth DM, Várnai P, Joseph SK, Hajnóczky G (2021) Oxidative bursts of single mitochondria mediate retrograde signaling toward the ER. *Mol Cell* 81: 3866–3876.e3862
Cappello S, Sung HM, Ickes C, Gibhardt CS, Vultur A, Bhat H, Hu Z, Brafford P, Denger A, Stejerean-Todoran I *et al* (2021) Protein signatures of NK cell-mediated melanoma killing predict response to immunotherapies. *Cancer Res* 81: 5540–5554
Cesi G, Walbrecq G, Zimmer A, Kreis S, Haan C (2017) ROS production induced by BRAF inhibitor treatment rewires metabolic processes affecting cell growth of melanoma cells. *Mol Cancer* 16: 102
Chio IIC, Tuveson DA (2017) ROS in cancer: The Burning Question. *Trends Mol Med* 23: 411–429
De Mario A, Tosatto A, Hill JM, Kriston-Vizi J, Ketteler R, Vecellio Reane D, Cortopassi G, Szabadkai G, Rizzuto R, Mammucari C (2021) Identification and functional validation of FDA-approved positive and negative modulators of the mitochondrial calcium uniporter. *Cell Rep* 35: 109275
Di Marco G, Vallese F, Jourde B, Bergsdorf C, Sturlese M, De Mario A, Techer-Etienne V, Haasen D, Oberhauser B, Schleeger S *et al* (2020) A high-throughput screening identifies MICU1 targeting compounds. *Cell Rep* 30: 2321–2331.e2326
Diebold L, Chandel NS (2016) Mitochondrial ROS regulation of proliferating cells. *Free Radic Biol Med* 100: 86–93
Foskett JK, Philipson B (2015) The mitochondrial Ca(2+) uniporter complex. *J Mol Cell Cardiol* 78: 3–8
Grote T, Siwak DR, Fritsche HA, Joy C, Mills GB, Simeone D, Whitcomb DC, Logsdon CD (2008) Validation of reverse phase protein array for practical screening of potential biomarkers in serum and plasma: accurate detection of CA19-9 levels in pancreatic cancer. *Proteomics* 8: 3051–3060
Hanahan D, Weinberg RA (2011) Hallmarks of cancer: the next generation. *Cell* 144: 646–674
Haq R, Shoag J, Andreu-Perez P, Yokoyama S, Edelman H, Rowe GC, Frederick DT, Hurley AD, Nellore A, Kung AL *et al* (2013) Oncogenic BRAF regulates oxidative metabolism via PGC1alpha and MITF. *Cancer Cell* 23: 302–315
Harel M, Ortenberg R, Varanasi SK, Mangalhara KC, Mardamshina M, Markovits E, Baruch EN, Tripple V, Arama-Chayoth M, Greenberg E *et al* (2019) Proteomics of melanoma response to immunotherapy reveals mitochondrial dependence. *Cell* 179: 236–250.e218
Hempel N, Trebak M (2017) Crosstalk between calcium and reactive oxygen species signaling in cancer. *Cell Calcium* 63: 70–96
Hoffman NE, Chandramoorthy HC, Shanmughapriya S, Zhang XQ, Vallem S, Doonan PJ, Malliankaraman K, Guo S, Rajan S, Elrod JW *et al* (2014) SLC25A23 augments mitochondrial Ca^{2+} uptake, interacts with MCU, and induces oxidative stress-mediated cell death. *Mol Biol Cell* 25: 936–947
Hooper R, Zhang X, Webster M, Go C, Kedra J, Marchbank K, Gill DL, Weeraratna AT, Trebak M, Soboloff J (2015) Novel protein kinase C-mediated control of orai1 function in invasive melanoma. *Mol Cell Biol* 35: 2790–2798
Hoth M (2016) CRAC channels, calcium, and cancer in light of the driver and passenger concept. *Biochim Biophys Acta* 1863: 1408–1417
Hoth M, Fanger CM, Lewis RS (1997) Mitochondrial regulation of store-operated calcium signaling in T lymphocytes. *J Cell Biol* 137: 633–648
Huang da W, Sherman BT, Lempicki RA (2009) Bioinformatics enrichment tools: paths toward the comprehensive functional analysis of large gene lists. *Nucleic Acids Res* 37: 1–13
Jenkins RW, Fisher DE (2020) Treatment of advanced melanoma in 2020 and beyond. *J Invest Dermatol* 141: 23–31
Jiang X, Stockwell BR, Conrad M (2021) Ferroptosis: mechanisms, biology and role in disease. *Nat Rev Mol Cell Biol* 22: 266–282
Joseph SK, Booth DM, Young MP, Hajnóczky G (2019) Redox regulation of ER and mitochondrial Ca(2+) signaling in cell survival and death. *Cell Calcium* 79: 89–97
Kapałczyńska M, Kolenda T, Przybyła W, Zajączkowska M, Teresiak A, Filas V, Ibbs M, Bliźniak R, Łuczewski Ł, Lamperska K (2018) 2D and 3D cell cultures: a comparison of different types of cancer cell cultures. *Arch Med Sci* 14: 910–919
Lambert JP, Ivosev G, Couzens AL, Larsen B, Taipale M, Lin ZY, Zhong Q, Lindquist S, Vidal M, Aebersold R *et al* (2013) Mapping differential interactomes by affinity purification coupled with data-independent mass spectrometry acquisition. *Nat Methods* 10: 1239–1245
Le Gal K, Ibrahim MX, Wiel C, Sayin VI, Akula MK, Karlsson C, Dalin MG, Akyurek LM, Lindahl P, Nilsson J *et al* (2015) Antioxidants can increase melanoma metastasis in mice. *Sci Transl Med* 7: 308re8
Li CJ, Lin HY, Ko CJ, Lai JC, Chu PY (2020) A novel biomarker driving poor-prognosis liver cancer: overexpression of the mitochondrial calcium gatekeepers. *Biomedicine* 8: 451
Lin Q-T, Stathopulos PB (2019) Molecular mechanisms of leucine zipper EF-hand containing transmembrane protein-1 function in health and disease. *Int J Mol Sci* 20: 286
Lu Y, Ling S, Hegde AM, Byers LA, Coombes K, Mills GB, Akbani R (2016) Using reverse-phase protein arrays as pharmacodynamic assays for functional proteomics, biomarker discovery, and drug development in cancer. *Semin Oncol* 43: 476–483
Madreiter-Sokolowski CT, Gottschalk B, Sokolowski AA, Malli R, Graier WF (2021) Dynamic control of mitochondrial Ca(2+) levels as a survival strategy of cancer cells. *Front Cell Dev Biol* 9: 614668
Mammucari C, Raffaello A, Vecellio Reane D, Gherardi G, De Mario A, Rizzuto R (2018) Mitochondrial calcium uptake in organ physiology: from molecular mechanism to animal models. *Pflugers Arch* 470: 1165–1179
Marchi S, Giorgi C, Galluzzi L, Pinton P (2020) Ca^{2+} fluxes and cancer. *Mol Cell* 78: 1055–1069
Monteith GR, Prevarskaya N, Roberts-Thomson SJ (2017) The calcium-cancer signalling nexus. *Nat Rev Cancer* 17: 367–380
Nemani N, Shanmughapriya S, Madesh M (2018) Molecular regulation of MCU: implications in physiology and disease. *Cell Calcium* 74: 86–93
O'Donnell JS, Teng MWL, Smyth MJ (2019) Cancer immunoediting and resistance to T cell-based immunotherapy. *Nat Rev Clin Oncol* 16: 151–167
Pallafacchina G, Zanin S, Rizzuto R (2021) From the identification to the dissection of the physiological role of the mitochondrial calcium uniporter: an ongoing story. *Biomolecules* 11: 786
Pampaloni F, Reynaud EG, Stelzer EH (2007) The third dimension bridges the gap between cell culture and live tissue. *Nat Rev Mol Cell Biol* 8: 839–845
Panieri E, Santoro MM (2016) ROS homeostasis and metabolism: a dangerous liason in cancer cells. *Cell Death Dis* 7: e2253
Pasquali S, Hadjinicolaou AV, Sileni VC, Rossi CR, Mocellin S (2018) Systemic treatments for metastatic cutaneous melanoma. *Cochrane Database Syst Rev* 2: CD011123

© 2022 The Authors

Pathak T, Gueguinou M, Walter V, Delierneux C, Johnson MT, Zhang X, Xin P, Yoast RE, Emrich SM, Yochum GS *et al* (2020) Dichotomous role of the human mitochondrial Na(+)/Ca2(+)/Li(+) exchanger NCLX in colorectal cancer growth and metastasis. *Elife* 9: e59686

Pathak T, Trebak M (2018) Mitochondrial Ca(2+) signaling. *Pharmacol Ther* 192: 112–123

Paweletz CP, Charboneau L, Bichsel VE, Simone NL, Chen T, Gillespie JW, Emmert-Buck MR, Roth MJ, Petricoin IE, Liotta LA (2001) Reverse phase protein microarrays which capture disease progression show activation of pro-survival pathways at the cancer invasion front. *Oncogene* 20: 1981–1989

Petrungaro C, Zimmermann KM, Küttner V, Fischer M, Dengjel J, Bogeski I, Riemer J (2015) The Ca(2+)-dependent release of the Mia40-induced MICU1-MICU2 dimer from MCU regulates mitochondrial Ca(2+) uptake. *Cell Metab* 22: 721–733

Piskounova E, Agathocleous M, Murphy MM, Hu Z, Huddlestun SE, Zhao Z, Leitch AM, Johnson TM, DeBerardinis RJ, Morrison SJ (2015) Oxidative stress inhibits distant metastasis by human melanoma cells. *Nature* 527: 186–191

Prevarskaya N, Skryma R, Shuba Y (2011) Calcium in tumour metastasis: new roles for known actors. *Nat Rev Cancer* 11: 609–618

Rambow F, Marine JC, Goding CR (2019) Melanoma plasticity and phenotypic diversity: therapeutic barriers and opportunities. *Genes Dev* 33: 1295–1318

Ribas A, Wolchok JD (2018) Cancer immunotherapy using checkpoint blockade. *Science* 359: 1350–1355

Roesch A, Fukunaga-Kalabis M, Schmidt EC, Zabierowski SE, Brafford PA, Vultur A, Basu D, Gimotty P, Vogt T, Herlyn M (2010) A temporarily distinct subpopulation of slow-cycling melanoma cells is required for continuous tumor growth. *Cell* 141: 583–594

Roesch A, Vultur A, Bogeski I, Wang H, Zimmermann KM, Speicher D, Körbel C, Laschke MW, Gimotty PA, Philipp SE *et al* (2013) Overcoming intrinsic multidrug resistance in melanoma by blocking the mitochondrial respiratory chain of slow-cycling JARID1B(high) cells. *Cancer Cell* 23: 811–825

Samanta K, Douglas S, Parekh AB (2014) Mitochondrial calcium uniporter MCU supports cytoplasmic Ca^{2+} oscillations, store-operated Ca^{2+} entry and Ca^{2+}–dependent gene expression in response to receptor stimulation. *PLoS One* 9: e101188

Saul S, Gibhardt CS, Schmidt B, Lis A, Pasieka B, Conrad D, Jung P, Gaupp R, Wonnenberg B, Diler E *et al* (2016) A calcium-redox feedback loop controls human monocyte immune responses: the role of ORAI Ca^{2+} channels. *Sci Signal* 9: ra26

Sayin VI, Ibrahim MX, Larsson E, Nilsson JA, Lindahl P, Bergo MO (2014) Antioxidants accelerate lung cancer progression in mice. *Sci Transl Med* 6: 221ra15

Schadendorf D, van Akkooi ACJ, Berking C, Griewank KG, Gutzmer R, Hauschild A, Stang A, Roesch A, Ugurel S (2018) Melanoma. *Lancet* 392: 971–984

Stafford WC, Peng X, Olofsson MH, Zhang X, Luci DK, Lu L, Cheng Q, Trésaugues L, Dexheimer TS, Coussens NP *et al* (2018) Irreversible inhibition of cytosolic thioredoxin reductase 1 as a mechanistic basis for anticancer therapy. *Sci Transl Med* 10: eaaf7444

Stanisz H, Saul S, Müller CS, Kappl R, Niemeyer BA, Vogt T, Hoth M, Roesch A, Bogeski I (2014) Inverse regulation of melanoma growth and migration by Orai1/STIM2-dependent calcium entry. *Pigment Cell Melanoma Res* 27: 442–453

Stanisz H, Stark A, Kilch T, Schwarz EC, Müller CS, Peinelt C, Hoth M, Niemeyer BA, Vogt T, Bogeski I (2012) ORAI1 Ca(2+) channels control endothelin-1-induced mitogenesis and melanogenesis in primary human melanocytes. *J Invest Dermatol* 132: 1443–1451

Stanisz H, Vultur A, Herlyn M, Roesch A, Bogeski I (2016) The role of Orai-STIM calcium channels in melanocytes and melanoma. *J Physiol* 594: 2825–2835

Tasdogan A, Faubert B, Ramesh V, Ubellacker JM, Shen B, Solmonson A, Murphy MM, Gu Z, Gu W, Martin M *et al* (2020) Metabolic heterogeneity confers differences in melanoma metastatic potential. *Nature* 577: 115–120

Theodosakis N, Micevic G, Kelly DP, Bosenberg M (2014) Mitochondrial function in melanoma. *Arch Biochem Biophys* 563: 56–59

Tosatto A, Sommaggio R, Kummerow C, Bentham RB, Blacker TS, Berecz T, Duchen MR, Rosato A, Bogeski I, Szabadkai G *et al* (2016) The mitochondrial calcium uniporter regulates breast cancer progression via HIF-1α. *EMBO Mol Med* 8: 569–585

Ubellacker JM, Tasdogan A, Ramesh V, Shen B, Mitchell EC, Martin-Sandoval MS, Gu Z, McCormick ML, Durham AB, Spitz DR *et al* (2020) Lymph protects metastasizing melanoma cells from ferroptosis. *Nature* 585: 113–118

Vazquez F, Lim JH, Chim H, Bhalla K, Girnun G, Pierce K, Clish CB, Granter SR, Widlund HR, Spiegelman BM *et al* (2013) PGC1alpha expression defines a subset of human melanoma tumors with increased mitochondrial capacity and resistance to oxidative stress. *Cancer Cell* 23: 287–301

Vultur A, Gibhardt CS, Stanisz H, Bogeski I (2018) The role of the mitochondrial calcium uniporter (MCU) complex in cancer. *Pflugers Arch* 470: 1149–1163

Zhang D, Huo D, Xie H, Wu L, Zhang J, Liu L, Jin Q, Chen X (2020) CHG: a systematically integrated database of cancer hallmark genes. *Front Genet* 11: 29

Zhang X, Gibhardt CS, Will T, Stanisz H, Körbel C, Mitkovski M, Stejerean I, Cappello S, Pacheu-Grau D, Dudek J *et al* (2019) Redox signals at the ER–mitochondria interface control melanoma progression. *EMBO J* 38: e100871

Zhang Y, Bilbao A, Bruderer T, Luban J, Strambio-De-Castillia C, Lisacek F, Hopfgartner G, Varesio E (2015) The use of variable Q1 isolation windows improves selectivity in LC-SWATH-MS acquisition. *J Proteome Res* 14: 4359–4371

Zheng X, Lu S, He Z, Huang H, Yao Z, Miao Y, Cai C, Zou F (2020) MCU-dependent negative sorting of miR-4488 to extracellular vesicles enhances angiogenesis and promotes breast cancer metastatic colonization. *Oncogene* 39: 6975–6989

License: This is an open access article under the terms of the Creative Commons Attribution License, which permits use, distribution and reproduction in any medium, provided the original work is properly cited.

Expanded View Figures

Figure EV1. MCU_A expression affects cancer patient survival (related to Fig 1).

A–O Kaplan–Meier survival plots depicting the correlation between MCU_A mRNA expression levels and survival probability of stage I-II (A) and stage III-IV (B) melanoma patients and of patients with indicated cancer types (C–O). BLCA, bladder urothelial carcinoma; BRCA, breast invasive carcinoma; CESC, cervical squamous cell carcinoma and endocervical adenocarcinoma; COAD, colon adenocarcinoma; GBM, glioblastoma multiforme; HNSC, head and neck squamous cell carcinoma; LIHC, liver hepatocellular carcinoma; OV, ovarian serous cystadenocarcinoma; PRAD, prostate adenocarcinoma; READ, rectum adenocarcinoma; STAD, stomach adenocarcinoma; TGCT, testicular germ cell tumors; THCA, thyroid carcinoma; UCEC, uterine corpus endometrial carcinoma. *P*-values are determined by log-rank test.

P–T MCU expression levels in wild-type versus BRAF-mutant patients (P) and WT versus NRAS-mutant melanoma patients (Q), in melanoma patients with different tumor stages (R), in melanoma patients of different age intervals (S) and in male versus female melanoma patients (T). NOS, not otherwise specified; TPM, transcripts per million reads.

Data information: Data in (P–T) are presented as mean ± SEM (n = 463 patients).

 © 2022 The Authors

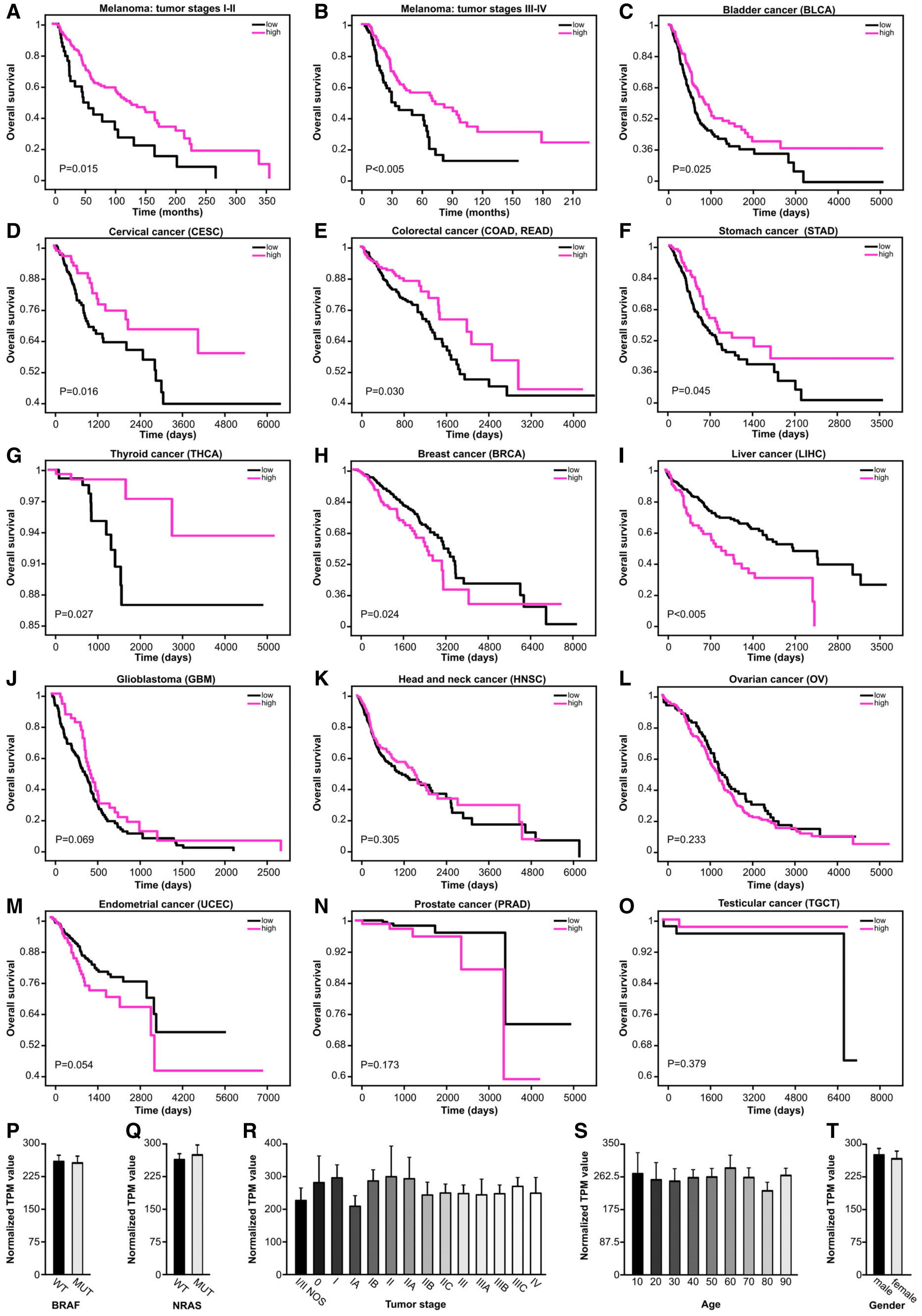
A
Melanoma: tumor stages I-II
P=0.015
Time (months)
B
Melanoma: tumor stages III-IV
P<0.005
Time (months)
C
Bladder cancer (BLCA)
P=0.025
Time (days)
D
Cervical cancer (CESC)
P=0.016
Time (days)
E
Colorectal cancer (COAD, READ)
P=0.030
Time (days)
F
Stomach cancer (STAD)
P=0.045
Time (days)
G
Thyroid cancer (THCA)
P=0.027
Time (days)
H
Breast cancer (BRCA)
P=0.024
Time (days)
I
Liver cancer (LIHC)
P<0.005
Time (days)
J
Glioblastoma (GBM)
P=0.069
Time (days)
K
Head and neck cancer (HNSC)
P=0.305
Time (days)
L
Ovarian cancer (OV)
P=0.233
Time (days)
M
Endometrial cancer (UCEC)
P=0.054
Time (days)
N
Prostate cancer (PRAD)
P=0.173
Time (days)
O
Testicular cancer (TGCT)
P=0.379
Time (days)
Overall survival
low
high
P
Normalized TPM value
WT
MUT
BRAF
Q
Normalized TPM value
WT
MUT
NRAS
R
Normalized TPM value
I/II NOS
0
I
IA
IB
II
IIA
IIB
IIC
III
IIIA
IIIB
IIIC
IV
Tumor stage
S
Normalized TPM value
10
20
30
40
50
60
70
80
90
Age
T
Normalized TPM value
male
female
Gender

Figure EV1.

Figure EV2. Evaluation of siRNA and shRNA-mediated MCU_A depletion efficacy and their influence on store-operated Ca^{2+} entry (related to Fig 2).

A, B mRNA expression of MCU_A in 1205Lu (A) and WM3734 (B) shCTRL and MCU_{A_KD} cells ($n \leq 4$ biological replicates).
C WB for MCU_A in 1205Lu and WM3734 MCU_{A_KD} cells.
D Corresponding band quantification, shown as the ratio between MCU_A and the loading control calnexin ($n = 4$ biological replicates).
E, F mRNA expression of MCU_A in 1205Lu (A) and WM3734 (B) MCU_A transient (siRNA) KD cells ($n \leq 4$ biological replicates).
G WB for MCU_A in 1205Lu and WM3734 transient MCU_{A_KD} cells.
H Corresponding band quantification, shown as the ratio between MCU_A and the loading control calnexin ($n = 3$ biological replicates).
I, J mRNA expression of MCU complex components (MCU_A, MCU_B, MICU1, MICU2, MICU3, EMRE, MCUR1) in 1205Lu ($n \geq 3$ biological replicates) (I) and WM3734 ($n \geq 3$ biological replicates) (J) cells with and without stable MCU_{A_KD}, normalized to housekeeping gene TBP, quantified by RT–qPCR.
K WB for proteins of the MCU complex (MCU_B, MICU1, MICU2, MICU3, MCUR1) in 1205Lu and WM3734 cells with and without stable MCU_{A_KD}. β-actin was used as loading control.
L mRNA expression of MCU complex components (MCU_A, MCU_B, MICU1, MICU2, MICU3, EMRE, and MCUR1) in WM3734 transient MCU_{A_KD} cells, normalized to housekeeping gene TBP, quantified by RT–qPCR ($n = 5$ biological replicates).
M Cytosolic Ca^{2+} measurements in stable WM3734 shCTRL (black), WM3734 shMCU_1 (darker orange) and WM3734 shMCU_2 (lighter orange) cells.
N Corresponding quantification of basal cytosolic Ca^{2+} before thapsigargin (Tg, 1 μM) stimulation, of ΔCa^{2+} ER and of ΔSOCE (shCTRL: $n = 186$ cells from seven biological replicates; shMCU_1: $n = 133$ cells from six biological replicates; shMCU_2: $n = 167$ cells from seven biological replicates).

Data information: Data are presented as mean ± SEM. Statistical significance was assessed using unpaired, two-tailed Student's *t*-test (KD cells were compared with their respective control), *$P < 0.05$; **$P < 0.01$; ***$P < 0.005$; no asterisk means no statistical significance ($P > 0.05$).
Source data are available online for this figure.

 © 2022 The Authors

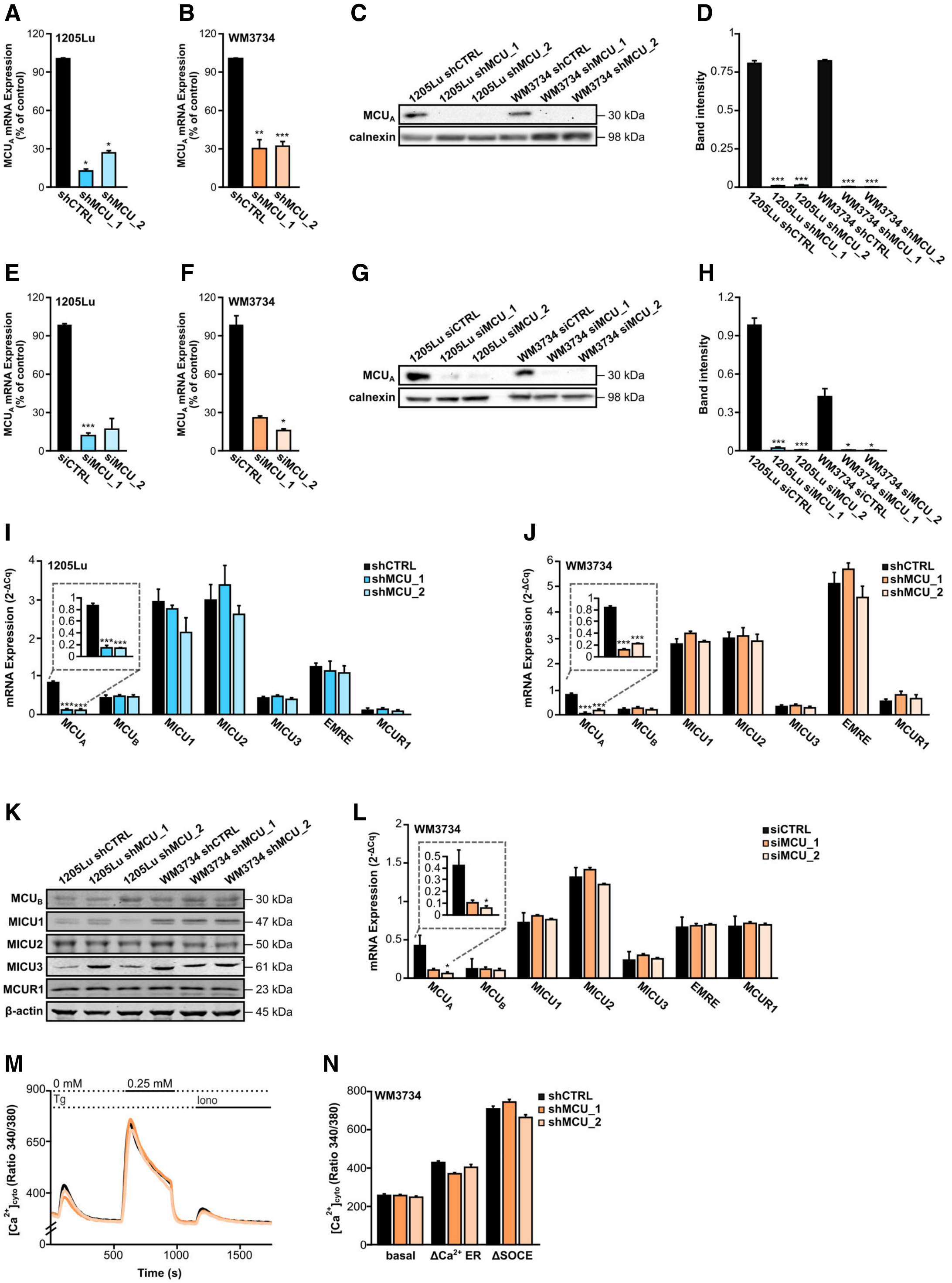

Figure EV2.

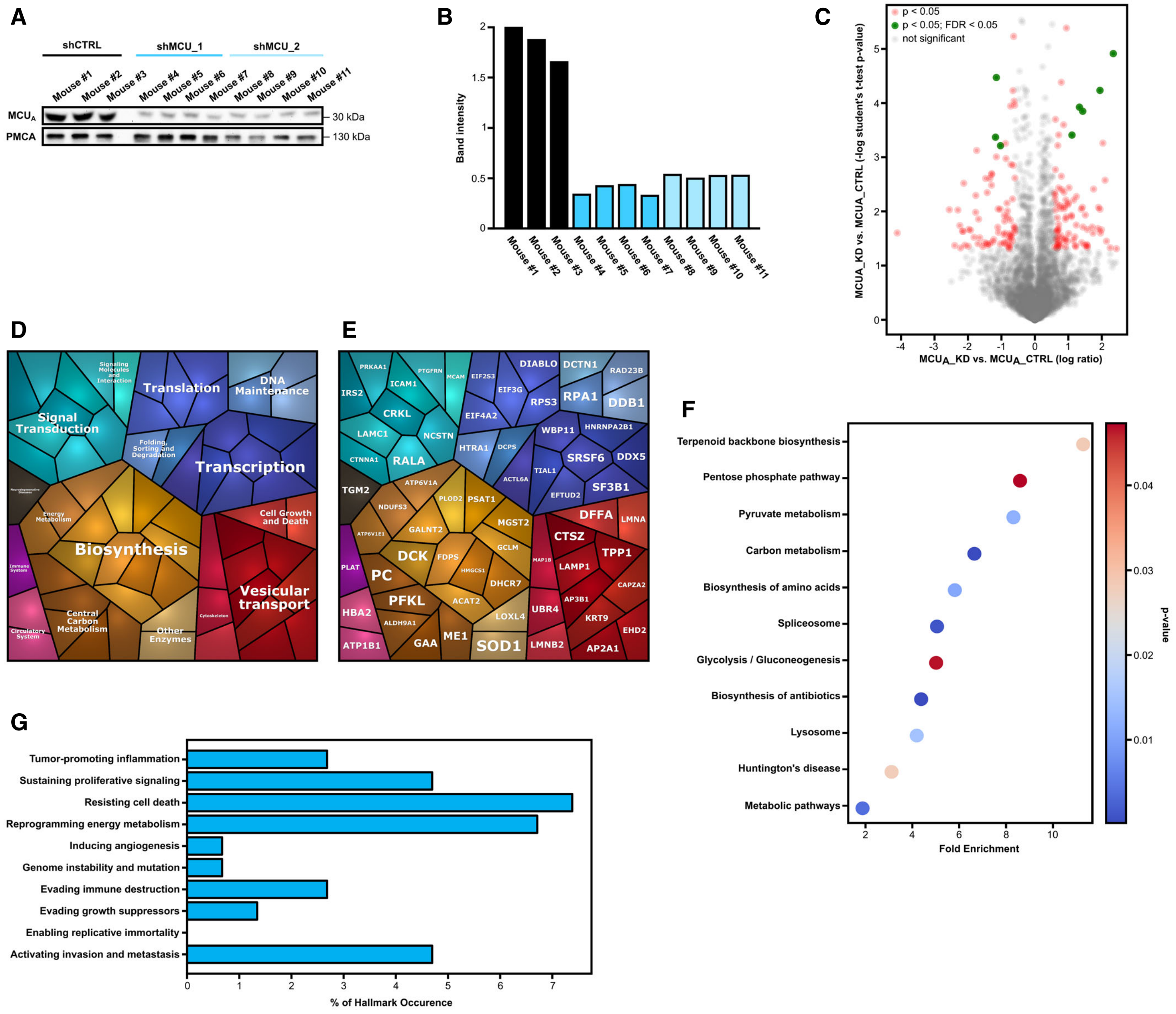

Figure EV3. MCU_A controls metabolic signaling in 1205Lu melanoma cells (related to Figs 3 and 4).

A, B Validation of MCU_A knockdown in melanoma xenografts. WB for MCU_A in 1205Lu MCU_{A_KD} cells isolated from xenograft tumors (A). Corresponding band intensity, shown as the ratio between MCU_A and the loading control plasma membrane Ca^{2+} ATPase (PMCA) (B).

C Volcano plot of proteomics data for 1205Lu MCU_{A_KD} cells versus MCU_A_CTRL. Protein entities with significant *P*-values ($P < 0.05$) are marked with red dots. Protein entities with significance in both *P*-value and FDR (false discovery rate) ($P < 0.05$; FDR < 0.05) are marked with green dots.

D, E Proteomap analyses with protein entities that display significant difference (absolute Log2 fold change > 0.5 and $P < 0.05$) between 1205Lu MCU_{A_KD} and 1205Lu shCTRL cells.

F KEGG-based analysis of cellular components and processes based on protein hits that show differential expression between in 1205Lu MCU_{A_KD} and shCTRL cells, revealed via proteomics.

G Cancer hallmark-based enrichment analysis of proteins differentially expressed in 1205Lu MCU_{A_KD} versus shCTRL cells, based on proteomics data shown in (A). Percentage of hits in the specific gene set is displayed.

Source data are available online for this figure.

 © 2022 The Authors

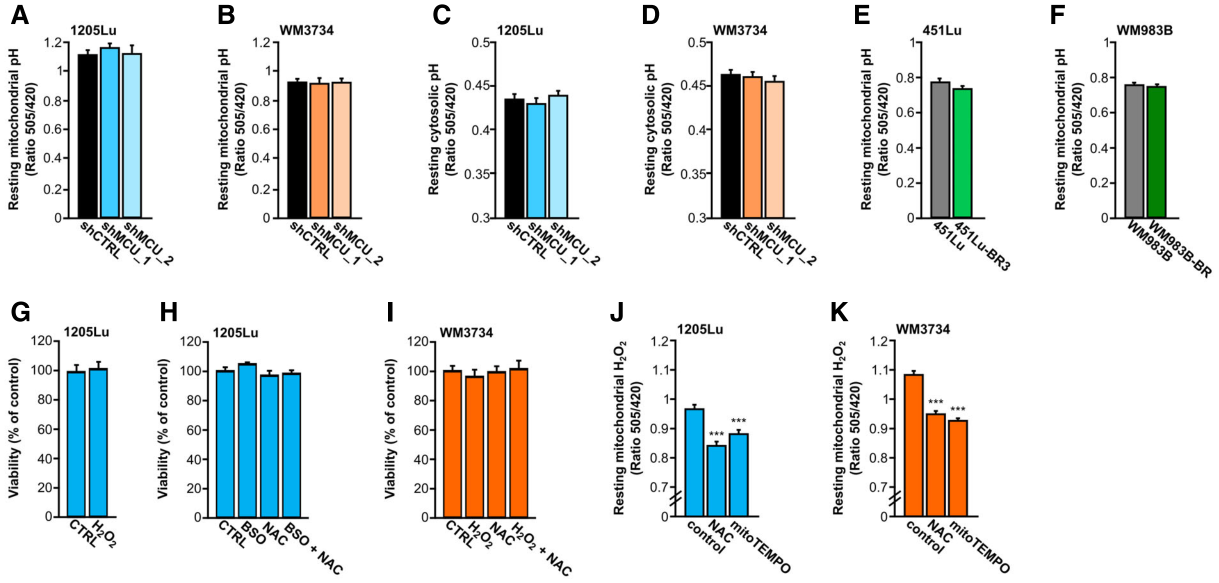

Figure EV4. Effect of redox agents on melanoma cell viability and mitochondrial ROS (related to Fig 5).

A, B Quantification of basal mito-SypHer ratios (pH control for mito-HyPer) in 1205Lu (A) and WM3734 (B) with and without stable MCU_{A_KD} (1205Lu—shCTRL: *n* = 210 cells from six biological replicates; shMCU_1: *n* = 174 cells from six biological replicates; shMCU_2: *n* = 117 cells from six biological replicates; WM3734—shCTRL: *n* = 74 from three biological replicates; shMCU_1: *n* = 51 cells from three biological replicates; shMCU_2: *n* = 67 cells from three biological replicates).

C, D Quantification of basal SypHer ratios (pH control for HyPer) in 1205Lu (C) and WM3734 (D) with and without stable MCU_{A_KD} (1205Lu—shCTRL: *n* = 149 cells from four biological replicates; shMCU_1: *n* = 176 cells from four biological replicates; shMCU_2: *n* = 158 cells from four biological replicates; WM3734—shCTRL: *n* = 187 cells from six biological replicates; shMCU_1: *n* = 189 cells from six biological replicates; shMCU_2: *n* = 193 cells from six biological replicates).

E, F Quantification of basal mito-SypHer ratios (pH control for mito-HyPer) in 451Lu (E) and WM983B (F) and their respective BRAF inhibitor-resistant versions (451Lu: *n* = 73 cells from four biological replicates; 451Lu-BR3: *n* = 136 cells from four biological replicates; WM983B: *n* = 103 cells from four biological replicates; WM983B-BR: *n* = 91 cells from four biological replicates).

G Viability assay upon 100 µM H_2O_2 treatment for 24 h in 1205Lu melanoma cells (*n* = 4 biological replicates).

H Viability assay upon 4-h treatment with 1 mM BSO, 200 µM NAC and 1 mM BSO + 200 µM NAC in 1205Lu melanoma cells (*n* = 12 wells from three biological replicates).

I Viability assay upon 4-h treatment with 100 µM H_2O_2, 200 µM NAC and 100 µM H_2O_2 + 200 µM NAC in WM3734 melanoma cells (*n* = 12 wells from three biological replicates).

J, K Resting mitochondrial hydrogen peroxide (H_2O_2; mito-HyPer) in 1205Lu (J) and WM3734 (K) with and without overnight pre-treatment with NAC (200 µM) or mito-TEMPO (1 µM) (1205Lu—control: *n* = 191 cells from 3 biological replicates; NAC: *n* = 148 cells from three biological replicates; mitoTEMPO: *n* = 133 cells from three biological replicates; WM3734—control: *n* = 237 cells from four biological replicates; NAC: *n* = 263 cells from five biological replicates; mitoTEMPO: *n* = 255 cells from three biological replicates).

Data information: (A–F) and (J–K) were measured in Ringer's buffer containing 0.5 mM Ca^{2+}. Data are presented as mean ± SEM. Statistical significance was determined using unpaired, two-tailed Student's *t*-test, ***$P < 0.005$; no asterisk means no statistical significance ($P > 0.05$).

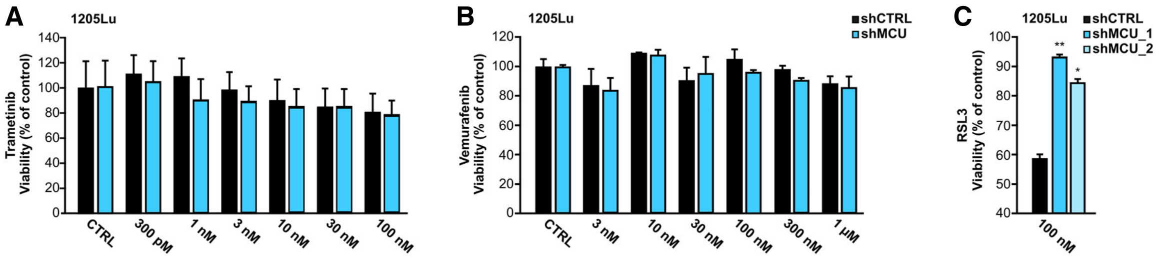

Figure EV5. MCU_A controls melanoma cell therapeutic sensitivity (related to Fig 7).

A, B 1205Lu shCTRL and MCU_{A_KD} cell viability following incubation with different concentrations of trametinib for 144 h (A) and vemurafenib for 96 h (B). Data are shown as percent of control (untreated cells = CTRL) (n = 3 biological replicates).

C 1205Lu cell viability upon incubation with 100 nM RSL3 for 72 h. Data are shown as percent of control (untreated cells, not shown) ($n \leq 8$ biological replicates). RSL, RAS-selective lethal.

Data information: Data are presented as mean ± SEM. Statistical significance was determined using unpaired, two-tailed Student's t-test, $*P < 0.05$; $**P < 0.01$; no asterisk means no statistical significance ($P > 0.05$).

 © 2022 The Authors
