# Supplementary material for: MCU controls melanoma progression through a redox‐controlled phenotype switch
Source: EMBO Rep. 2022 Sep 26;23(11):e54746. doi: 10.15252/embr.202254746 (PMC9638851; doi:10.15252/embr.202254746)
Supplement: Supplementary file 13 [file EMBR-23-e54746-s002.pdf]

EMBO reports

MCU controls melanoma progression through a redox-controlled phenotype switch

Ioana Stejerean-Todoran, Katharina Zimmermann, Christine Gibhardt, Adina Vultur, Christian Ickes, Batool Shannan, Zurine Bonilla del Río, Anna Wölling, Sabrina Cappello, Hsu-Min Sung, Magdalena Shumanska, Xin Zhang, Maithily Nanadikar, Muhammad Latif, Anna Wittek, Felix Lange, Andrea Waters, Patricia Brafford, Jörg Wilting, Henning Urlaub, Dörthe Katschinski, Peter Rehling, Christof Lenz, Stefan Jakobs, Volker Ellenrieder, Alexander Roesch, Michael Schön, Meenhard Herlyn, Hedwig Stanisz, and Ivan Bogeski
DOI: 10.15252/embr.202254746

Corresponding author(s): Ivan Bogeski (ivan.bogeski@med.uni-goettingen.de)

Review Timeline:

Submission Date:	28th Jan 22
Editorial Decision:	31st Jan 22
Revision Received:	30th Jun 22
Editorial Decision:	17th Aug 22
Revision Received:	29th Aug 22
Accepted:	29th Aug 22

Editor: Achim Breiling

Transaction Report: This manuscript was transferred to EMBO reports following peer review at The EMBO Journal.

(Note: With the exception of the correction of typographical or spelling errors that could be a source of ambiguity, letters and reports are not edited. Depending on transfer agreements, referee reports obtained elsewhere may or may not be included in this compilation. Referee reports are anonymous unless the Referee chooses to sign their reports.)

1st Editorial Decision 31st Jan 2022

Dear Prof. Bogeski,

Thank you for transferring your manuscript to EMBO reports. I now went through your manuscript, the referee reports from The EMBO Journal (attached again below) and your revision plan (point-by-point response). The referees have raised several concerns and suggestions to improve the manuscript, or to strengthen the data and the conclusions drawn.

EMBO reports emphasizes novel functional over detailed mechanistic insight, but asks for clear physiological relevance of the findings, and strong experimental support of the major conclusions. It will thus be necessary that in a revised manuscript you address all the points questioning the main conclusions of the study, and all technical concerns, or points regarding the experimental design, model systems used, or data presentation. Looking through your p-b-p-response, I think that this addresses well the points raised.

Given the constructive referee comments, we would like to invite you to further revise your manuscript with the understanding that all referee concerns must be addressed in the revised manuscript and in a detailed point-by-point response (as indicated in your revision plan). Acceptance of your manuscript will depend on a positive outcome of a final round of review at EMBO reports and will therefore depend on the completeness of your responses included in the next, final version of the manuscript.

When submitting your revised manuscript, please also carefully review the instructions that follow below.

PLEASE NOTE THAT upon resubmission revised manuscripts are subjected to an initial quality control prior to exposition to re-review. Upon failure in the initial quality control, the manuscripts are sent back to the authors, which may lead to delays. Frequent reasons for such a failure are the lack of the data availability section (please see below) and the presence of statistics based on n=2 (the authors are then asked to present scatter plots or provide more data points).

When submitting your revised manuscript, we will require:

1) a .docx formatted version of the final manuscript text (including legends for main figures, EV figures and tables), but without the figures included. Please make sure that changes are highlighted to be clearly visible. Figure legends should be compiled at the end of the manuscript text.

2) individual production quality figure files as .eps, .tif, .jpg (one file per figure), of main figures and EV figures. Please upload these as separate, individual files upon re-submission.

The Expanded View format, which will be displayed in the main HTML of the paper in a collapsible format, has replaced the Supplementary information. You can submit up to 5 images as Expanded View. Please follow the nomenclature Figure EV1, Figure EV2 etc. The figure legend for these should be included in the main manuscript document file in a section called Expanded View Figure Legends after the main Figure Legends section. Additional Supplementary material should be supplied as a single pdf file labeled Appendix. The Appendix should have page numbers and needs to include a table of content on the first page (with page numbers) and legends for all content. Please follow the nomenclature Appendix Figure Sx, Appendix Table Sx etc. throughout the text, and also label the figures and tables according to this nomenclature.

For more details please refer to our guide to authors:
http://www.embopress.org/page/journal/14693178/authorguide#manuscriptpreparation

See our guide for figure preparation:
http://wol-prod-cdn.literatumonline.com/pb-assets/embo-site/EMBOPress_Figure_Guidelines_061115-1561436025777.pdf

See also the guidelines for figure legend preparation:
https://www.embopress.org/page/journal/14693178/authorguide#figureformat

3) a .docx formatted letter INCLUDING the reviewers' reports and your detailed point-by-point responses to their comments. As part of the EMBO Press transparent editorial process, the point-by-point response is part of the Review Process File (RPF), which will be published alongside your paper.

4) a complete author checklist, which you can download from our author guidelines (https://www.embopress.org/page/journal/14693178/authorguide). Please insert page numbers in the checklist to indicate where the requested information can be found in the manuscript. The completed author checklist will also be part of the RPF.

Please also follow our guidelines for the use of living organisms, and the respective reporting guidelines:
http://www.embopress.org/page/journal/14693178/authorguide#livingorganisms

5) that primary datasets produced in this study (e.g. RNA-seq, ChIP-seq and array data) are deposited in an appropriate public database. This is now mandatory (like the COI statement). If no primary datasets have been deposited in any database, please state this in this section (e.g. 'No primary datasets have been generated and deposited').

See also: http://embor.embopress.org/authorguide#datadeposition

Please remember to provide a reviewer password if the datasets are not yet public.

The accession numbers and database should be listed in a formal "Data Availability " section (placed after Materials & Methods) that follows the model below. Please note that the Data Availability Section is restricted to new primary data that are part of this study.

Data availability

The datasets produced in this study are available in the following databases:

- RNA-Seq data: Gene Expression Omnibus GSE46843 (https://www.ncbi.nlm.nih.gov/geo/query/acc.cgi?acc=GSE46843)
- [data type]: [name of the resource] [accession number/identifier/doi] ([URL or identifiers.org/DATABASE:ACCESSION])

*** Note - All links should resolve to a page where the data can be accessed. ***

Moreover, I have these editorial requests:

6) We strongly encourage the publication of original source data with the aim of making primary data more accessible and transparent to the reader. The source data will be published in a separate source data file online along with the accepted manuscript and will be linked to the relevant figure. If you would like to use this opportunity, please submit the source data (for example scans of entire gels or blots, data points of graphs in an excel sheet, additional images, etc.) of your key experiments together with the revised manuscript. If you want to provide source data, please include size markers for scans of entire gels, label the scans with figure and panel number, and send one PDF file per figure.

7) Our journal encourages inclusion of *data citations in the reference list* to directly cite datasets that were re-used and obtained from public databases. Data citations in the article text are distinct from normal bibliographical citations and should directly link to the database records from which the data can be accessed. In the main text, data citations are formatted as follows: "Data ref: Smith et al, 2001" or "Data ref: NCBI Sequence Read Archive PRJNA342805, 2017". In the Reference list, data citations must be labeled with "[DATASET]". A data reference must provide the database name, accession number/identifiers and a resolvable link to the landing page from which the data can be accessed at the end of the reference. Further instructions are available at: http://www.embopress.org/page/journal/14693178/authorguide#referencesformat

8) Regarding data quantification and statistics, please specify, where applicable, the number "n" for how many independent experiments (biological or technical replicates) were performed, the bars and error bars (e.g. SEM, SD) and the test used to calculate p-values in the respective figure legends. Please provide statistical testing where applicable, and also add a paragraph detailing this to the methods section. See:
http://www.embopress.org/page/journal/14693178/authorguide#statisticalanalysis

9) Please also note our reference format:
http://www.embopress.org/page/journal/14693178/authorguide#referencesformat

10) For microscopic images, please add scale bars of similar style and thickness to all the microscopic images, using clearly visible black or white bars (depending on the background). Please place these in the lower right corner of the images. Please do not write on or near the bars in the image but define the size in the respective figure legend.

11) We updated our journal's competing interests policy in January 2022 and request authors to consider both actual and perceived competing interests. Please review the policy https://www.embopress.org/competing-interests and update your competing interests if necessary. Please name this section 'Disclosure and Competing Interests Statement'.

12) Please provide the abstract written in present tense (and with not more than 175 words) and order the manuscript sections like this (using these names):
Title page - Abstract - Introduction - Results - Discussion - Materials and Methods -Data availability section - Acknowledgements - Author contributions - Disclosure and Competing Interests Statement - References - Figure legends - Expanded View Figure legends.

I look forward to seeing a revised version of your manuscript when it is ready. Please let me know if you have questions or comments regarding the revision.

Please use this link to submit your revision: https://embor.msubmit.net/cgi-bin/main.plex

Best,

Achim Breiling

Achim Breiling
Senior Editor
EMBO Reports

Referee #1:

This manuscript addressed the role of the mitochondrial Ca2+ influx channel MCU in melanoma metastasis. The authors first used multiple databases to analyze a potential connection between MCU and melanoma patients and MCU and other cancers and found reduced survival with low expression of MCU. This led them to explore the role of MCU in melanocytes proliferation and metastasis in model system and mice. They concluded that the reduced expression of MCU cased a change in the mitochondrial and cellular redox potential and thus increased metastasis and reduced growth. Finally, the authors showed that melanoma cell likes with reduced MCU activity survive much better killing by human NK cells.

Although the topic is of interest and the results in Figure 6 on survival of melanoma cells killing by NK cells are highly significant, the analysis of the role of MCU in melanoma cells and the connection between MCU level and metastasis are not well established.

Specific comments:
1. Figure 1: As impressive as the analysis of MCU and survival of melanoma patient, there is no evidence in melanoma cells that MCU is actually reduced. The results in Figure 1H and 1I show no change in MCU level. This, considerably, weaken the conclusion that reduced MCU is the cause of poor survival (see also below).

2. Figure 1: To exclude changes in other proteins of the MCU complex, higher number of melanoma cells need to be analyzed and protein levels, not only mRNA, should be determined.

3. Important missing experiments are analyzing mitochondria Ca2+ homeostasis and redox or H2O2 in melanoma cells without artificial manipulation of MCU by knockout/down to see if they are different

4. Figure 2, related to comment 1: the role of MCU is tested by knockout or knockdown in two melanoma cell lines. To establish a role of MCU in metastasis the authors need to use a more physiological mode of increasing metastasis and tumorigenesis in these cells, such UV radiation or other procedures, to show that this caused reduction in MCU expression and then correlate this with cell proliferation, metastasis in cell system, and in mice.

5. Figure 2: The use of thapsigargin to assay the effect of mitochondria Ca2+ is not specific enough . These experiments should be done with a more physiological stimulus that causes release from ER by the IP3 receptors that normally feed MCU and mitochondrial Ca2+ uptake. Similar analysis of control and melanoma cells made metastatic should be performed to establish a strong correlation between MCU and metastasis.

6. In all experiments the authors used melanoma cells in which MCU level was reduced by knockdown/out. What are the controls for these experiments? How does knockout of MCU in a non-melanoma cell like affect their functions, at least in all cellular assay used in the present studies?

7. Figure 4: The proteome analysis as it stands is not sufficiently informative. Validation in the role of the proteome and of the hits, at least some of the top and novel hits, should be examined in one or two of the cell assays of proliferation and metastasis.

8. Figure 5: The authors attribute metastasis potential to the state of H2O2 and redox potential in melanoma cells. Does a change in redox potential has the same effect as knockout of MCU and do the cells with MCU knocked out resistant to the drugs used to change the mitochondrial redox potential.

Referee #2:

In this study, Zimmerman et al. have examined the role of MCU (or mitochondrial Ca2+ uptake) on the proliferation and invasive behavior of melanoma cells. The MCU complex is a major route for calcium into the mitochondrial matrix but if and how MCU

affects melanoma pathobiology is not understood. Based on the findings the authors suggest that MCUA expression strongly correlates with melanoma patient survival. Knockdown (KD) of MCUA suppressed melanoma cell growth but promoted migration and invasion in 2D and 3D cultures. In melanoma xenografts, MCUA_KD reduced tumor volumes but promoted lung metastases. Proteomic analyses and protein microarrays identified pathways that link MCUA abundance and melanoma cell phenotype and suggested a major role for metabolic and redox regulation. Antioxidants enhanced melanoma cell migration, while pro-oxidants diminished the MCUA_KD induced invasive phenotype. Furthermore, MCUA_KD increased melanoma cell resistance to immunotherapies and ferroptosis.

This is a very interesting and thorough study and can potentially provide new therapeutic strategies. While the experiments have been carefully done and data provided are very clear, the link between mitochondria and how it differentially regulates increase or suppression of proliferation/ invasion is not established. Is it related to mitochondrial function ? Are changes in ATP generation or energetics involved? No data are presented to show the status of mitochondrial function (ATP, respiration, membrane potential) under various conditions or how increase in ROS affects mitochondrial function (membrane potential). Since mitochondrial Ca2+ uptake is known to increase mitochondrial respiration and ATP synthesis, it is important to assess mitochondrial function directly. Further, since certain conditions cause increased cell death, there must be a collapse of mitochondrial membrane potential. This is an important aspect that needs to be examined to substantiate the findings.

Specific points:
1. What is the status of mitochondrial function and energetics in cells that are either proliferative or displaying increased invasiveness? Are there specific contributions of mitochondrial energetics to these phenotypes.
2. How does mitochondrial ROS affect mitochondrial function ?
3. It might be useful to test the effects of specific mitochondrial and cytosolic ROS scavenger.
4. Are other ROS-activated channels, such as TRPM2, involved ? This channel has also been previously shown to mediate the effects of ROS-generating chemotherapeutics. So it is important to examine the function of this channel.

Referee #3:

In this study, Zimmermann et al studied the contribution of the mitochondrial Ca2+ uniporter (MCU or MCUa) to melanoma progression. Using TCGA, they report that melanoma patients with low expression of MCU have worse patient survival. The authors then generated two sets of shRNA-mediated Knockdown (KD) cells of MCU and showed that MCU knockdown suppresses melanoma growth but enhances migration and invasion. In vivo melanoma xenografts studies also confirmed that cells with MCU KD generated smaller tumors while promoting metastasis of these cells to the lung. In order to shed some light on the mechanisms of this dual regulation by MCU of melanoma growth and invasions, the authors performed protein microarrays on control and MCU-KD cells to identify pathways by which MCU controls melanoma cell phenotype. They honed on redox regulation as a potential mechanism and confirmed the contribution of ROS using a series of oxidant and antioxidant experiments performed in vitro where they showed that antioxidants enhanced melanoma migration, while pro-oxidants inhibited invasion of MCU-KD cells. Finally, pathway analysis also revealed that MCU-KD melanoma cells have increased resistance to immunotherapies and ferroptosis. This is an interesting study that appears to be carefully conducted. While the subject of Ca2+ and ROS crosstalk in cancer is a highly significant area of research which remains incompletely understood, the findings of the current study are somewhat preliminary. The fact that the different functions controlled by ROS are context-dependent and that different levels of ROS can have opposing results in cellular physiology and pathophysiology are not novel. The role of mitochondrial Ca2+ and ROS and the molecular players involved in melanoma has been described in detail in Zhang et al. 2019. The current study (using the authors own words) focused on the pathways and not the individual proteins and ended up on the descriptive side without offering one major in-depth and thorough mechanism of how MCU function connects to ferroptosis and resistance to immunotherapies. This manuscript can become a highly significant study if the authors can provide such detailed mechanistic insights making those connections. This and other comments are listed below.

Major comments:
1. In Figure 2, to test for invasiveness, the transwell should have a Matrigel coating. Then only the experiment will say anything about invasiveness. Unless this was done in the Matrigel-coated chambers, the experiments should be repeated. If indeed it was done in Matrigel-coated transwells, please mention this in the methods/results/legends.

2. In Figure S2, The MCU protein levels in WM3734 are significantly different between D and H. I know the blots are different, but the cell lines are the same. In Figure S2D, 1206LU shcontrol and WM3734 control have the same MCU protein levels, but in Figure S2H, WM3734 has 50% less MCU. Please explain.

3. In Figure 3, given that the cell lines and knockdown condition are giving variable results in proliferation assays, transwell migration assays and spheroid size analysis should be done in both the cell lines and two different shRNA sequences.

4. In Figure 5, Please perform all these crucial experiments in both cell lines with two different shRNA. Such as measurements of both mitochondrial and cytoplasmic ROS.

5. In Figure 5, The ROS levels in cytoplasm and mitochondria should be determined after the antioxidant treatment.

6. In Figure 5, Reduced MCU function leads to increased mitochondrial and cytosolic ROS. Using a generic antioxidant does not distinguish if the phenotype is due to mtROS of cytoROS. Rescue experiments, migration, and invasion should also be performed using mitoTEMPO. This will determine the role of mitoROS in the phenotype.

7. In Figure 5, The viability after drug treatment should be performed in both cell lines.

8. There is no mechanism suggested to explain why reduced mtCa2+ is causing reduced overall ROS in cells. How lower ROS level is causing increased migration and invasion is not determined. How the ROS levels affect the therapeutic sensitivity and metastatic spread is also not clear. In Figures 4 and 6, there are multiple pathways and proteins that are altered due to the reduced function of MCU, which can explain the phenotype. But none of the pathways are validated. Furthermore, mitochondrial structure and function should be determined in both the cell lines and knockdown conditions. That might also help explain the reduced ROS phenotype. Without some of these insights, the scheme in figure 7 is not supported by data, and the manuscript remains preliminary.

9. All bar graphs should have individual data points. This will help in determining the data distribution and will also self-explain the number of repeats.

Minor Comments:
1. In Figures 2 D and E, please explain why the mtCa2+ influx is only ~40% reduced in WM3734 shMCU even when the MCU protein levels are 95% reduced.

2. In Figure 4H, please write what is the scale bar of the heat map represents and why there is no value for control cells.

3. In Figure 3G, It is not clear how the invasion of spheroids was measured. The invasion should be measured in 1205LU cells also.

4. Please fix the sentence "Cells were injected with the MCUA_KD cells and their" in the melanoma xenograft, under methods on page 22.

5. Please mention the number of animals used in the main text or figure legends. This issue can also be solved by making graphs with individual data points or scatter blots.

POINT-BY-POINT REPLY

Referee #1:

This manuscript addressed the role of the mitochondrial Ca2+ influx channel MCU in melanoma metastasis. The authors first used multiple databases to analyze a potential connection between MCU and melanoma patients and MCU and other cancers and found reduced survival with low expression of MCU. This led them to explore the role of MCU in melanocytes proliferation and metastasis in model system and mice. They concluded that the reduced expression of MCU cased a change in the mitochondrial and cellular redox potential and thus increased metastasis and reduced growth. Finally, the authors showed that melanoma cell likes with reduced MCU activity survive much better killing by human NK cells.

Although the topic is of interest and the results in Figure 6 on survival of melanoma cells killing by NK cells are highly significant, the analysis of the role of MCU in melanoma cells and the connection between MCU level and metastasis are not well established.

We thank the reviewer for acknowledging the potential interest of this study. We hope that the revised version and the new experimental data will provide an improved understanding about the role of MCU on melanoma cell aggressive behavior.

Specific comments:

1. Figure 1: As impressive as the analysis of MCU and survival of melanoma patient, there is no evidence in melanoma cells that MCU is actually reduced. The results in Figure 1H and 1I show no change in MCU level. This, considerably, weaken the conclusion that reduced MCU is the cause of poor survival (see also below).

2. Figure 1: To exclude changes in other proteins of the MCU complex, higher number of melanoma cells need to be analyzed and protein levels, not only mRNA, should be determined.

We understand the reviewer's concern and are thankful for her/his suggestions.

The Kaplan Meier-based evaluation of patient datasets clearly shows that patients with low expression of MCU_A have reduced survival. Our further evaluation of these datasets also indicated that parameters such as mutational status, disease stage, patient age or gender do not influence MCU_A expression. The results presented in the former Figure 1H-I confirmed these findings. We now realize that these data can be misleading and removed the two panels from Figure 1.

Nevertheless, we agree that additional analyses of the MCU_A expression in melanoma cell lines could provide a deeper understanding of the correlation between MCU_A and melanoma invasiveness. Given that categorizing cell lines in aggressive versus less aggressive is not trivial, we now used an experimental system in which we compared melanoma cells resistant to targeted therapies/small molecules such as the BRAF kinase inhibitors. These lines are known to be highly aggressive as compared to their control counterparts. As seen in our New Figure 1H-I, the expression of MCU_A in BRAF inhibitor-resistant 451Lu-BR3 and WM983B-BR melanoma cells was diminished compared to the control cell lines. In addition, as requested by the reviewer, the other components of the MCU complex were also evaluated.

We believe that these new findings strengthen the link between MCU_A expression and disease severity. Moreover, they support the observations from melanoma patient datasets.

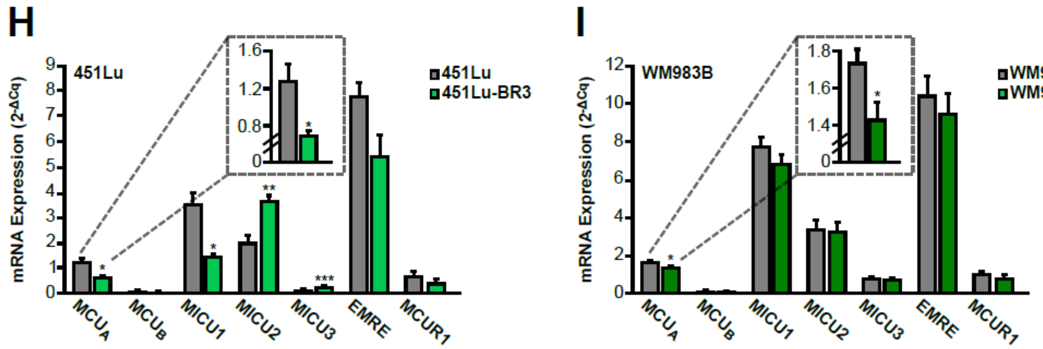

New Figure 1H-I. mRNA expression of MCU complex components (MCU_A, MCU_B, MICU1, MICU2, MICU3, EMRE, MCUR1) in 451Lu (H) and WM983B (I) melanoma cells with and without resistance to BRAF inhibitors, normalized to the housekeeping gene TBP, quantified by RT–qPCR (n≥3). Data are presented as mean ± SEM. Statistical significance was assessed using unpaired, two-tailed Student's t-test, (*) $p < 0.05$; (**) $p < 0.01$; (***) $p < 0.005$.

3. Important missing experiments are analyzing mitochondria Ca2+ homeostasis and redox or H2O2 in melanoma cells without artificial manipulation of MCU by knockout/down to see if they are different

We agree that such analysis is important. We thus measured mitochondrial Ca^{2+} and H2O2 in the BRAF inhibitor-resistant cells and their controls in which MCU_A expression was not artificially manipulated. Our findings indicate that the mitochondrial Ca^{2+} uptake was decreased in the BRAF inhibitor-resistant cells versus their counterparts (New Figure 2Q-V). Of note these cells also displayed a diminished MCU_A expression (New Figure 1H-I).

Hyper measurements showed diminished mitochondrial H_2O_2 production in the 451Lu-BR3 and the WM983B-BR cells thus supporting the hypothesis that MCU_A i.e. mitochondrial Ca^{2+} are important determinants of the mitochondrial redox status (New Figure 5L-M).

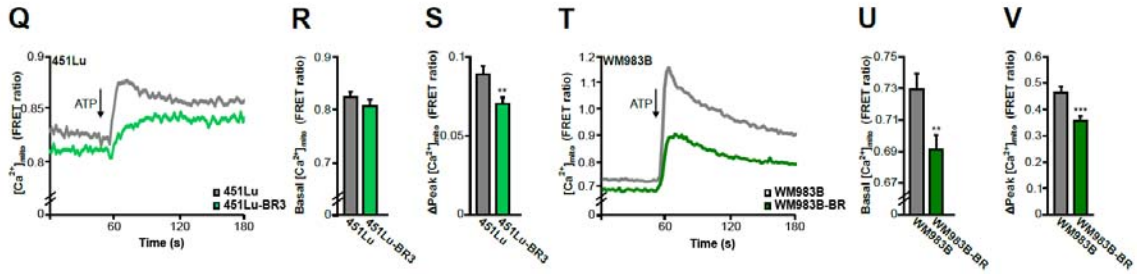

New Figure 2. (Q-S) Mitochondrial calcium uptake (represented as FRET ratio) in 451Lu (grey; n=157) and BRAF inhibitor-resistant 451Lu (451Lu-BR3; green; n=164) upon physiological stimulation with ATP (100 µM). Quantification of basal levels (R) and Ca^{2+} uptake (Δpeak) (S). (T-V) Mitochondrial calcium uptake (represented as FRET ratio) in WM983B (grey; n=151) and BRAF inhibitor-resistant WM983B (WM983B-BR; green; n=133) upon physiological stimulation with ATP (100 µM). Quantification of basal levels (U) and Ca^{2+} uptake (Δpeak) (V). Data were measured in Ringer's buffer containing 0.5 mM Ca^{2+} and are presented as mean ± SEM. Statistical significance was determined using unpaired, two-tailed Student's t-test, (*) $p < 0.05$; (**) $p < 0.01$; (***) $p < 0.005$.

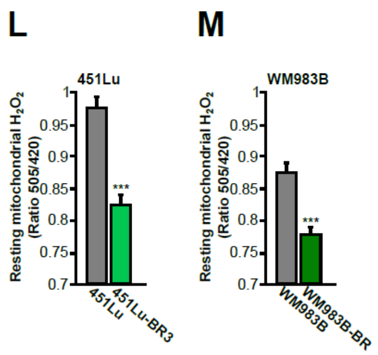

New Figure 5L-M. Resting mitochondrial hydrogen peroxide (H_2O_2) in 451Lu and BRAF inhibitor-resistant 451Lu-BR3 (L) and WM983B and BRAF inhibitor-resistant WM983B-BR (M) (451Lu=227; 451Lu-BR=166; WM983B=193; WM983B-BR=208). Data were measured in Ringer's buffer containing 0.5 mM Ca^{2+} and statistical significance was determined using unpaired, two-tailed Student's t-test, (*) $p < 0.05$; (**) $p < 0.01$; (***) $p < 0.005$.

4. Figure 2, related to comment 1: the role of MCU is tested by knockout or knockdown in two melanoma cell lines. To establish a role of MCU in metastasis the authors need to use a more physiological mode of increasing metastasis and tumorigenesis in these cells, such UV radiation or other procedures, to show that this caused reduction in MCU expression and then correlate this with cell proliferation, metastasis in cell system, and in mice.

We would like to point out that our bioinformatic data in Fig. 1 and Fig. S1 indicate that no relevant melanoma-relevant driver mutations i.e. genetic factors are involved in the regulation of MCU_A expression. Our additional bioinformatic analyses (shown below) support these conclusions as only 1 out of 1338 patients with cutaneous melanoma displayed a MCU_A deletion. Moreover, in only 17 patients of the same cohort MCU_A mutations (whose effect on MCU_A function is not well understood) were identified (please see Fig. R1 below). Accordingly, we do not believe that MCU_A is involved in melanomagenesis. Our findings rather suggest that MCU_A determines melanoma cell biology following their malignant transformation.

Regarding the environmental or therapy-related factors such as drug resistance, our new findings demonstrate that acquired resistance to BRAF inhibitors causes decreased MCU_A expression and a more aggressive melanoma cell behavior. This was determined by measuring 2D transwell migration and 3D spheroid invasion. As depicted in the new Figures 3K-O, both migration and invasion were significantly increased in the BRAF inhibitor-resistant lines, which exhibit lower MCU_A levels and a decreased mitochondrial Ca^{2+}.

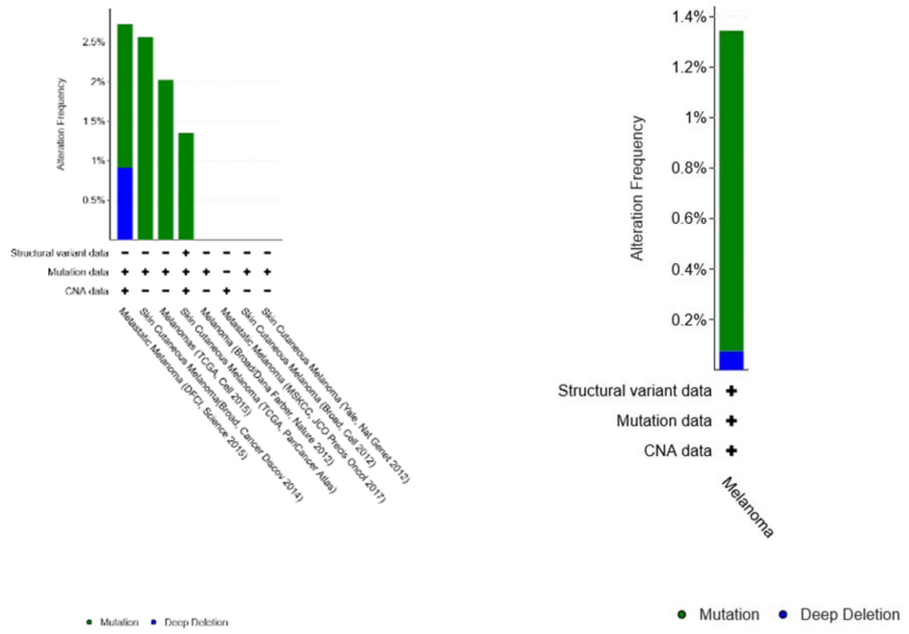

Figure R1. Evaluation of MCUa genetic alteration in melanoma patients (TCGA database)

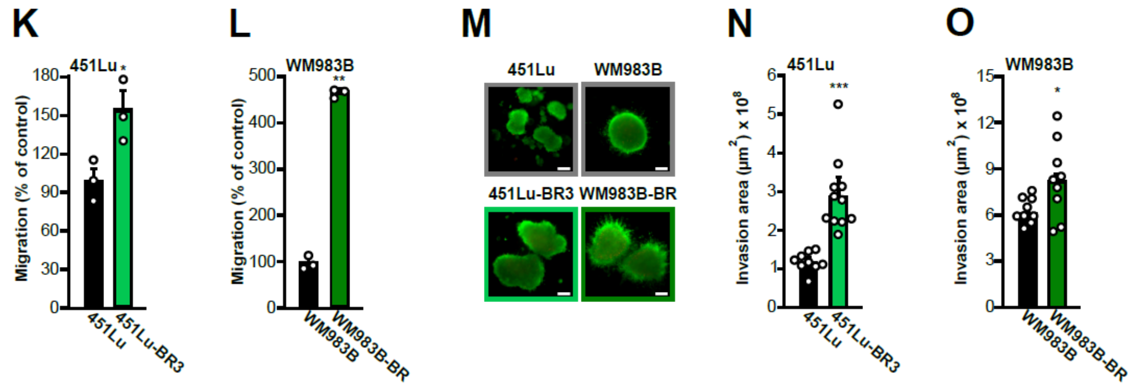

New Figure 3K-O. Migration potential of 451Lu and 451Lu BRAF inhibitor-resistant (451Lu-BR3) (K) and WM983B and WM983B BRAF inhibitor-resistant (WM983B-BR) lines over 24 h (n=3, shown also by individual data points). (M) Representative images of 451Lu wild-type (grey frame), 451Lu-BR3 (lighter green frame), WM983B wild-type (grey frame) and WM983B-BR (darker green frame) melanoma spheroids after 72 h invasion in collagen. Live cells are shown in green. Scale bar: 100 µm. (N-O) Quantification of 451Lu versus 451Lu-BR3 (N) and WM983B versus WM983B-BR (O) spheroid core size (n≥9, shown also by individual data points). Statistical significance was determined using unpaired, two-tailed Student's t-test, (*) $p < 0.05$; (**) $p < 0.01$; (***) $p < 0.005$.

5. Figure 2: The use of thapsigargin to assay the effect of mitochondria Ca2+ is not specific enough. These experiments should be done with a more physiological stimulus that causes release from ER by the IP3 receptors that normally feed MCU and mitochondrial Ca2+ uptake. Similar analysis of control and melanoma cells made metastatic should be performed to establish a strong correlation between MCU and metastasis.

We agree. As indicated above, we tested various physiological stimuli such as ATP, histamine, and noradrenaline and used SOCE measurements as a readout. Our results (New

Figure 2J) showed that all induce SOCE activation, with ATP being the most potent stimulus. As also shown in our reply to comment 3, ATP also induced mitochondrial Ca^{2+} uptake in other melanoma cell lines.

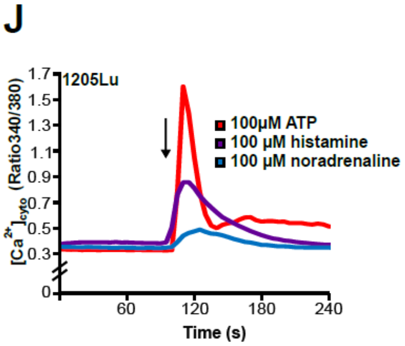

New Figure 2J. Cytosolic Fura-2 AM based Ca^{2+} measurements in 1205Lu cells upon physiological stimulation with ATP (100 µM), histamine (100 µM) and noradrenaline (100 µM) (n≥50).

6. In all experiments the authors used melanoma cells in which MCU level was reduced by knockdown/out. What are the controls for these experiments? How does knockout of MCU in a non-melanoma cell like affect their functions, at least in all cellular assay used in the present studies?

The control cells have been treated with scrambled non-silencing shRNA or siRNA. Is the reviewer referring to healthy melanocytes? If yes, comparing these cells with melanoma cells would be very difficult because of the completely different biology.

7. Figure 4: The proteome analysis as it stands is not sufficiently informative. Validation in the role of the proteome and of the hits, at least some of the top and novel hits, should be examined in one or two of the cell assays of proliferation and metastasis.

We agree that validation of some of the top hits might provide additional understanding of the MCU_A-controlled pathways. In this regard, we want to note that the redox metabolism pathway is one of the top hits and was thus validated. Given that MCU_{A_KD} has a strong impact on cellular metabolism, mitochondrial bioenergetics and other important signaling pathways, evaluating the contribution of single proteins will likely not improve our understanding of the role of MCU_A in melanoma. Hence, we rather focused on additional analyses of the MCU-redox signaling axis. We hope that the reviewer will agree with our approach.

8. Figure 5: The authors attribute metastasis potential to the state of H2O2 and redox potential in melanoma cells. Does a change in redox potential has the same effect as knockout of MCU and do the cells with MCU knocked out resistant to the drugs used to change the mitochondrial redox potential?

Yes. As seen in New Figure 5N-O, treatment with the antioxidant NAC (N-acetylcysteine) enhances cell migration in 1205Lu and WM3734 cells. MCU_{A_KD} (see Fig. 5P) also increases migration when using the same transwell migration assay. As depicted in New Figure 5L-M, the mitochondrial H_2O_2 levels are reduced in the BRAF inhibitor-resistant cell lines.

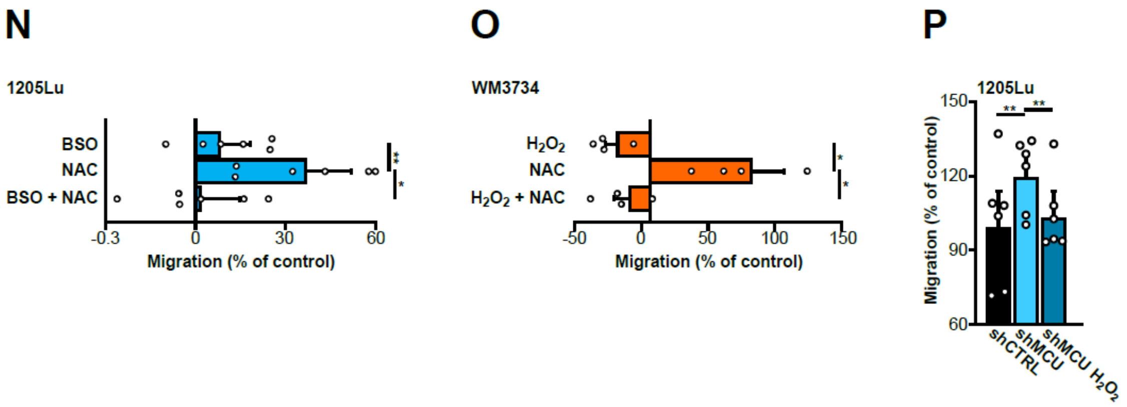

New Figure 5N-P. (N) Migration (4h) of 1205Lu wild-type cells upon 4 h pre-treatment with 1 mM BSO, 200 µM NAC and 1 mM BSO + 200 µM NAC. (O) Migration (4h) of WM3734 wild-type cells upon 4 h pre-treatment with 100 µM H_2O_2, 200 µM NAC and 100 µM H_2O_2 + 200 µM NAC. (P) Migration of 1205Lu shCTRL and shMCU untreated and shMCU pre-treated for 4 h with 100 µM H_2O_2. All data are presented as mean ± SEM. For (N-O) statistical significance was determined using paired, one-tailed Student's t-test, (*) $p < 0.05$; (**) $p < 0.01$; (***) $p < 0.005$. For (P) statistical significance was assessed using paired, two-tailed Student's t-test, (*) $p < 0.05$; (**) $p < 0.01$; (***) $p < 0.005$.

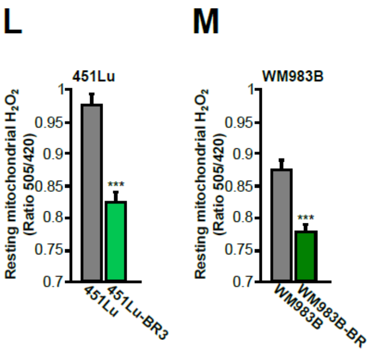

New Figure 5L-M. Resting mitochondrial hydrogen peroxide (H_2O_2) in 451Lu and BRAF inhibitor-resistant 451Lu-BR (L) and WM983B and BRAF inhibitor-resistant WM983B-BR (M) (451Lu=227; 451Lu-BR=166; WM983B=193; WM983B-BR=208). Data were measured in Ringer's buffer containing 0.5 mM Ca^{2+} and statistical significance was determined using unpaired, two-tailed Student's t-test, (*) $p < 0.05$; (**) $p < 0.01$; (***) $p < 0.005$.

Referee #2:

Review: "MCU controls melanoma progression through a redox-controlled phenotype switch" by Zimmermann et al.

In this study, Zimmerman et al. have examined the role of MCU (or mitochondrial Ca2+ uptake) on the proliferation and invasive behavior of melanoma cells. The MCU complex is a major route for calcium into the mitochondrial matrix but if and how MCU affects melanoma pathobiology is not understood. Based on the findings the authors suggest that MCUA expression strongly correlates with melanoma patient survival. Knockdown (KD) of MCUA suppressed melanoma cell growth but promoted migration and invasion in 2D and 3D cultures. In melanoma xenografts, MCUA_KD reduced tumor volumes but promoted lung

metastases. Proteomic analyses and protein microarrays identified pathways that link MCUA abundance and melanoma cell phenotype and suggested a major role for metabolic and redox regulation. Antioxidants enhanced melanoma cell migration, while pro-oxidants diminished the MCUA_KD induced invasive phenotype. Furthermore, MCUA_KD increased melanoma cell resistance to immunotherapies and ferroptosis.

This is a very interesting and thorough study and can potentially provide new therapeutic strategies. While the experiments have been carefully done and data provided are very clear, the link between mitochondria and how it differentially regulates increase or suppression of proliferation/ invasion is not established. Is it related to mitochondrial function? Are changes in ATP generation or energetics involved? No data are presented to show the status of mitochondrial function (ATP, respiration, membrane potential) under various conditions or how increase in ROS affects mitochondrial function (membrane potential). Since mitochondrial Ca2+ uptake is known to increase mitochondrial respiration and ATP synthesis, it is important to assess mitochondrial function directly. Further, since certain conditions cause increased cell death, there must be a collapse of mitochondrial membrane potential. This is an important aspect that needs to be examined to substantiate the findings.

We thank the reviewer for the positive evaluation of our study. We agree that additional mitochondrial parameters were needed in order to better understand the role of MCU_A on mitochondrial function. Our new results suggest that the mitochondrial membrane potential is decreased in all four MCU_{A_KD} cell lines (New Figure 6A-D). Moreover, measurements of ATP using fluorescent dyes and genetically encoded protein sensors showed reduced ATP levels in the MCU_{A_KD} melanoma cell lines (New Figure 6E-I). These new findings thus provide additional insights into the role of MCU_A on mitochondrial function. We hope that these new data answer the questions raised by the reviewer.

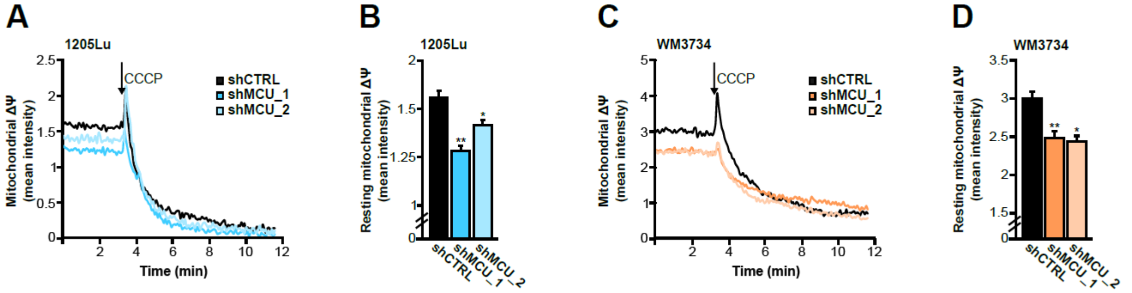

New Figure 6A-D. Resting mitochondrial membrane potential (ΔΨ), measured with TMRE in 1205Lu (A-B) and WM3734 (C-D) with and without stable MCU_{A_KD} (1205Lu: shCTRL=81; shMCU_1=81; shMCU_2=156; WM3734: shCTRL=108; shMCU_1=73; shMCU_2=110). Statistical significance was determined using unpaired, two-tailed Student's t-test, (*) $p < 0.05$; (**) $p < 0.01$; (***) $p < 0.005$.

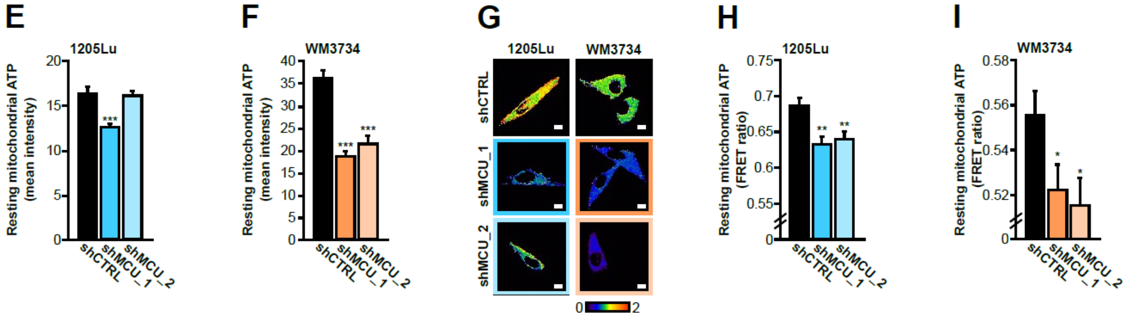

New Figure E-I. (E-F) Resting mitochondrial ATP levels, measured using the ATP-Red dye in 1205Lu (E) and WM3734 (F) with and without stable MCU_{A_KD} (1205Lu: shCTRL=67; shMCU_1=80; shMCU_2=69; WM3734: shCTRL=40; shMCU_1=38; shMCU_2=21). (G-I) Mitochondrial ATP, measured using mito-ATEAM in 1205Lu and WM3734 with and without stable MCU_{A_KD}. Exemplary ratiometric images (FRET/CFP) are shown for all conditions (G). Scale bar: 10 µm. Quantification of basal levels in 1205Lu (H) and WM3734 (I) (1205Lu: shCTRL=141; shMCU_1=143; shMCU_2=143; WM3734: shCTRL=122; shMCU_1=103; shMCU_2=99). Statistical significance was determined using unpaired, two-tailed Student's t-test, (*) $p < 0.05$; (**) $p < 0.01$; (***) $p < 0.005$.

Specific points:

1. What is the status of mitochondrial function and energetics in cells that are either proliferative or displaying increased invasiveness? Are there specific contributions of mitochondrial energetics to these phenotypes?

We agree that examining the influence of mitochondrial bioenergetics on melanoma cell phenotype is important as we already found that mitochondrial respiration correlates with efficacy on melanoma immunotherapies (Cappello et al. 2021, Cancer Research). Hence, we analyzed respiration in a number of melanoma cell lines. Based on the data shown below (Fig. R2), we conclude that the more aggressive metastatic cell lines (red and dark red) have a decreased OCR when compared with the less aggressive melanoma cell lines (green and blue).

Since part of these data has already been published, we did not involve this figure in the revised version of the manuscript.

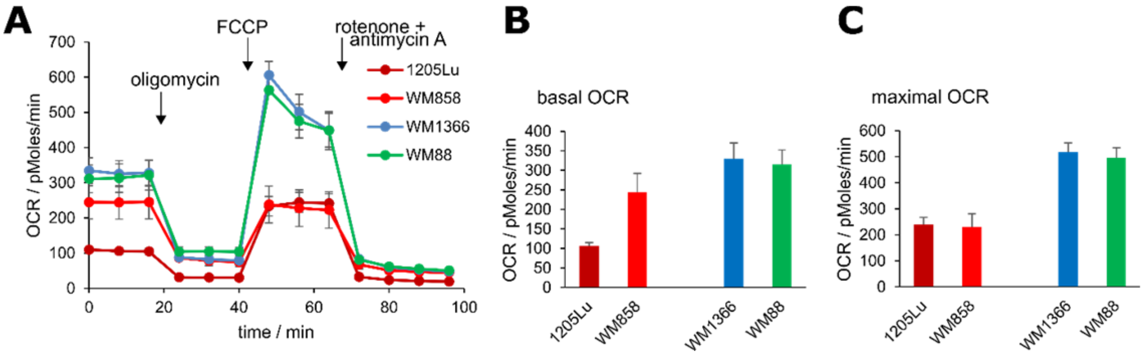

Figure R2. Oxygen consumption rate is dependent on melanoma cell aggressive phenotype (A) Mitochondrial oxygen consumption rate (OCR) of metastatic (red) and non-metastatic (blue-green) melanoma cell lines after injection of oligomycin (3 µM), CCCP (1 µM), antimycin (1 µM) and rotenone (2 µM). (**B+C**) Quantification of basal (**B**) OCR and (**C**) maximal OCR are presented as mean ± SEM ($n > 3$).

2. How does mitochondrial ROS affect mitochondrial function?

This is an important but also a complex question and we believe that the effect of ROS on mitochondria will very much depend on the source of ROS as well as on the mitochondrial compartment (matrix, intramembrane space or the outer mitochondrial membrane) affected. This has already been explored in other studies and if the reviewer agrees, we would not perform additional experiments.

3. It might be useful to test the effects of specific mitochondrial and cytosolic ROS scavenger.

We agree. In addition to the NAC treatment, we now tested the effect of mitochondrially targeted antioxidants such as mitoTEMPO. As seen in Fig. R3, mitoTEMPO was not toxic to

melanoma cells. Moreover, similar as NAC, mitoTEMPO supressed mitochondrial H_2O_2 levels in both cell lines (New Figure S4J-K).

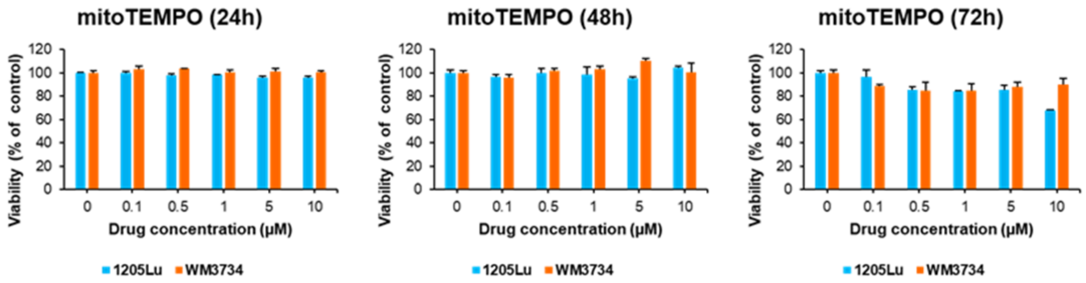

Figure R3. Proliferation of 1205Lu (in blue) and WM3734 (in orange) upon treatment with different concentrations of mitoTEMPO over 72h.

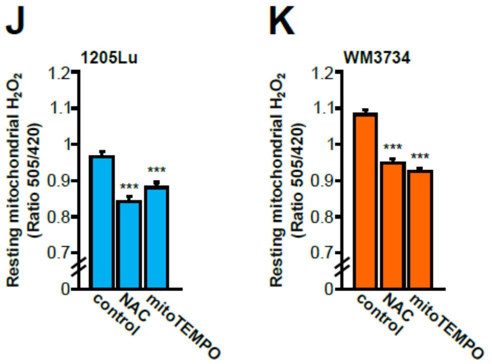

New Figure S4J-K. Resting mitochondrial hydrogen peroxide (H_2O_2) in 1205Lu and WM3734 with and without over-night pre-treatment with NAC (200 µM) or mito-TEMPO (1 µM) (1205Lu: control=191; NAC=148; mito-TEMPO=133; WM3734: control=237; NAC=263; mito-TEMPO=255). Statistical significance was determined using paired, one-tailed Student's t-test, (*) $p < 0.05$; (**) $p < 0.01$; (***) $p < 0.005$.

4. Are other ROS-activated channels, such as TRPM2, involved? This channel has also been previously shown to mediate the effects of ROS-generating chemotherapeutics. So it is important to examine the function of this channel.

We agree that TRPM2 might be very important in the redox regulation of melanoma function and should be studied in this context. Indeed, low TRPM2 expression is linked with decreased patient survival (Fig. R5) in cutaneous melanoma. Nevertheless, MCU_A does not correlate with TRPM2 expression (unpublished data) and we would rather not include these data in the revised manuscript.

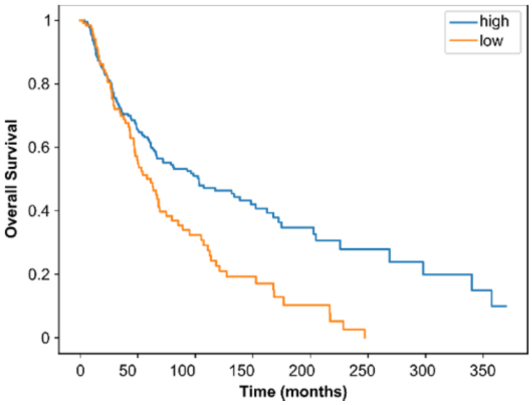

Figure R5. TRPM2 controls melanoma patient survival

Kaplan–Meier survival plots depicting the correlation between TRPM2 mRNA expression levels (high and low) and survival probability of melanoma patients ($p \leq 0.001$)

Referee #3:

In this study, Zimmermann et al studied the contribution of the mitochondrial Ca2+ uniporter (MCU or MCUa) to melanoma progression. Using TCGA, they report that melanoma patients with low expression of MCU have worse patient survival. The authors then generated two sets of shRNA-mediated Knockdown (KD) cells of MCU and showed that MCU knockdown suppresses melanoma growth but enhances migration and invasion. In vivo melanoma xenografts studies also confirmed that cells with MCU KD generated smaller tumors while promoting metastasis of these cells to the lung. In order to shed some light on the mechanisms of this dual regulation by MCU of melanoma growth and invasions, the authors performed protein microarrays on control and MCU-KD cells to identify pathways by which MCU controls melanoma cell phenotype. They honed on redox regulation as a potential mechanism and confirmed the contribution of ROS using a series of oxidant and antioxidant experiments performed in vitro where they showed that antioxidants enhanced melanoma migration, while pro-oxidants inhibited invasion of MCU-KD cells. Finally, pathway analysis also revealed that MCU-KD melanoma cells have increased resistance to immunotherapies and ferroptosis. This is an interesting study that appears to be carefully conducted. While the subject of Ca2+ and ROS crosstalk in cancer is a highly significant area of research which remains incompletely understood, the findings of the current study are somewhat preliminary. The fact that the different functions controlled by ROS are context-dependent and that different levels of ROS can have opposing results in cellular physiology and pathophysiology are not novel. The role of mitochondrial Ca2+ and ROS and the molecular players involved in melanoma has been described in detail in Zhang et al. 2019. The current study (using the authors own words) focused on the pathways and not the individual proteins and ended up on the descriptive side without offering one major in-depth and thorough mechanism of how MCU function connects to ferroptosis and resistance to immunotherapies. This manuscript can become a highly significant study if the authors can provide such detailed mechanistic insights making those connections. This and other comments are listed below:

We thank the reviewer for the positive evaluation regarding the significance of our study and for acknowledging the potential clinical importance of our findings. Indeed, we agree that identifying the mechanism(s) that link melanoma cell therapeutic resistance and MCU could

increase the significance of this study. Accordingly, we performed additional experiments as indicated in our reply to the other reviewers and as described below.

Major comments

1. In Figure 2, to test for invasiveness, the transwell should have a Matrigel coating. Then only the experiment will say anything about invasiveness. Unless this was done in the Matrigel-coated chambers, the experiments should be repeated. If indeed it was done in Matrigel-coated transwells, please mention this in the methods/results/legends.

Thanks for raising this question. We assume the reviewer is referring to the old Fig. 3. The transwell assays were performed without Matrigel coating and when referring to those results we write - migration. We write - invasion when referring to the data obtained from melanoma spheroids. We will clarify this in the revised version of the manuscript and double check that the usage of the both terms is accurate.

2. In Figure S2, The MCU protein levels in WM3734 are significantly different between D and H. I know the blots are different, but the cell lines are the same. In Figure S2D, 1206LU shcontrol and WM3734 control have the same MCU protein levels, but in Figure S2H, WM3734 has 50% less MCU. Please explain.

In Fig. S2D, the stable KD cells (using shRNA) are shown. In S2H on the other hand, we show WM3734 from transiently transfected cells (using siRNA). Comparing signal intensities between blots is not recommended unless the same sample is loaded on all gels, and this was not necessary (and not the goal) for these analyses since the two sets of samples are distinct. We hope the reviewer will accept this explanation.

3. In Figure 3, given that the cell lines and knockdown condition are giving variable results in proliferation assays, transwell migration assays and spheroid size analysis should be done in both the cell lines and two different shRNA sequences.

We agree and now performed additional transwell and spheroid assays (New Figure 3). As shown, MCU_{A_KD} also decreased the spheroid size in WM3734 cells. Unfortunately, the 1205Lu spheroids could not be cultured for 10 days due to the aggressive nature of the cells. To compensate for this, we performed migration and invasion assays in the in the BRAF kinase-resistant cell lines. The results shown in New Figure 3J-N depict increased transwell migration and invasion in the BRAF inhibitors resistant cells. Of note, we also found that these cells have inhibited MCU_A expression and display a suppressed ${}_{mito}Ca^{2+}$ uptake (New Figure 2Q-V).

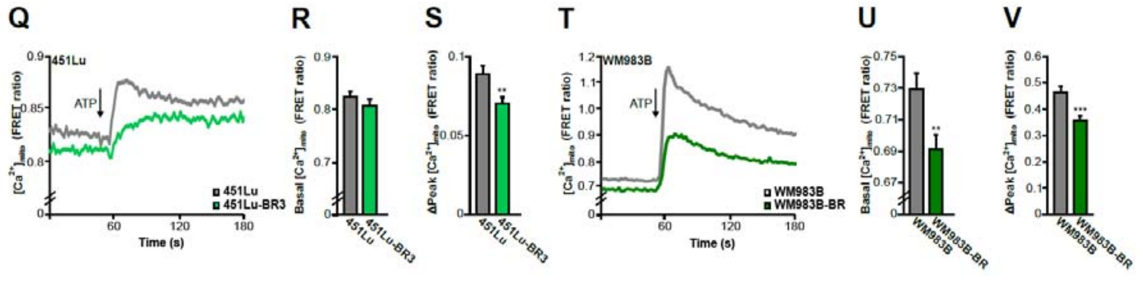

New Figure 2. (Q-S) Mitochondrial calcium uptake (represented as FRET ratio) in 451Lu (grey; n=157) and BRAF inhibitor-resistant 451Lu (451Lu-BR3; green; n=164) upon physiological stimulation with ATP (100 μM). Quantification of basal levels (R) and Ca^{2+} uptake (Δpeak) (S). (T-V) Mitochondrial calcium uptake

(represented as FRET ratio) in WM983B (grey; n=151) and BRAF inhibitor-resistant WM983B (WM983B-BR; green; n=133) upon physiological stimulation with ATP (100 µM). Quantification of basal levels (U) and Ca^{2+} uptake (Δpeak) (V). Data were measured in Ringer's buffer containing 0.5 mM Ca^{2+} and are presented as mean ± SEM. Statistical significance was determined using unpaired, two-tailed Student's t-test, (*) $p < 0.05$; (**) $p < 0.01$; (***) $p < 0.005$.

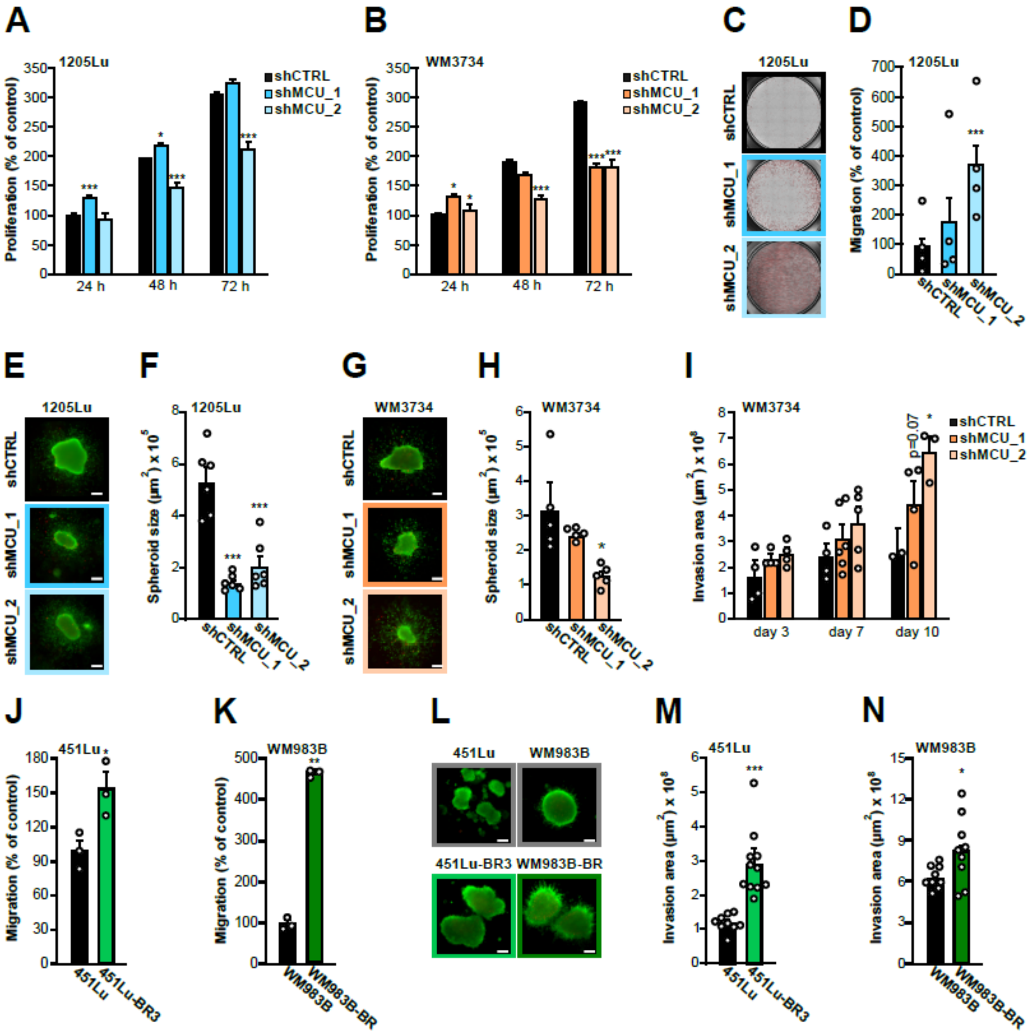

New Figure 3. (A-B) Proliferation of 1205Lu (A) and WM3734 (B) stable MCU KD lines over 72 h, shown as percent of the respective control (shCTRL at 24 h) (n≥7/condition/day). (C) Representative images of the migrated stained 1205Lu shCTRL (black frame), shMCU_1 (darker blue frame) and shMCU_2 (lighter blue frame) cells on the lower side of the insert. (D) Quantification of the transwell migration in 1205Lu stable MCU_{A_KD} lines, based on the number of stained cells (n=4, shown also by individual data points). (E) Representative images of 1205Lu shCTRL (black frame), shMCU_1 (darker blue frame) and shMCU_2 (lighter blue frame) melanoma spheroids after 72 h invasion in collagen. Live cells are shown in green. Scale bar: 100 µm. (F) Quantification of 1205Lu stable MCU_{A_KD} spheroid core size (n=6, shown also by individual data points). (G) Representative images of WM3734 shCTRL (black frame), shMCU_1 (darker orange frame) and shMCU_2 (lighter orange frame) melanoma spheroids. Live cells are shown in green. Scale bar: 100 µm. (H) Quantification of WM3734 stable MCU_{A_KD} spheroid core size (n=5, shown also by individual data points). (I) Invasion potential of WM3734 shCTRL (black), shMCU_1 (darker orange) and shMCU_2 (lighter orange) over a period of 10 days (n≤4/condition/day, shown also by individual data points). (J-K) Migration potential of 451Lu and 451Lu BRAF inhibitor-resistant (451Lu-BR3) (J) and WM983B and WM983B BRAF inhibitor-

resistant (WM983B-BR) (K) over 24 h (n=3, shown also by individual data points). (L) Representative images of 451Lu wild-type (grey frame), 451Lu-BR3 (lighter green frame), WM983B wild-type (grey frame) and WM983B-BR (darker green frame) melanoma spheroids after 72 h invasion in collagen. Live cells are shown in green. Scale bar: 100 µm. (M-N) Quantification of 451Lu versus 451Lu-BR3 (M) and WM983B versus WM983B-BR (N) spheroid core size (n≥9, shown also by individual data points). Data are presented as mean ± SEM. Statistical significance was determined using unpaired, two-tailed Student's t-test (shMCU cells were compared to their respective control, shCTRL), (*) $p < 0.05$; (**) $p < 0.01$; (***) $p < 0.005$.

4. In Figure 5, Please perform all these crucial experiments in both cell lines with two different shRNA. Such as measurements of both mitochondrial and cytoplasmic ROS.

We agree that this is important and now measured mitochondrial H_2O_2 and/or glutathione redox potential in both 1205Lu and WM3734 MCU_{A_KD} stable cell lines as well as in the BRAF kinase-resistant cell lines. As seen below, both parameters were diminished in the newly measured cells thus confirming the findings of the original manuscript.

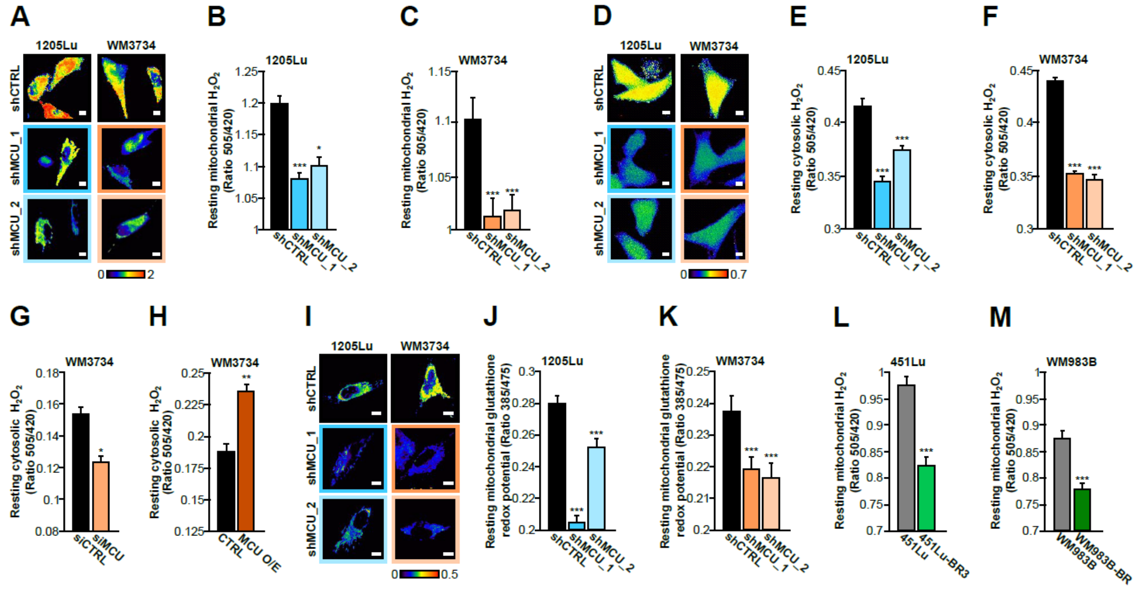

New Figure 5A-M. (A-C) Mitochondrial hydrogen peroxide (H_2O_2) measurement in 1205Lu and WM3734 with and without stable MCU_{A_KD} using mito-HyPer. Exemplary ratiometric images (F505 nm/F420 nm) are shown for all conditions (A) Scale bar: 10 µm. Quantification of mito-HyPer ratio in 1205Lu (B) and WM3734 (C) under resting state (1205Lu: shCTRL=217; shMCU_1=197; shMCU_2=212; WM3734: shCTRL=104; shMCU_1=107; shMCU_2=114). (D-F) Cytosolic hydrogen peroxide (H_2O_2) measurement in 1205Lu and WM3734 with and without stable MCU_{A_KD} using HyPer. Exemplary ratiometric images (F505 nm/F420 nm) are shown for all conditions (D) Scale bar: 10 µm. Quantification of HyPer ratio in 1205Lu (E) and WM3734 (F) under resting state (1205Lu: shCTRL=232; shMCU_1=290; shMCU_2=290; WM3734: shCTRL=443; shMCU_1=297; shMCU_2=233). (G-H) Quantification of H_2O_2 levels in WM3734 upon siRNA-mediated MCU_A depletion (siCTRL=58; siMCU=68) (G) and MCU_A overexpression (O/E) (CTRL=52; MCU O/E=66) (H). (I-K) Mitochondrial glutathione redox potential, measured with mito-Grx1-roGFP2 in 1205Lu and WM3734 with and without stable MCU_{A_KD}. Representative ratiometric images (F385 nm/F475 nm) are shown for all conditions (I). Quantification in 1205Lu (J) and WM3734 (K) under resting state (1205Lu: shCTRL=231; shMCU_1=176; shMCU_2=164; WM3734: shCTRL=127; shMCU_1=142; shMCU_2=116). (L-M) Resting mitochondrial hydrogen peroxide (H_2O_2) in 451Lu and BRAF inhibitor-resistant 451Lu-BR3 (L) and WM983B and BRAF inhibitor-resistant WM983B-BR (M) (451Lu=227; 451Lu-BR3=166; WM983B=193; WM983B-BR=208). All data are presented as mean ± SEM. (A-F) and (I-M) were measured in Ringer's buffer containing 0.5 mM Ca^{2+} and (G-H) in Ringer's buffer containing 1 mM Ca^{2+}. Statistical significance was determined using unpaired, two-tailed Student's t-test, (*) $p < 0.05$; (**) $p < 0.01$; (***) $p < 0.005$.

5. In Figure 5, The ROS levels in cytoplasm and mitochondria should be determined after the antioxidant treatment.

We agree that this is an important control. In a previous study, the effects of NAC, catalase and DTT on cytosolic H_2O_2 have been evaluated and are presented in Fig. EV3M (Zhang et al. EMBO J, 2019). As depicted in the figure R6 (left panel) below, all agents significantly reduced the cytosolic HyPer signals. In addition, we measured glutathione redox potential and found DTT efficiently suppresses the GSH/GSSG signals in 1205Lu cells (R6, right panel). Moreover, we treated both 1205Lu and WM3734 cells with antioxidants such as NAC and mitoTEMPO and determined mitochondrial H_2O_2 levels. As depicted in New Figure S4J-K, both antioxidant compounds suppressed mitochondrial H_2O_2 levels in both cell lines.

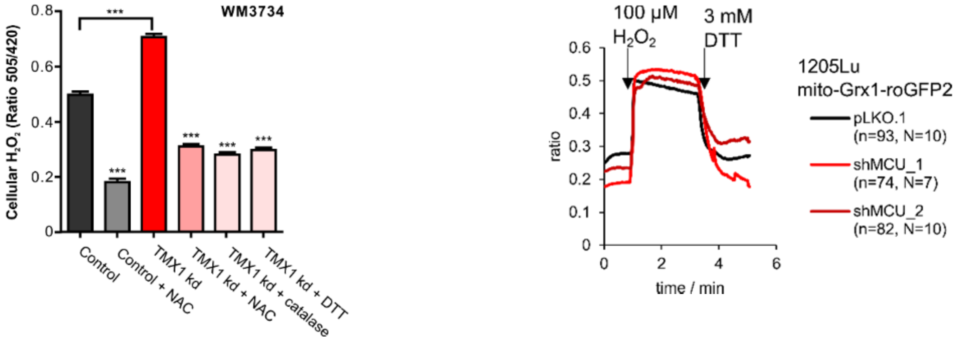

Figure R6. Antioxidants suppress HyPer and Grx1-roGFP2 signals. (left) Cellular H_2O_2 measured with HyPer 25 min after treatment with antioxidants (NAC: 100 µM; catalase: 50 U/ml; DTT 2 mM) (from Zhang et al. EMBO J, 2019). **(right)** Mitochondrial redox potential was measured using mito-Grx1-roGFP2 in 1205Lu cells after stable MCU knockdown. The sensor was fully oxidized by addition of 100 µM H_2O_2 and reduced by addition of 3 mM DTT.

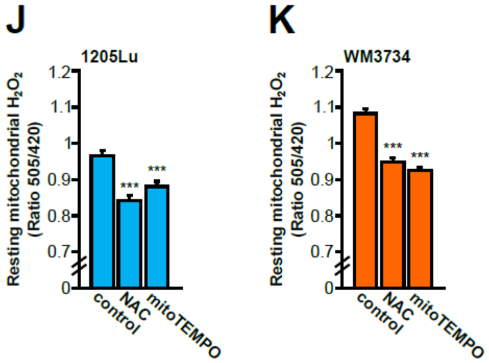

New Figure S4J-K. Resting mitochondrial hydrogen peroxide (H_2O_2) in 1205Lu and WM3734 with and without over-night pre-treatment with NAC (200 µM) or mitoTEMPO (1 µM) (1205Lu: control=191; NAC=148; mito-TEMPO=133; WM3734: control=237; NAC=263; mitoTEMPO=255). Statistical significance was determined using paired, one-tailed Student's t-test, (*) $p < 0.05$; (**) $p < 0.01$; (***) $p < 0.005$.

6. In Figure 5, Reduced MCU function leads to increased mitochondrial and cytosolic ROS. Using a generic antioxidant does not distinguish if the phenotype is due to mtROS of cytoROS. Rescue experiments, migration, and invasion should also be performed using mitoTEMPO. This will determine the role of mitoROS in the phenotype.

In Figure 5 we show that reduced MCU function leads to decreased and not increased mitochondrial and cytosolic ROS. Rescue experiments with mitoTEMPO or other antioxidants would thus not be very helpful in this context.

7. In Figure 5, The viability after drug treatment should be performed in both cell lines.

We agree but we guess that the reviewer refers to the old Fig. 6. We now measured the effect of drug treatment on the 1205Lu cell as suggested. New Figure S5 below indicates that similarly as in WM3734 cells, MCU_{A_KD} does not affect cell sensitivity towards targeted therapies such as vemurafenib and trametinib. Moreover, the treatment with the ferroptosis inducer RSL3 indicated that the 1205Lu_MCUa_KD cells are also more resistant to ferroptosis. Of note, due to the high sensitivity of the 1205Lu cell line to RSL3 we had to use lower concentrations which hindered the direct comparison with the WM3734 cells.

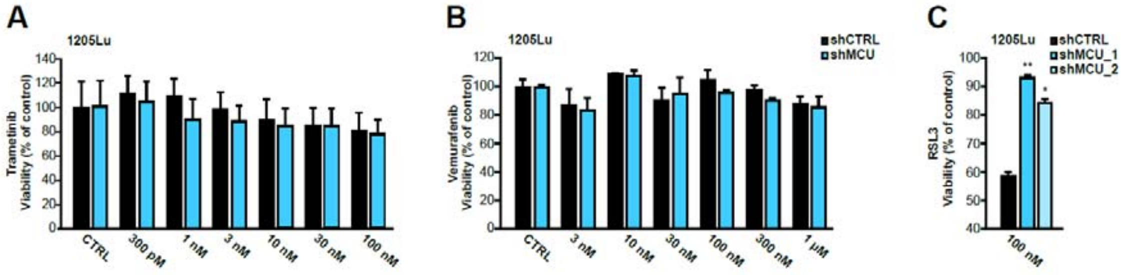

New Figure S5A-C. (A-B) 1205Lu shCTRL and MCU_{A_KD} cell viability following incubation with different concentrations of trametinib for 144 h (A) and vemurafenib for 96 h (B). (C) 1205Lu cell viability upon incubation with 100 µM and RSL3 for 72 h. Data are shown as percent of control (untreated cells, not shown) (n≤8). Abbreviations: RSL = RAS-selective lethal. Data are presented as mean ± SEM. Statistical significance was determined using unpaired, two-tailed Student's t-test, (*) $p < 0.05$; (**) $p < 0.01$; (***) $p < 0.005$.

8. There is no mechanism suggested to explain why reduced mtCa2+ is causing reduced overall ROS in cells. How lower ROS level is causing increased migration and invasion is not determined. How the ROS levels affect the therapeutic sensitivity and metastatic spread is also not clear. In Figures 4 and 6, there are multiple pathways and proteins that are altered due to the reduced function of MCU, which can explain the phenotype. But none of the pathways are validated. Furthermore, mitochondrial structure and function should be determined in both the cell lines and knockdown conditions. That might also help explain the reduced ROS phenotype. Without some of these insights, the scheme in figure 7 is not supported by data, and the manuscript remains preliminary.

Previous studies have determined that mitochondrial Ca^{2+} controls mitochondrial ROS production and most of these studies agree that this is due to Ca^{2+} regulation of dehydrogenases within the mitochondrial matrix and their effect on the electron transfer chain (higher electron transfer rate = higher ROS). Furthermore, several recent studies described mechanisms by which antioxidants and reducing environments promote melanoma migration and metastatic spread, *in vitro* and *in vivo*. Most of these papers are referenced in the current version of the manuscript. We will discuss these papers in more detail in order to make these two important points clearer to the reader.

We would like to mention that redox metabolism was one of the pathways which was strongly affected by MCU_{A_KD}. Accordingly, we validated its involvement. Nevertheless, we agree that

additional knowledge regarding the impact of MCU_A downregulation on mitochondrial function might provide very important insights. We thank the reviewer for this important suggestion.

Our new findings summarized in New Figure 6 show that MCU_{A_KD} causes reduced ATP production and depolarization of the IMM.

As suggested by the reviewer, we analyzed mitochondrial structure using confocal and electron microscopy. The new data showed no overt changes in mitochondrial structure and volume. Accordingly, we concluded that the MCU_{A_KD}-induced effects on melanoma cell phenotype are rather controlled by functional alterations.

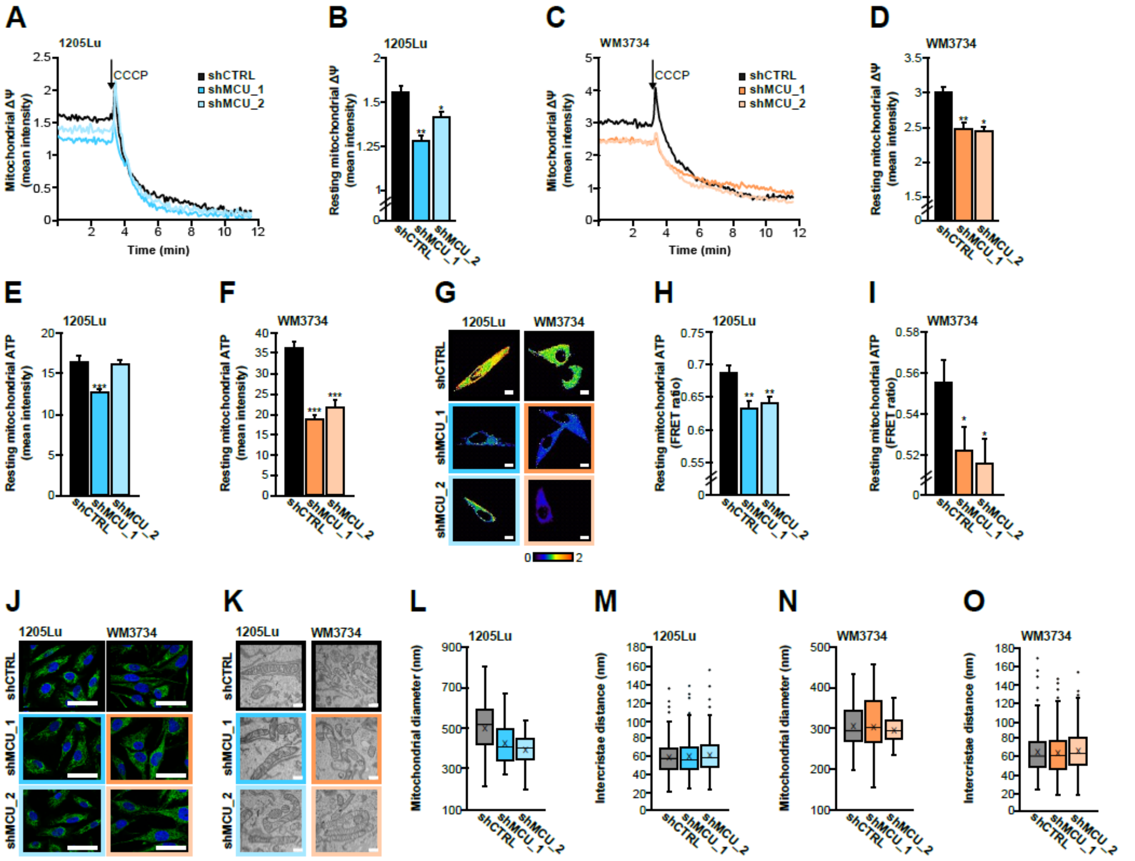

New Figure 6. MCU_A controls mitochondrial ATP production.
(A-D) Resting mitochondrial membrane potential (ΔΨ), measured with TMRE in 1205Lu (A-B) and WM3734 (C-D) with and without stable MCU_{A_KD} (1205Lu: shCTRL=81; shMCU_1=81; shMCU_2=156; WM3734: shCTRL=108; shMCU_1=73; shMCU_2=110). (E-F) Resting mitochondrial ATP levels, measured using the ATP-Red dye in 1205Lu (E) and WM3734 (F) with and without stable MCU_{A_KD} (1205Lu: shCTRL=67; shMCU_1=80; shMCU_2=69; WM3734: shCTRL=40; shMCU_1=38; shMCU_2=21). (G-I) Mitochondrial ATP, measured using mito-ATEAM in 1205Lu and WM3734 with and without stable MCU_{A_KD}. Exemplary ratiometric images (FRET/CFP) are shown for all conditions (G). Scale bar: 10 µm. Quantification of basal levels in 1205Lu (H) and WM3734 (I) (1205Lu: shCTRL=141; shMCU_1=143; shMCU_2=143; WM3734: shCTRL=122; shMCU_1=103; shMCU_2=99). (J) Exemplary confocal microscope images of the mitochondrial network (blue: DAPI staining of the nucleus; green: TOMM20 staining of mitochondria). Scale bar: 50 µm. (K-O) Electron microscopy of mitochondria of stable MCU_{A_KD} cell lines. (K) Exemplary images. Scale bar: 500 nm. (L-O) Quantification of mitochondrial diameter (L and N) and intercristae disctance (M and O) of 1205Lu (L and M) and WM3734 (N and O) cells with and without stable MCU_{A_KD}, presented as boxplot. The box presents the 25 %- quartile, median and 75 %-quartile, the X represents the mean and the whiskers the minimum

and maximum, outliers are represented as dots (mitochondrial diameter 1205Lu: shCTRL=47; shMCU_1=44; shMCU_2=40; WM3734: shCTRL=35; shMCU_1=52; shMCU_2=38; intercristace distance 1205Lu: shCTRL=330; shMCU_1=285; shMCU_2=255; WM3734: shCTRL=352; shMCU_1=433; shMCU_2=357). Statistical significance was determined using unpaired, two-tailed Student's t-test, (*) $p < 0.05$; (**) $p < 0.01$; (***) $p < 0.005$.

9. All bar graphs should have individual data points. This will help in determining the data distribution and will also self-explain the number of repeats.

We agree and now show individual data points for bar graphs in which the n number is low and SEM high. We now also include n values for all measurements in the figure legends.

Minor Comments-

1. In Figures 2 D and E, please explain why the mtCa2+ influx is only ~40% reduced in WM3734 shMCU even when the MCU protein levels are 95% reduced.

Only in one out of four lines the reduction in mCa^{2+} is around 40-50 % while in the other 3 lines the inhibition is much higher. To further examine this we used ATP stimulation instead of thapsigargin. These additional measurements demonstrated that mitochondrial Ca^{2+} uptake is strongly inhibited in all four melanoma cell lines. We hope that these additional results (New Figure 2K-P) are sufficient to conclude that MCU_{A_KD} induces strong inhibition of the mitochondrial Ca^{2+} uptake in melanoma cells.

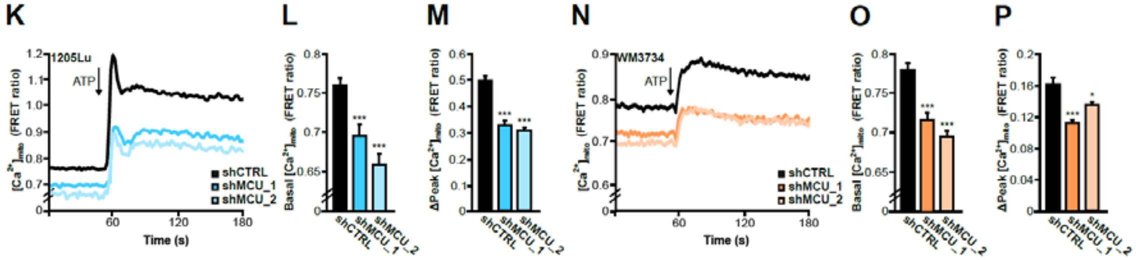

New Figure 2K-P. (K-M) Mitochondrial calcium uptake (represented as FRET ratio) in stable 1205Lu shCTRL (black; n=168), 1205Lu shMCU_1 (darker blue; n=91) and 1205Lu shMCU_2 (lighter blue; n=104) cells upon physiological stimulation with ATP (100 µM). Quantification of basal levels (L) and Ca^{2+} uptake (Δpeak) (M). (N-P) Mitochondrial Ca^{2+} uptake (represented as FRET ratio) in stable WM3734 shCTRL (black; n=147), WM3734 shMCU_1 (darker orange; n=175) and WM3734 shMCU_2 (lighter orange; n=140) cells upon physiological stimulation with ATP (100 µM). Quantification of basal levels (O) and Ca^{2+} uptake (Δpeak) (P). Data were measured in Ringer's buffer containing 0.5 mM Ca^{2+} and are presented as mean ± SEM. Statistical significance was determined using unpaired, two-tailed Student's t-test, (*) $p < 0.05$; (**) $p < 0.01$; (***) $p < 0.005$.

2. In Figure 4H, please write what is the scale bar of the heat map represents and why there is no value for control cells.

Thanks for noticing the missing label. We corrected this. The data was normalized to the CTRL and presented as *Fold change vs CTRL*. This is a routine procedure which is used to quantify RPPA data.

3. In Figure 3G, It is not clear how the invasion of spheroids was measured. The invasion should be measured in 1205LU cells also.

We now provide representative images (Fig. R7) to show how we evaluated the spheroid invasion. We also explain this in more detail in the methods section.

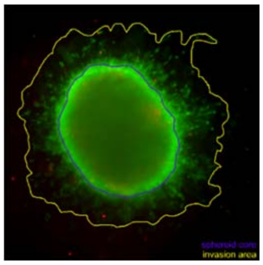

Figure R7. Spheroid invasion evaluation. Spheroid invasion was measured by subtracting the mask for the core of each spheroid (marked with purple) from the total area covered by all the cells of a given spheroid (marked with yellow) [total area – spheroid core] using ImageJ.

4. Please fix the sentence "Cells were injected with the MCUA_KD cells and their" in the melanoma xenograft, under methods on page 22.

Thanks for noticing this typo. We corrected this.

5. Please mention the number of animals used in the main text or figure legends. This issue can also be solved by making graphs with individual data points or scatter blots.

We agree. This will be corrected. 10 mice per group were used (30 mice in total).

1st Revision - Editorial Decision 17th Aug 2022

Dear Prof. Bogeski,

Thank you for the submission of your revised manuscript to our editorial offices. I have now received the reports from the three referees that was asked to re-evaluate your study, you will find below. As you will see, the referees now fully support the publication of your work.

Before proceeding to formal acceptance, I have these editorial requests I ask you to address in a final revised manuscript:

- Please reduce the number of keywords to 5.

- Please make sure that the number "n" for how many independent experiments were performed, their nature (biological versus technical replicates), the bars and error bars (e.g. SEM, SD) and the test used to calculate p-values is indicated in the respective figure legends (main and EV figures), and that statistical testing has been done where applicable. Please avoid phrases like 'independent experiment', but clearly state if these were biological or technical replicates. Please add complete statistical testing to all diagrams (main, EV and Appendix figures). Please also indicate (e.g. with n.s.) if testing was performed, but the differences are not significant.

- It seems there is no callout for Fig. 1G. Please check.

- Please make sure that all the funding information is also entered into the online submission system and that it is complete and similar to the one in the acknowledgement section of the manuscript text file.

- As they are significantly cropped, please provide the source data for the few Western blots shown in the manuscript. The source data will be published in separate source data files online along with the accepted manuscript and will be linked to the relevant figures. Please submit scans of entire gels or blots together with the final revised manuscript. Please include size markers for scans of entire gels, label the scans with figure and panel number, and send one PDF file per figure.

- Please remove the list of EV items at the end of the manuscript text.

- For the dataset files, please add a title and a legend on the first TAB of the excel files and upload these again.

- Please also use our reference format ('et al' for more than 10 authors):
http://www.embopress.org/page/journal/14693178/authorguide#referencesformat

- Finally, please find attached a word file of the manuscript text (provided by our publisher) with changes we ask you to include in your final manuscript text, and some queries, we ask you to address. Please provide your final manuscript file with track changes, in order that we can see any modifications done.

In addition, I would need from you:
- a short, two-sentence summary of the manuscript (not more than 35 words).
- two to four short bullet points highlighting the key findings of your study.
- a schematic summary figure (in jpeg or tiff format with the exact width of 550 pixels and a height of not more than 400 pixels) that can be used as a visual synopsis on our website.

I look forward to seeing the final revised version of your manuscript when it is ready. Please let me know if you have questions regarding the revision.

Achim Breiling
Senior Editor
EMBO Reports

Referee #1:

The authors provided additional experiments and comments that significantly improved the manuscript and addressed most of my concerns. Although I accept the authors reasoning of not testing effect of knockdown of MCU on non-metastatic melanoma cells to determine whether this will increase their metastatic potential, this could have provided further support to their model. Therefore, overall I find the authors response satisfactory and have no further concerns.

Referee #2:

The authors have addressed all my previous concerns. this paper should now be acceptable for publication.

Referee #3:

The authors have gone to great lengths to address the comments from the previous round of review. In this revision, they provide new experiments depicting new data requested by reviews and also provide clarifications -when needed- to various comments from the review. The result is a responsive and strong revision that has greatly enhanced the quality of the manuscript. I have no further comments.

POINT-BY-POINT REPLY

Dear Dr. Breiling,

Thanks a lot for the positive evaluation of our study and the highly professional guidance throughout the whole process.

Thank you for the submission of your revised manuscript to our editorial offices. I have now received the reports from the three referees that was asked to re-evaluate your study, you will find below. As you will see, the referees now fully support the publication of your work.

We are delighted to read that the reviewers support publication of our work and have no further comments.

Before proceeding to formal acceptance, I have these editorial requests I ask you to address in a final revised manuscript:

- Please reduce the number of keywords to 5.

This has been done

- Please make sure that the number "n" for how many independent experiments were performed, their nature (biological versus technical replicates), the bars and error bars (e.g. SEM, SD) and the test used to calculate p-values is indicated in the respective figure legends (main and EV figures), and that statistical testing has been done where applicable. Please avoid phrases like 'independent experiment', but clearly state if these were biological or technical replicates. Please add complete statistical testing to all diagrams (main, EV and Appendix figures). Please also indicate (e.g. with n.s.) if testing was performed, but the differences are not significant.

We now provide information about the number of biological and technical repeats in the figure legends. Furthermore, in the figure legends we indicate that statistical testing was performed but the differences were not significant for the datapoints which are not marked with asterisks. We would like to note here that marking these datapoints with n.s. within the figures would have made the graphical presentation not easy and the figures would appear very busy and difficult to follow.

- It seems there is no callout for Fig. 1G. Please check.

This has been corrected.

- Please make sure that all the funding information is also entered into the online submission system and that it is complete and similar to the one in the acknowledgement section of the manuscript text file.

Done

- As they are significantly cropped, please provide the source data for the few Western blots shown in the manuscript. The source data will be published in separate source data files online along with the accepted manuscript and will be linked to the relevant figures. Please submit scans of entire gels or blots together with the final revised manuscript. Please include size markers for scans of entire gels, label the scans with figure and panel number, and send one PDF file per figure.

Done. Unfortunately, for few blots in which we only wanted to confirm the knockdown efficiency, size markers were not included. We hope that this is acceptable, given the purpose of these blots.

- Please remove the list of EV items at the end of the manuscript text.

Done

- For the dataset files, please add a title and a legend on the first TAB of the excel files and upload these again.

Done

- Please also use our reference format ('et al' for more than 10 authors): http://www.embopress.org/page/journal/14693178/authorguide#referencesformat

Done

- Finally, please find attached a word file of the manuscript text (provided by our publisher) with changes we ask you to include in your final manuscript text, and some queries, we ask you to address. Please provide your final manuscript file with track changes, in order that we can see any modifications done.

The new text file is provided.

In addition, I would need from you:

- a short, two-sentence summary of the manuscript (not more than 35 words).
- two to four short bullet points highlighting the key findings of your study.
- a schematic summary figure (in jpeg or tiff format with the exact width of 550 pixels and a height of not more than 400 pixels) that can be used as a visual synopsis on our website.

We now provide a two-sentence summary and highlights for our manuscript in a separate word file. The summary figure is also submitted in a JPEG format, as instructed.

2nd Revision - Editorial Decision 29th Aug 2022

Prof. Ivan Bogeski
UMG, University of Göttingen
Physiology
Humboldtallee 23
Göttingen 37073
Germany

Dear Prof. Bogeski,

I am very pleased to accept your manuscript for publication in the next available issue of EMBO reports. Thank you for your contribution to our journal.

At the end of this email I include important information about how to proceed. Please ensure that you take the time to read the information and complete and return the necessary forms to allow us to publish your manuscript as quickly as possible.

As part of the EMBO publication's Transparent Editorial Process, EMBO reports publishes online a Review Process File to accompany accepted manuscripts. As you are aware, this File will be published in conjunction with your paper and will include the referee reports, your point-by-point response and all pertinent correspondence relating to the manuscript.

If you do NOT want this File to be published, please inform the editorial office within 2 days, if you have not done so already, otherwise the File will be published by default [contact: emboreports@embo.org]. If you do opt out, the Review Process File link will point to the following statement: "No Review Process File is available with this article, as the authors have chosen not to make the review process public in this case." Please note that the author checklist will still be published even if you opt out of the transparent process.

Thank you again for your contribution to EMBO reports and congratulations on a successful publication. Please consider us again in the future for your most exciting work.

Yours sincerely,

Achim Breiling
Editor
EMBO Reports

****

THINGS TO DO NOW:

Please note that you will be contacted by Wiley Author Services to complete licensing and payment information. The required 'Page Charges Authorization Form' is available here: https://www.embopress.org/pb-assets/embo-site/er_apc.pdf - please download and complete the form and return to embopressproduction@wiley.com

You will receive proofs by e-mail approximately 2-3 weeks after all relevant files have been sent to our Production Office; you should return your corrections within 2 days of receiving the proofs.

Please inform us if there is likely to be any difficulty in reaching you at the above address at that time. Failure to meet our deadlines may result in a delay of publication, or publication without your corrections.

All further communications concerning your paper should quote reference number EMBOR-2022-54746V3 and be addressed to emboreports@wiley.com.

Should you be planning a Press Release on your article, please get in contact with emboreports@wiley.com as early as possible, in order to coordinate publication and release dates.

EMBO Press Author Checklist

Corresponding Author Name: Ivan Bogeski	
Journal Submitted to: EMBO Reports	
Manuscript Number: EMBOR-2022-54746	

USEFUL LINKS FOR COMPLETING THIS FORM
The EMBO Journal - Author Guidelines
EMBO Reports - Author Guidelines
Molecular Systems Biology - Author Guidelines
EMBO Molecular Medicine - Author Guidelines

Reporting Checklist for Life Science Articles (updated January 2022)
This checklist is adapted from Materials Design Analysis Reporting (MDAR) Checklist for Authors. MDAR establishes a minimum set of requirements in transparent reporting in the life sciences (see Statement of Task: 10.31222/osf.io/9sm4x). Please follow the journal's guidelines in preparing your manuscript.
Please note that a copy of this checklist will be published alongside your article.

Abridged guidelines for figures
1. Data
The data shown in figures should satisfy the following conditions:
- the data were obtained and processed according to the field's best practice and are presented to reflect the results of the experiments in an accurate and unbiased manner.
- ideally, figure panels should include only measurements that are directly comparable to each other and obtained with the same assay.
- plots include clearly labeled error bars for independent experiments and sample sizes. Unless justified, error bars should not be shown for technical replicates.
- if n<5, the individual data points from each experiment should be plotted. Any statistical test employed should be justified.
- Source Data should be included to report the data underlying figures according to the guidelines set out in the authorship guidelines on Data Presentation.

2. Captions
Each figure caption should contain the following information, for each panel where they are relevant:
- a specification of the experimental system investigated (eg cell line, species name).
- the assay(s) and method(s) used to carry out the reported observations and measurements.
- an explicit mention of the biological and chemical entity(ies) that are being measured.
- an explicit mention of the biological and chemical entity(ies) that are altered/varied/perturbed in a controlled manner.
- the exact sample size (n) for each experimental group/condition, given as a number, not a range;
- a description of the sample collection allowing the reader to understand whether the samples represent technical or biological replicates (including how many animals, litters, cultures, etc.).
- a statement of how many times the experiment shown was independently replicated in the laboratory.
- definitions of statistical methods and measures:
  - common tests, such as t-test (please specify whether paired vs. unpaired), simple χ2 tests, Wilcoxon and Mann-Whitney tests, can be unambiguously identified by name only, but more complex techniques should be described in the methods section;
  - are tests one-sided or two-sided?
  - are there adjustments for multiple comparisons?
  - exact statistical test results, e.g., P values = x but not P values < x;
  - definition of 'center values' as median or average;
  - definition of error bars as s.d. or s.e.m.

Please complete ALL of the questions below.
Select "Not Applicable" only when the requested information is not relevant for your study.

Materials

Newly Created Materials	Information included in the manuscript?	In which section is the information available? (Reagents and Tools Table, Materials and Methods, Figures, Data Availability Section)
New materials and reagents need to be available; do any restrictions apply?	Not Applicable	

Antibodies	Information included in the manuscript?	In which section is the information available? (Reagents and Tools Table, Materials and Methods, Figures, Data Availability Section)
For **antibodies** provide the following information:<br>- Commercial antibodies: RRID (if possible) or supplier name, catalogue number and or/clone number<br>- Non-commercial: RRID or citation	Yes	This information is provided in the "Materials and Methods" section, under "Immunolabeling and confocal microscopy" and "Western blots", as well as in the Tables EV4 and EV5.

DNA and RNA sequences	Information included in the manuscript?	In which section is the information available? (Reagents and Tools Table, Materials and Methods, Figures, Data Availability Section)
**Short novel DNA or RNA including primers, probes**: provide the sequences.	Yes	This information is available in the Table EV3.

Cell materials	Information included in the manuscript?	In which section is the information available? (Reagents and Tools Table, Materials and Methods, Figures, Data Availability Section)
**Cell lines:** Provide species information, strain. Provide accession number in repository **OR** supplier name, catalog number, clone number, and/**OR** RRID.	Yes	The cell lines used in this study were provided by Meenhard Herlyn. Most of them are also available for purchase at https://www.rockland.com/categories/cell-lines-and-lysates/cell-lines/.
**Primary cultures:** Provide species, strain, sex of origin, genetic modification status.	Yes	NK cells were obtained from human peripheral blood mononuclear cells (PBMCs) of healthy thrombocyte donors of the local blood bank and were not genetically modified.
Report if the cell lines were recently **authenticated** (e.g., by STR profiling) and tested for mycoplasma contamination.	Yes	Cell lines, gifted by Meenhard Herlyn were authenticated by DNA fingerprinting, using Coriell's microsatellite kit. All cell lines tested negative for Mycoplasma (information provided in the "Materials and Methods" section).

Experimental animals	Information included in the manuscript?	In which section is the information available? (Reagents and Tools Table, Materials and Methods, Figures, Data Availability Section)
**Laboratory animals or Model organisms:** Provide species, strain, sex, age, genetic modification status. Provide accession number in repository **OR** supplier name, catalog number, clone number, **OR** RRID.	Yes	Information briefly provided in the "Materials and methods" section, subsection "Melanoma xenografts". NOD/LtSscidIL2Rynull (NSG) mice were inbred at The Wistar Institute under license from the Jackson Laboratory (00557). Ten mice (female and male) were used per group, randomized.
**Animal observed in or captured from the field:** Provide species, sex, and age where possible.	Not Applicable	
Please detail **housing and husbandry conditions**.	Yes	Information briefly provided in the "Materials and methods" section, subsection "Melanoma xenografts". Mice were kept in the same holding room, handled aseptically in germ-free environment in Biosafety Cabinet (Class II), socially caged as 5 mice/unit cage and fed with sterile food pellets and water. Wistar Animal Facility has a quality control program in place wherein 5 % of mice in each holding room were periodically tested serologically for common murine viruses (Sendai, pneumonia virus of mice, mouse hepatitis virus, theoloviruses, reo virus, lymphocyte choriomeningitis virus, ectromelia virus, mouse pneumonitis virus, polyoma virus, mastadeno virus, mouse rotavirus, mouse parvovirus, and murine Nora virus), mycoplasma pulmonis and helicobacter.

Plants and microbes	Information included in the manuscript?	In which section is the information available? (Reagents and Tools Table, Materials and Methods, Figures, Data Availability Section)
**Plants:** provide species and strain, ecotype and cultivar where relevant, unique accession number if available, and source (including location for collected wild specimens).	Not Applicable	
**Microbes:** provide species and strain, unique accession number if available, and source.	Not Applicable	

Human research participants	Information included in the manuscript?	In which section is the information available? (Reagents and Tools Table, Materials and Methods, Figures, Data Availability Section)
If collected and within the bounds of privacy constraints report on age, sex and gender or ethnicity for all study participants.	Not Applicable	

Core facilities	Information included in the manuscript?	In which section is the information available? (Reagents and Tools Table, Materials and Methods, Figures, Data Availability Section)

If your work benefited from core facilities, was their service mentioned in the acknowledgments section?	Not Applicable	

Design

Study protocol	Information included in the manuscript?	In which section is the information available? (Reagents and Tools Table, Materials and Methods, Figures, Data Availability Section)
If study protocol has been **pre-registered, provide DOI in the manuscript**. For clinical trials, provide the trial registration number **OR** cite DOI.	Not Applicable	
Report the **clinical trial registration number** (at ClinicalTrials.gov or equivalent), where applicable.	Not Applicable	

Laboratory protocol	Information included in the manuscript?	In which section is the information available? (Reagents and Tools Table, Materials and Methods, Figures, Data Availability Section)
Provide DOI OR other citation details if **external detailed step-by-step protocols** are available.	Not Applicable	

Experimental study design and statistics	Information included in the manuscript?	In which section is the information available? (Reagents and Tools Table, Materials and Methods, Figures, Data Availability Section)
Include a statement about **sample size** estimate even if no statistical methods were used.	Not Applicable	
Were any steps taken to minimize the effects of subjective bias when allocating animals/samples to treatment (e.g. **randomization procedure**)? If yes, have they been described?	Not Applicable	
Include a statement about **blinding** even if no blinding was done.	Not Applicable	No blinding was done.
Describe **inclusion/exclusion criteria** if samples or animals were excluded from the analysis. Were the criteria pre-established?<br><br>If sample or data points were omitted from analysis, report if this was due to attrition or intentional exclusion and provide justification.	Not Applicable	
For every figure, are **statistical tests** justified as appropriate? Do the data meet the assumptions of the tests (e.g., normal distribution)? Describe any methods used to assess it. Is there an estimate of variation within each group of data? Is the variance similar between the groups that are being statistically compared?	Yes	Please refer to the "Materials and methods" section, subsection "Statistical analyses".

Sample definition and in-laboratory replication	Information included in the manuscript?	In which section is the information available? (Reagents and Tools Table, Materials and Methods, Figures, Data Availability Section)
In the figure legends: state number of times the experiment was **replicated** in laboratory.	Yes	This information is available for each figure in the figure legend.
In the figure legends: define whether data describe **technical or biological replicates**.	Yes	All data describe biological repeats.

Ethics

Ethics	Information included in the manuscript?	In which section is the information available? (Reagents and Tools Table, Materials and Methods, Figures, Data Availability Section)
Studies involving **human participants**: State details of **authority granting ethics approval** (IRB or equivalent committee(s), provide reference number for approval.	Yes	Please refer to the "Materials and methods" section, subsection "Real-time killing assay".
Studies involving **human participants**: Include a statement confirming that **informed consent** was obtained from all subjects and that the experiments conformed to the principles set out in the WMA Declaration of Helsinki and the Department of Health and Human Services Belmont Report.	Yes	Please refer to the "Materials and methods" section, subsection "Real-time killing assay".
Studies involving **human participants:** For publication of **patient photos**, include a statement confirming that consent to publish was obtained.	Not Applicable	
Studies involving experimental **animals**: State details of **authority granting ethics approval** (IRB or equivalent committee(s), provide reference number for approval. Include a statement of compliance with ethical regulations.	Yes	All animal experiments were performed in accordance with the Wistar Institute's Institutional Animal Care and User Committee (IACUC protocol 201227).
Studies involving **specimen and field samples:** State if relevant **permits** obtained, provide details of authority approving study; if none were required, explain why.	Not Applicable	

Dual Use Research of Concern (DURC)	Information included in the manuscript?	In which section is the information available? (Reagents and Tools Table, Materials and Methods, Figures, Data Availability Section)
Could your study fall under dual use research restrictions? Please check biosecurity documents and list of **select agents and toxins** (CDC): https://www.selectagents.gov/sat/list.htm	Not Applicable	
If you used a select agent, is the security level of the lab appropriate and reported in the manuscript?	Not Applicable	
If a study is subject to dual use research of concern regulations, is the name of the **authority granting approval and reference number** for the regulatory approval provided in the manuscript?	Not Applicable	

Reporting

The MDAR framework recommends adoption of discipline-specific guidelines, established and endorsed through community initiatives. Journals have their own policy about requiring specific guidelines and recommendations to complement MDAR.

Adherence to community standards	Information included in the manuscript?	In which section is the information available? (Reagents and Tools Table, Materials and Methods, Figures, Data Availability Section)
State if relevant guidelines or checklists (e.g., **ICMJE, MIBBI, ARRIVE, PRISMA**) have been followed or provided.	Not Applicable	
For **tumor marker prognostic studies,** we recommend that you follow the **REMARK** reporting guidelines (see link list at top right). See author guidelines, under 'Reporting Guidelines'. Please confirm you have followed these guidelines.	Not Applicable	
For **phase II and III randomized controlled trials**, please refer to the **CONSORT** flow diagram (see link list at top right) and submit the CONSORT checklist (see link list at top right) with your submission. See author guidelines, under 'Reporting Guidelines'. Please confirm you have submitted this list.	Not Applicable	

Data Availability

Data availability	Information included in the manuscript?	In which section is the information available? (Reagents and Tools Table, Materials and Methods, Figures, Data Availability Section)
Have **primary datasets** been deposited according to the journal's guidelines (see 'Data Deposition' section) and the respective accession numbers provided in the Data Availability Section?	Yes	Please refer to the "Data availability" section in the manuscript.
Were **human clinical and genomic datasets** deposited in a public access-controlled repository in accordance to ethical obligations to the patients and to the applicable consent agreement?	Not Applicable	
Are **computational models** that are central and integral to a study available without restrictions in a machine-readable form? Were the relevant accession numbers or links provided?	Not Applicable	
If publicly available data were reused, provide the respective **data citations in the reference list**.	Yes	Please refer to the "Materials and methods" section, subsection "Bioinformatic analyses" in the manuscript.
